# Human candidate gene polymorphisms and risk of severe malaria in children in Kilifi, Kenya: a case-control association study

**DOI:** 10.1016/S2352-3026(18)30107-8

**Published:** 2018-07-20

**Authors:** Carolyne M Ndila, Sophie Uyoga, Alexander W Macharia, Gideon Nyutu, Norbert Peshu, John Ojal, Mohammed Shebe, Kennedy O Awuondo, Neema Mturi, Benjamin Tsofa, Nuno Sepúlveda, Taane G Clark, Gavin Band, Geraldine Clarke, Kate Rowlands, Christina Hubbart, Anna Jeffreys, Silvia Kariuki, Kevin Marsh, Margaret Mackinnon, Kathryn Maitland, Dominic P Kwiatkowski, Kirk A Rockett, Thomas N Williams, MalariaGEN Consortium, Amadou Abathina, Ismaela Abubakar, Eric Achidi, Tsiri Agbenyega, Mohammed Aiyegbo, Alex Akoto, Angela Allen, Stephen Allen, Lucas Amenga-Etego, Folakemi Amodu, Olukemi Amodu, Judith Anchang-Kimbi, Nana Ansah, Patrick Ansah, Daniel Ansong, Sampson Antwi, Thomas Anyorigiya, Tobias Apinjoh, Emmanuel Asafo-Agyei, Victor Asoala, Frank Atuguba, Sarah Auburn, Abdou Bah, Kariatou Bamba, Germana Bancone, Gavin Band, David Barnwell, Abdoulaye Barry, Evasius Bauni, Richard Besingi, Kalifa Bojang, Edith Bougouma, Susan Bull, George Busby, Abdoulie Camara, Landing Camara, Susana Campino, Richard Carter, Dan Carucci, Climent Casals-Pascual, Ndey Ceesay, Pa Ceesay, Tran Chau, Ly Chuong, Taane Clark, Geraldine Clarke, Ramou Cole-Ceesay, David Conway, Katharine Cook, Olivia Cook, Victoria Cornelius, Patrick Corran, Simon Correa, Sharon Cox, Rachel Craik, Bakary Danso, Timothy Davis, Nicholas Day, Panos Deloukas, Awa Dembele, Jantina deVries, Rajika Dewasurendra, Mahamadou Diakite, Elizabeth Diarra, Yaya Dibba, Andrea Diss, Abdoulaye Djimdé, Amagana Dolo, Ogobara Doumbo, Alan Doyle, Chris Drakeley, Eleanor Drury, Patrick Duffy, Sarah Dunstan, Augustine Ebonyi, Ahmed Elhassan, Ibrahim Elhassan, Abier Elzein, Anthony Enimil, Pamela Esangbedo, Jennifer Evans, Julie Evans, Jeremy Farrar, Deepika Fernando, Kathryn Fitzpatrick, Janet Fullah, Jacob Garcia, Anita Ghansah, Michael Gottleib, Angie Green, Lee Hart, Meike Hennsman, Tran Hien, Nguyen Hieu, Eliza Hilton, Abraham Hodgson, Rolf Horstmann, Christina Hubbart, Catherine Hughes, Ayman Hussein, Robert Hutton, Muntaser Ibrahim, Deus Ishengoma, Jula Jaiteh, Mariatou Jallow, Muminatou Jallow, Kebba Jammeh, Momodou Jasseh, Anna Jeffreys, Amie Jobarteh, Kimberly Johnson, Sarah Joseph, Dushyanth Jyothi, David Kachala, Dorcas Kamuya, Haddy Kanyi, Harin Karunajeewa, Nadira Karunaweera, Momodou Keita, Angeliki Kerasidou, Aja Khan, Katja Kivinen, Gilbert Kokwaro, Amadou Konate, Salimata Konate, Kwadwo Koram, Dominic Kwiatkowski, Moses Laman, Si Le, Ellen Leffler, Martha Lemnge, Enmoore Lin, Alioune Ly, Alexander Macharia, Bronwyn MacInnis, Nguyen Mai, Julie Makani, Cinzia Malangone, Valentina Mangano, Alphaxard Manjurano, Lamin Manneh, Laurens Manning, Magnus Manske, Kevin Marsh, Vicki Marsh, Gareth Maslen, Caroline Maxwell, Eric Mbunwe, Marilyn McCreight, Daniel Mead, Alieu Mendy, Anthony Mendy, Nathan Mensah, Pascal Michon, Alistair Miles, Olivo Miotto, David Modiano, Hiba Mohamed, Sile Molloy, Malcolm Molyneux, Sassy Molyneux, Mike Moore, Catherine Moyes, Frank Mtei, George Mtove, Ivo Mueller, Regina Mugri, Annie Munthali, Theonest Mutabingwa, Behzad Nadjm, Andre Ndi, Carolyne Ndila, Charles Newton, Amadou Niangaly, Haddy Njie, Jalimory Njie, Madi Njie, Malick Njie, Sophie Njie, Labes Njiragoma, Francis Nkrumah, Neema Ntunthama, Aceme Nyika, Vysaul Nyirongo, John O'Brien, Herbert Obu, Abraham Oduro, Alex Ofori, Subulade Olaniyan, Rasaq Olaosebikan, Tom Oluoch, Olayemi Omotade, Olajumoke Oni, Emmanuel Onykwelu, Daniel Opi, Adebola Orimadegun, Sean O'Riordan, Issa Ouedraogo, Samuel Oyola, Michael Parker, Richard Pearson, Paul Pensulo, Norbert Peshu, Ajib Phiri, Nguyen Phu, Margaret Pinder, Matti Pirinen, Chris Plowe, Claire Potter, Belco Poudiougou, Odile Puijalon, Nguyen Quyen, Ioannis Ragoussis, Jiannis Ragoussis, Oba Rasheed, John Reeder, Hugh Reyburn, Eleanor Riley, Paul Risley, Kirk Rockett, Joanne Rodford, Jane Rogers, William Rogers, Kate Rowlands, Valentín Ruano-Rubio, Kumba Sabally-Ceesay, Abubacar Sadiq, Momodou Saidy-Khan, Horeja Saine, Anavaj Sakuntabhai, Abdourahmane Sall, David Sambian, Idrissa Sambou, Miguel SanJoaquin, Nuno Sepúlveda, Shivang Shah, Jennifer Shelton, Peter Siba, Nilupa Silva, Cameron Simmons, Jaques Simpore, Pratap Singhasivanon, Dinh Sinh, Sodiomon Sirima, Giorgio Sirugo, Fatoumatta Sisay-Joof, Sibiry Sissoko, Kerrin Small, Elilan Somaskantharajah, Chris Spencer, Jim Stalker, Marryat Stevens, Prapat Suriyaphol, Justice Sylverken, Bintou Taal, Adama Tall, Terrie Taylor, Yik Teo, Cao Thai, Mahamadou Thera, Vincent Titanji, Ousmane Toure, Marita Troye-Blomberg, Stanley Usen, Sophie Uyoga, Aaron Vanderwal, Hannah Wangai, Renee Watson, Thomas Williams, Michael Wilson, Rebecca Wrigley, Clarisse Yafi, Lawrence Yamoah

**Affiliations:** a KEMRI/Wellcome Trust Research Programme, Kilifi, Kenya; b Wellcome Centre for Human Genetics, University of Oxford, Oxford, UK; c Big Data Institute, University of Oxford, Oxford, UK; d London School of Hygiene & Tropical Medicine, London, UK; e Centro de Estatística e Aplicações da Universidade de Lisboa, Lisbon, Portugal; f Wellcome Sanger Institute, Cambridge, UK; g Department of Medicine, Imperial College, St Mary's Hospital, London, UK; h unaffiliated researcher [ResearcherID: L-3155-2013]

## Abstract

**Background:**

Human genetic factors are important determinants of malaria risk. We investigated associations between multiple candidate polymorphisms—many related to the structure or function of red blood cells—and risk for severe *Plasmodium falciparum* malaria and its specific phenotypes, including cerebral malaria, severe malaria anaemia, and respiratory distress.

**Methods:**

We did a case-control study in Kilifi County, Kenya. We recruited as cases children presenting with severe malaria to the high-dependency ward of Kilifi County Hospital. We included as controls infants born in the local community between Aug 1, 2006, and Sept 30, 2010, who were part of a genetics study. We tested for associations between a range of candidate malaria-protective genes and risk for severe malaria and its specific phenotypes. We used a permutation approach to account for multiple comparisons between polymorphisms and severe malaria. We judged p values less than 0·005 significant for the primary analysis of the association between candidate genes and severe malaria.

**Findings:**

Between June 11, 1995, and June 12, 2008, 2244 children with severe malaria were recruited to the study, and 3949 infants were included as controls. Overall, 263 (12%) of 2244 children with severe malaria died in hospital, including 196 (16%) of 1233 with cerebral malaria. We investigated 121 polymorphisms in 70 candidate severe malaria-associated genes. We found significant associations between risk for severe malaria overall and polymorphisms in 15 genes or locations, of which most were related to red blood cells: *ABO, ATP2B4, ARL14, CD40LG, FREM3, INPP4B, G6PD, HBA* (both *HBA1* and *HBA2*), *HBB, IL10, LPHN2* (also known as *ADGRL2*), *LOC727982, RPS6KL1, CAND1*, and *GNAS*. Combined, these genetic associations accounted for 5·2% of the variance in risk for developing severe malaria among individuals in the general population. We confirmed established associations between severe malaria and sickle-cell trait (odds ratio [OR] 0·15, 95% CI 0·11–0·20; p=2·61 × 10^−58^), blood group O (0·74, 0·66–0·82; p=6·26 × 10^−8^), and –α^3·7^-thalassaemia (0·83, 0·76–0·90; p=2·06 × 10^−6^). We also found strong associations between overall risk of severe malaria and polymorphisms in both *ATP2B4* (OR 0·76, 95% CI 0·63–0·92; p=0·001) and *FREM3* (0·64, 0·53–0·79; p=3·18 × 10^−14^). The association with *FREM3* could be accounted for by linkage disequilibrium with a complex structural mutation within the glycophorin gene region (comprising *GYPA, GYPB*, and *GYPE*) that encodes for the rare Dantu blood group antigen. Heterozygosity for Dantu was associated with risk for severe malaria (OR 0·57, 95% CI 0·49–0·68; p=3·22 × 10^−11^), as was homozygosity (0·26, 0·11–0·62; p=0·002).

**Interpretation:**

Both *ATP2B4* and the Dantu blood group antigen are associated with the structure and function of red blood cells. *ATP2B4* codes for plasma membrane calcium-transporting ATPase 4 (the major calcium pump on red blood cells) and the glycophorins are ligands for parasites to invade red blood cells. Future work should aim at uncovering the mechanisms by which these polymorphisms can result in severe malaria protection and investigate the implications of these associations for wider health.

**Funding:**

Wellcome Trust, UK Medical Research Council, European Union, and Foundation for the National Institutes of Health as part of the Bill & Melinda Gates Grand Challenges in Global Health Initiative.

## Introduction

*Plasmodium falciparum* malaria has had a pre-eminent role in child mortality within tropical regions for the past 5000 years. This mosquito-transmitted infection spends most of its lifecycle in human beings within red blood cells, in which it multiplies approximately ten times every 2 days until controlled by either immunity or treatment. Chronic asymptomatic and repeated uncomplicated malaria episodes cause high levels of childhood anaemia and undernutrition in malaria-endemic areas, whereas severe malaria—including severe malarial anaemia, cerebral malaria, and respiratory distress—is associated with high acute mortality.^1^

**Research in context**

**Evidence before this study**

We searched PubMed between Jan 1, 1960, and May 30, 2018, with the terms (“malaria” AND “case-control”), (“malaria” AND “*ATP2B4*”), (“malaria” AND “*FREM*3”), and “Dantu”. We did not restrict our search by language. We supplemented our search with a review of reference lists within papers identified by our search and those from our personal records. Our search retrieved six papers containing data for malaria and *ATP2B4* (four case-control studies, one population genetic study, and one review article), two relevant papers relating to malaria and *FREM3* (one case-control study and one molecular genetic study), and one laboratory-based functional study relating to malaria and the rare blood group antigen Dantu. Although previous studies have highlighted associations between polymorphisms in *ATP2B4* and *FREM3* and malaria risk, they have lacked detail about the precise clinical effects of these polymorphisms. Moreover, previous studies have not confirmed that the association between *FREM3* and severe malaria is accounted for by close chromosomal linkage to the rare blood group antigen Dantu.

**Added value of this study**

We report the results of a large case-control study (n=6193) of severe *Plasmodium falciparum* malaria, undertaken in a carefully phenotyped population. We have described the effects of both *ATP2B4* and Dantu on specific phenotypes of severe malaria, on parasite densities during malaria episodes, on haematological indices, and on death during hospital admission. Moreover, in all participants we genotyped 121 polymorphisms in 70 candidate genes and could put associations into context with other candidate genes and quantify their overall effect on disease susceptibility. Presence of the Dantu mutation was associated with reductions in risk for severe malaria overall—43% among heterozygotes and 74% among homozygotes. Protection was equal for all forms of severe malaria and Dantu also protected against malaria-related death. Similarly, *ATP2B4* was strongly protective against all forms of severe malaria but, moreover, was associated with lower parasite densities.

**Implications of all the available evidence**

Both the Dantu and *ATP2B4* polymorphisms affect the membrane of red blood cells, which is the main target of malaria infection in human beings, yet the haematological outcomes of these polymorphisms remain unknown. In the case of the *Plasmodium vivax* form of human malaria, discovery of a strong protective association between negativity for the red cell blood group Duffy antigen has led directly to design of a promising vaccine. Discovering the mechanisms by which *ATP2B4* and Dantu protect against severe *P falciparum* malaria offers similar potential for drug and vaccine development in the future.

Malaria has had a major effect on the human genome through selection of polymorphisms associated with improved survival.^2^ Some of these polymorphisms include the classic red-blood-cell variants sickle-cell trait and α^+^-thalassaemia. However, protection against malaria might also be afforded by other polymorphisms.^3^

Here, we describe the effect of a wide range of key genetic candidates—most of which relate to the structure and function of red blood cells—on risk for severe childhood malaria in the population of Kilifi in Kenya. The aim of our study was to verify the importance of specific candidates that could provide insights into the pathophysiology of severe malaria and that might be plausibly used to underpin the development of new approaches to prevention and treatment of this debilitating disease.

## Methods

### Study design and participants

Our study was set in Kilifi County on the coast of Kenya.^4^ We investigated the effect of a range of candidate genes on risk for severe falciparum malaria, using a case-control approach.

We included as cases children (aged <14 years) who presented to hospital with clinical features of severe malaria^5^, ^6^, ^7^ and were subsequently admitted to the high-dependency ward of Kilifi County Hospital. We excluded children who presented before or after the recruitment dates and those without specific features of severe malaria. We focused our study on children, because in the context of most countries within sub-Saharan Africa, severe malaria is almost exclusively restricted to childhood.^1^ We categorised cases into the major phenotypes of cerebral malaria, respiratory distress, severe malarial anaemia, and other severe malaria (which included clinical evidence of prostration, hypoglycaemia and hyperparasitaemia), as described elsewhere.^5^, ^6^, ^7^ We also classified cases based on their inpatient survival status. We used these categories on the basis that they are either associated with the highest rates of mortality^7^ or that they would probably have been associated with high mortality in the pretreatment era and, as such, have been strong drivers of Darwinian selection.

We included as controls infants (aged 3–12 months) who were born within the same study area as cases and who were recruits to a cohort study investigating genetic susceptibility to a range of childhood diseases.^8^ We recruited roughly two controls for every case—a ratio we arrived at for pragmatic reasons.

Individual written informed consent was provided by the parents of all study participants. Ethics approval for the study was granted by the Kenya Medical Research Institute and the National Ethical Review Committee in Nairobi, Kenya (SCC1192) and the Oxford Tropical Ethical Review Committee in Oxford, UK (020-06).

### Procedures

We investigated the association between severe malaria and 129 single nucleotide polymorphisms (SNPs) plus one 3·7 kb deletion in 70 gene regions that had been identified as candidates through previous studies^9^ (appendix pp 2–4). We did genotyping with the Sequenom MassARRAY iPLEX platform (Agena Biosciences, Hamburg, Germany;^9^ appendix pp 5, 6), using DNA extracted by proprietary methods (ABI PRISM, Applied Biosystems, Foster City, CA, USA; Qiagen DNA Blood Mini Kit, Qiagen, Crawley, UK) from fresh or frozen samples of whole blood obtained from participants. We genotyped samples for the common African form of α^+^-thalassaemia (3·7 kb deletion in *HBA1* and *HBA2* [–α^3·7^-thalassaemia]) by PCR, as described previously,^10^, ^11^ and we used a BlpI restriction fragment length polymorphism assay (Thermo Fisher Scientific, Waltham, MA, USA) to type for the rare Dantu blood group antigen polymorphism (known as DUP4, a duplication copy number variant), as described previously.^12^

### Outcomes

The primary outcome was the association between specific genetic polymorphisms and risk of severe *P falciparum* malaria both overall and in terms of specific phenotypes, expressed as odds ratios (ORs) for their frequency in cases compared with healthy uninfected controls. Secondary outcomes included associations between polymorphisms and inpatient mortality, and between polymorphisms and *P falciparum* parasite densities among severe malaria cases, as judged from peripheral blood smears.

### Statistical analysis

We ascertained the sample size for this study pragmatically, as the number of children presenting to Kilifi County Hospital during the study period who were eligible for inclusion as cases. We included roughly two controls for every case to maximise power. We did all analyses using R.

Of the 130 polymorphisms assayed, we removed nine before analysis after standard quality-control procedures. We removed eight SNPs because they were monomorphic and therefore non-contributory to the analysis (*BCAS3* [rs184142841], *GRIP1* [rs192909543], *GUSBP5* [rs148111931], *LTBP2* [rs74063230], *OXNAD1* [rs200704287, rs75180423, and rs79691057], and *RHOG* [rs138826089]) and one SNP was removed because more than 10% of genotypes were missing (*PLEKHG1* [rs144224092]), making genotype calls unreliable (appendix pp 2–4). All remaining polymorphisms passed a test for Hardy-Weinberg equilibrium in the control sample using a threshold of p<0·0001 (this threshold includes a Bonferroni correction for the number of polymorphisms included in this study). Thus, 121 polymorphisms were analysed.

We calculated odds ratios (ORs) for associations of polymorphisms with risk for severe malaria and its major phenotypes, and for disease outcomes in terms of mortality, by comparing allele and genotype frequencies among cases and controls, using a fixed-effects logistic-regression model. We adjusted analyses for the confounding effects of genetic background (using self-reported ethnic origin) and the rs334 polymorphism in *HBB*. rs334 causes both sickle-cell trait and sickle-cell disease, which are major potential confounders in the interpretation of such analyses. Gender is also a known (albeit small) risk factor for malaria, both at a genetic level and at a sociocultural level; therefore, we used gender as a covariate in the autosomal polymorphism analyses. We did not look specifically at the effect of individual ethnic groups in Kilifi because of space and power considerations. In our primary analysis of severe malaria phenotypes, we allowed individual cases to be included more than once if they manifested more than one phenotype of severe malaria. In secondary analyses, we classified individuals uniquely based on relative mortality, using the hierarchy of cerebral malaria, severe malarial anaemia, respiratory distress, and other forms of severe malaria. For X-chromosome polymorphisms, we did analyses both overall and for each gender separately, based on our previous observations with the X-chromosome gene *G6PD*, in which we noted opposing effects in males and females that were undetectable in combined analyses.^13^ In all our analyses, we used a standard meta-analytical approach that utilised fixed effects (inverse-variance weighting) to account for unequal variance.^14^

We tested for associations between polymorphisms and risk for severe malaria using a range of different genetic models of inheritance (including additive, dominant, heterozygous, hemizygous, and recessive) and selected those that were most significant for each polymorphism. However, we analysed the SNP rs8176719 under a recessive model only, because this model was the only one that allowed us to test for an association between severe malaria and blood group O. Doing more than one statistical test can lead to an increased chance of false-positives. We, therefore, used an appropriate significance cutoff (p<0·005), which accounted for the simulated distribution of potential p values, by permuting (randomly reassigning) the case or control status 1000 times using the *SNPassoc* package in R. We then used the lower bound of the 95% CI of the resulting p-value distribution (p≤0·005) as our significance level for all further analyses.^15^ We adjusted p values for gender, ethnic group, and rs334 genotype. Moreover, because epistasis has been reported between several malaria resistance polymorphisms,^16^ we also used a likelihood ratio approach^11^ to test for pairwise interactions between polymorphisms in autosomal chromosomes for which we identified significant associations with severe malaria in our primary analyses.

We assessed the proportion of the total variance in the overall risk of developing severe malaria at least once before the age of 14 years within Kilifi County that could be accounted for both by individual polymorphisms and overall. We analysed data on the raw (binomial), logit-transformed and probit-transformed scales under generalised linear models using the *glm* function in the *stats* package in R, with fixed effects for gender, ethnic group, and the genetic marker or set of markers (appendix pp 26, 27). We calculated estimates of the proportion of the total explained variation (variance) using R^2^ statistics for analyses on the raw scale and McFadden's pseudo-R^2^) statistic when analysed on the transformed scale. We post-adjusted McFadden's pseudo-R^2^) statistic to the underlying disease liability scale, which we assumed to be normally distributed, using the method described by Lee and colleagues.^17^ This approach adjusts for dependency between variances of binomial traits on disease prevalence, thereby enabling comparisons of estimates between populations and studies. Since case-control studies have higher proportions of cases than in the general population (in our study, roughly 20 times higher), we adjusted variance estimates to reflect those in the general population,^17^ based on a designated lifetime prevalence of severe malaria in children younger than 14 years in the general population of 2·15% and the ratio of cases to controls (9:16). We calculated linkage disequilibrium using the *genetics* package in R.

### Role of the funding source

The funders had no role in study design, data collection, data analysis, data interpretation, or writing of the report. CMN, MM, KAR, and TNW had access to raw data. The corresponding author had full access to all data in the study and had final responsibility for the decision to submit for publication.

## Results

Between June 11, 1995, and June 12, 2008, 2244 children presented to the high-dependency ward of Kilifi County Hospital with features of severe falciparum malaria. Between Aug 1, 2006, and Sept 30, 2010, 3949 infants born in the local community were recruited as controls. The clinical, demographic, and laboratory characteristics of severe malaria cases and community controls are summarised in table 1. The flow of cases through the study is shown in the appendix (p 29).

**Table 1:** Clinical, demographic, and laboratory characteristics of study participants

		**Cases**					**Controls (n=3949)**
		Severe malaria (n=2244)	Cerebral malaria (n=1233)	Severe malarial anaemia (n=686)	Respiratory distress (n=686)	Other severe malaria (n=436)	
Age (months)		28·0 (15·0–43·0)	28·0 (16·0–43·0)	19·0 (10·0–33·0)	25·0 (12·7–38·0)	32·0 (19·2–54·0)	6·0 (5·0–8·0)
Male cases		1157 (52%)	627 (51%)	357 (52%)	357 (52%)	226 (52%)	1992 (50%)
Female cases		1087 (48%)	606 (49%)	329 (48%)	329 (48%)	210 (48%)	1957 (50%)
Ethnic origin							
	Giriama	1320 (59%)	743 (60%)	385 (56%)	379 (55%)	268 (62%)	1335 (46%)
	Chonyi	527 (23%)	264 (21%)	176 (26%)	181 (26%)	97 (22%)	1411 (36%)
	Kauma	171 (8%)	98 (8%)	48 (7%)	64 (9%)	25 (6%)	440 (11%)
	Other	226 (10%)	128 (10%)	77 (11%)	62 (9%)	46 (10%)	273 (7%)
Parasite density per μL (geometric mean [95% CI])		40 032 (36 141–44 343)	38 650 (33 608–44 447)	40 944 (34 590–48 465)	56 849 (47 547–67 969)	34 701 (27 111–44 416)	N/A
MCV (fL)		74·1 (9·4)	74·3 (9·3)	75·4 (10·6)	74·8 (9·8)	73·3 (8·9)	N/A
Platelets (× 10^6^ per L)		163 (146)	170 (145)	139 (118)	154 (147)	168 (164)	N/A
Haemoglobin (g/L)		66 (25)	70 (24)	38 (12)	63 (25)	79 (20)	N/A
Inpatient death		263 (12%)	196 (16%)	82 (12%)	119 (17%)	21 (5%)	N/A

Data are median (IQR), number of participants (%), or mean (SD), unless indicated otherwise. Severe malaria cases were classified as cerebral malaria, severe malarial anaemia, and respiratory distress; patients displaying more than one severe malaria phenotype are represented in each of the subgroups. MCV=mean cell volume. N/A=not applicable.

**Figure 1:**
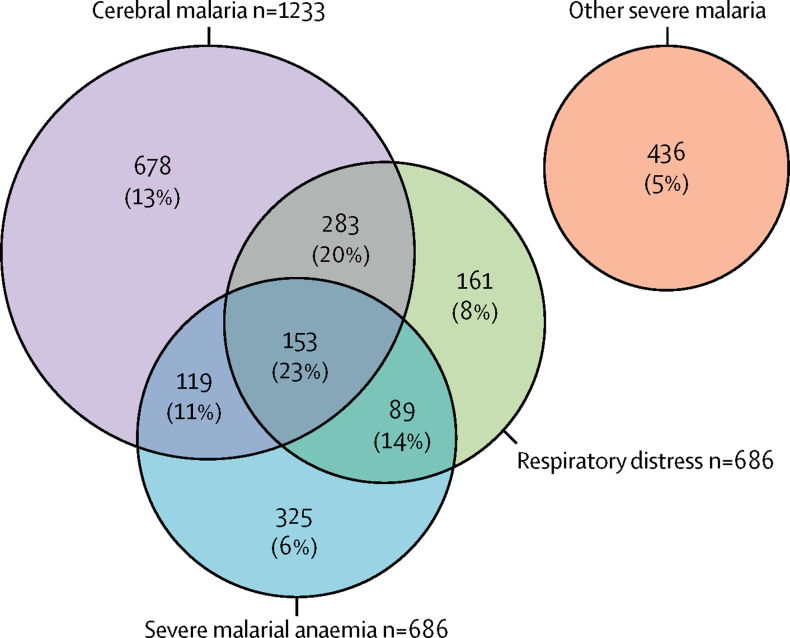
Venn diagram showing the distribution of the three major phenotypes of severe malaria Numbers indicate the cases for each phenotype or co-expressed phenotypes, and percentages are the case-fatality. Other severe malaria cases are those without any of the three phenotypes shown in the main diagram.

Overall, 263 (12%) of 2244 children with severe malaria died in hospital. Mortality was highest in the subgroup with clinical features of cerebral malaria (196 of 1233 [16%]) and lowest in those who manifested other features of severe malaria (21 of 436 [5%]). 644 (29%) cases had two or more common phenotypes of severe malaria (figure 1).

**Figure 2:**
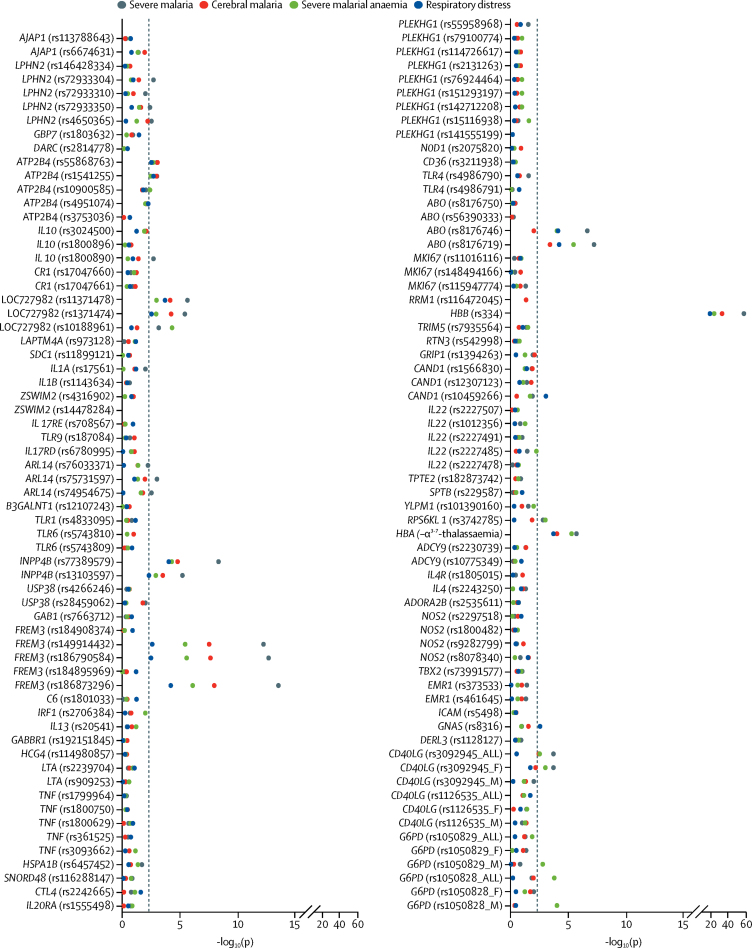
Distribution of p values for association tests with severe malaria phenotypes The minimum p value (−log_10_[p]) for every polymorphism across the models is shown. We adjusted p values for gender, ethnic group, and rs334 genotype. The dashed vertical line represents the significance threshold of p<0·005, which was determined by permutation testing. The polymorphisms tested are listed in chromosomal order (appendix pp 2–4).

The results of association analyses between severe malaria overall and all the polymorphisms under investigation are summarised in figure 2 and in the appendix (pp 7–16). Significant associations were found with severe malaria overall—or with at least one of its phenotypes—for polymorphisms in 15 of 70 genes investigated. The genes and locations were *ABO, ATP2B4, ARL14, CD40LG, FREM3, INPP4B, G6PD, HBA* (both *HBA 1* and *HBA2*), *HBB, IL10, LPHN2* (also known as *ADGRL2*), LOC727982, *RPS6KL1, CAND1*, and *GNAS* for autosomal-related (table 2) and X-chromosome-related (table 3) polymorphisms. Data pertaining to the polymorphisms in *CAND1* and *GNAS* are presented in the appendix (pp 7–16). Analyses done in terms of genotypic groups are summarised in the appendix (p 17). Associations seemed mostly to be additive and pronounced in homozygotes. However, exceptions included *G6PD* and *RPS6KL1*, for which opposite effects were seen in heterozygous and homozygous children, and *IL10*, for which protection was restricted to homozygotes.

**Table 2:** Polymorphisms with significant signals of association in different severe malaria phenotypes

	**Frequency (%) of derived allele in controls (normal/het/hom [n])**	**Frequency (%) of derived allele in cases (normal/het/hom [n])**	Model*	**Unadjusted**		**Adjusted**†	
				OR (95% CI)	p value	OR (95% CI)	p value
***ATP2B4* (rs1541255, chromosome 1; A→G)**							
Severe malaria	33% (1741/1774/418)	31% (1010/1016/185)	R	0·74 (0·62–0·89)	0·001	0·76 (0·63–0·92)	0·001
Cerebral malaria	33% (1741/1774/418)	30% (581/549/96)	R	0·68 (0·54–0·86)	0·001	0·69 (0·54–0·89)	0·001
Severe malarial anaemia	33% (1741/1774/418)	29% (323/293/49)	R	0·63 (0·46–0·87)	0·003	0·60 (0·43–0·84)	0·003
Respiratory distress	33% (1741/1774/418)	30% (322/313/49)	R	0·62 (0·46–0·85)	0·001	0·64 (0·46–0·88)	0·002
Death	33% (1741/1774/418)	31% (119/117/23)	R	0·80 (0·51–1·25)	0·31	0·74 (0·46–1·29)	0·25
***IL10* (rs1800890, chromosome 1; A→T)**							
Severe malaria	24% (2270/1418/257)	23% (1313/821/199)	R	0·69 (0·55–0·88)	0·002	0·72 (0·56–0·93)	0·002
Cerebral malaria	24% (2270/1418/257)	23% (725/443/57)	R	0·74 (0·55–0·99)	0·03	0·76 (0·55–0·99)	0·04
Severe malarial anaemia	24% (2270/1418/257)	24% (394/256/37)	R	0·83 (0·58–1·19)	0·3	0·89 (0·62–1·30)	0·33
Respiratory distress	24% (2270/1418/257)	23% (400/252/33)	R	0·75 (0·51–1·09)	0·12	0·75 (0·51–1·11)	0·12
Death	24% (2270/1418/257)	23% (156/86/16)	R	0·96 (0·57–1·63)	0·9	0·98 (0·72–1·72)	0·97
***LPHN2* (also known as *ADGRL2*; rs72933304, chromosome 1, C→A)**							
Severe malaria	8% (3297/619/26)	7% (1912/296/8)	A	0·80 (0·69–0·92)	0·001	0·83 (0·72–0·96)	0·002
Cerebral malaria	8% (3297/619/26)	7% (1057/168/5)	A	0·82 (0·69–0·98)	0·02	0·86 (0·71–0·99)	0·03
Severe malarial anaemia	8% (3297/619/26)	7% (570/93/3)	A	0·85 (0·68–1·06)	0·15	0·88 (0·70–1·11)	0·17
Respiratory distress	8% (3297/619/26)	7% (587/93/3)	A	0·83 (0·66–1·03)	0·09	0·85 (0·68–1·07)	0·11
Death	8% (3297/619/26)	6% (228/34/0)	A	0·74 (0·51–1·05)	0·08	0·77 (0·53–1·11)	0·12
**LOC727982 (rs1371478, chromosome 2; C→T)**							
Severe malaria	26% (2123/1466/320)	29% (1088/958/163)	H	1·30 (1·17–1·45)	1·90 × 10^−6^	1·35 (1·20–1·51)	2·31 × 10^−6^
Cerebral malaria	26% (2123/1466/320)	29% (606/536/84)	H	1·31 (1·15–1·50)	4·61 × 10^−5^	1·37 (1·19–1·57)	7·19 × 10^−5^
Severe malarial anaemia	26% (2123/1466/320)	30% (318/291/154)	H	1·31 (1·11–1·55)	0·001	1·45 (1·22–1·74)	0·001
Respiratory distress	26% (2123/1466/320)	29% (331/308/43)	H	1·38 (1·17–1·63)	0·0001	1·47 (1·23–1·74)	0·0001
Death	26% (2123/1466/320)	29% (126/116/21)	H	1·34 (1·04–1·73)	0·02	1·42 (1·09–1·86)	0·02
***ARL14* (rs75731597, chromosome 3, A→C)**							
Severe malaria	8% (2957/500/34)	10% (1754/401/18)	H	1·27 (1·10–1·47)	0·001	1·25 (1·07–1·46)	0·001
Cerebral malaria	8% (2957/500/34)	10% (975/222/9)	H	1·25 (1·05–1·49)	0·01	1·25 (1·04–1·51)	0·01
Severe malarial anaemia	8% (2957/500/34)	10% (520/119/7)	H	1·27 (1·02–1·59)	0·03	1·30 (1·07–1·59)	0·04
Respiratory distress	8% (2957/500/34)	12% (546/119/5)	H	1·23 (0·98–1·53)	0·06	1·28 (0·99–1·59)	0·08
Death	8% (2957/500/34)	10% (1754/401/18)	H	1·45 (1·03–2·04)	0·01	1·55 (1·13–2·13)	0·01
***FREM3* (rs186873296, chromosome 4, A→G)**							
Severe malaria	10% (3216/670/40)	6% (1967/233/6)	A	0·57 (0·49–0·66)	4·23 × 10^−14^	0·64 (0·53–0·79)	3·18 × 10^−14^
Cerebral malaria	10% (3216/670/40)	6% (1087/136/2)	A	0·59 (0·49–0·71)	7·16 × 10^−9^	0·63 (0·49–0·79)	1·12 × 10^−8^
Severe malarial anaemia	10% (3216/670/40)	5% (593/69/1)	A	0·55 (0·43–0·70)	4·56 × 10^−7^	0·67 (0·45–0·81)	8·20 × 10^−7^
Respiratory distress	10% (3216/670/40)	5% (611/68/3)	A	0·53 (0·41–0·69)	4·67 × 10^−7^	0·61 (0·44–0·83)	2·70 × 10^−7^
Death	10% (3216/670/40)	6% (228/29/1)	A	0·59 (0·40–0·85)	0·003	0·60 (0·31–0·82)	0·004
***INPP4B* (rs77389579, chromosome 4, G→T)**							
Severe malaria	6% (3407/516/14)	4% (2019/188/2)	A	0·61 (0·51–0·72)	4·48 × 10^−9^	0·64 (0·53–0·77)	4·79 × 10^−9^
Cerebral malaria	6% (3407/516/14)	5% (1113/109/1)	A	0·63 (0·51–0·78)	1·48 × 10^−5^	0·66 (0·52–0·83)	1·64 × 10^−5^
Severe malarial anaemia	6% (3407/516/14)	4% (613/52/1)	A	0·56 (0·42–0·75)	3·57 × 10^−5^	0·60 (0·43–0·82)	5·35 × 10^−5^
Respiratory distress	6% (3407/516/14)	4% (625/58/0)	A	0·58 (0·44–0·78)	8·53 × 10^−5^	0·62 (0·46–0·83)	9·54 × 10^−5^
Death	6% (3407/516/14)	4% (241/19/0)	A	0·50 (0·31–0·80)	0·001	0·48 (0·28–0·80)	0·001
***ABO* (rs8176719, chromosome 9; blood group O *vs* non-blood group O)**‡							
Severe malaria	26% (256/3632)	31% (198/2032)	R	0·73 (0·65–0·81)	3·99 × 10^−9^	0·74 (0·66–0·82)	6·26 × 10^−8^
Cerebral malaria	26% (256/3632)	30% (198/2032)	R	0·77 (0·68–0·88)	9·36 × 10^−5^	0·78 (0·69–0·92)	0·0004
Severe malarial anaemia	26% (256/3632)	32% (198/2032)	R	0·66 (0·56–0·78)	8·45 × 10^−7^	0·67 (0·78–0·79)	3·68 × 10^−6^
Respiratory distress	26% (256/3632)	31% (198/2032)	R	0·70 (0·59–0·83)	2·74 × 10^−5^	0·71 (0·60–0·84)	6·45 × 10^−5^
Death	26% (256/3632)	32% (198/2032)	R	0·67 (0·52–0·87)	0·002	0·67 (0·52–0·87)	0·002
***ABO* (rs8176746, chromosome 9; C→A)**§							
Severe malaria	13% (2970/878/76)	17% (1529/622/76)	A	1·37 (1·22–1·55)	9·60 × 10^−9^	1·38 (1·22–1·50)	2·38 × 10^−7^
Cerebral malaria	13% (2970/878/76)	16% (870/317/36)	A	1·23 (1·06–1·43)	0·005	1·22 (1·04–1·42)	0·01
Severe malarial anaemia	13% (2970/878/76)	18% (459/196/27)	A	1·44 (1·20–1·73)	8·50 × 10^−5^	1·44 (1·19–1·74)	0·0001
Respiratory distress	13% (2970/878/76)	18% (474/178/31)	A	2·55 (1·66–3·92)	1·79 × 10^−5^	2·42 (1·56–3·76)	8·04 × 10^−5^
Death	13% (2970/878/76)	17% (2181/169/8)	A	1·28 (0·96–1·71)	0·08	1·29 (0·96–1·72)	0·08
***HBB* (rs334; chromosome 11; A→T)**							
Severe malaria	8% (3320/596/33)	1% (2159/57/11)	H	0·14 (0·12–0·21)	6·81 × 10^−59^	0·15 (0·11–0·20)	2·61 × 10^−58^
Cerebral malaria	8% (3320/596/33)	2% (1185/29/8)	H	0·14 (0·09–0·21)	8·64 × 10^−39^	0·14 (0·09–0·20)	1·67 × 10^−38^
Severe malarial anaemia	8% (3320/596/33)	2% (666/9/8)	H	0·07 (0·04–0·15)	2·11 × 10^−31^	0·07 (0·03–0·17)	2·61 × 10^−31^
Respiratory distress	8% (3320/596/33)	2% (664/13/7)	H	0·11 (0·06–0·19)	8·41 × 10^−28^	0·11 (0·06–0·20)	1·62 × 10^−27^
Death	8% (3320/596/33)	3% (242/11/3)	H	0·26 (0·14–0·48)	1·96 × 10^−7^	0·26 (0·14–0·49)	2·41 × 10^−7^
***RPS6KL1* (rs3742785, chromosome 14; C→A)**							
Severe malaria	29% (1935/1422/357)	30% (1032/896/187)	H	1·20 (1·08–1·34)	0·001	1·18 (1·05–1·33)	0·001
Cerebral malaria	29% (1935/1422/357)	29% (584/491/95)	H	1·18 (1·03–1·35)	0·01	1·19 (1·03–1·37)	0·01
Severe malarial anaemia	29% (1935/1422/357)	32% (293/292/65)	H	1·33 (1·12–1·57)	0·001	1·37 (1·15–1·64)	0·001
Respiratory distress	29% (1935/1422/357)	28% (341/260/55)	H	1·06 (0·89–1·26)	0·45	1·06 (0·89–1·27)	0·5
Death	29% (1935/1422/357)	26% (140/95/18)	H	0·98 (0·75–1·28)	0·91	0·99 (0·76–1·29)	0·96
***HBA* (−α^3·7^-thalassaemia, chromosome 16; insertion/deletion)**							
Severe malaria	40% (1356/1953/637)	36% (853/1026/264)	A	0·82 (0·76–0·89)	6·59 × 10^−7^	0·83 (0·76–0·90)	2·06 × 10^−6^
Cerebral malaria	40% (1356/1953/637)	37% (458/590/141)	A	0·83 (0·75–0·91)	0·0001	0·83 (0·76–0·92)	0·0001
Severe malarial anaemia	40% (1356/1953/637)	34% (275/299/65)	A	0·72 (0·64–0·82)	1·55 × 10^−6^	0·72 (0·65–0·84)	5·45 × 10^−6^
Respiratory distress	40% (1356/1953/637)	35% (272/311/79)	A	0·78 (0·69–0·89)	0·0001	0·79 (0·70–0·90)	0·0002
Death	40% (1356/1953/637)	33% (108/117/24)	A	0·70 (0·57–0·85)	0·0003	0·72 (0·59–0·88)	0·0004

When more than one SNP was genotyped at a locus, we show ORs for the SNP with the lowest p value for severe malaria or any one of its phenotypes. Individuals with multiple phenotypes are included in each of those phenotype groups for analysis. het=heterozygous. hom=homozygous. OR=odds ratio. SNP=single nucleotide polymorphism.

* Statistical models were additive (A), heterozygous (H), or recessive (R).

† Adjusted for ethnic group, gender, and rs334.

‡ The recessive model for rs8176719 is presented and represents blood group O.

§ The derived allele of rs8176746 identifies blood group B.

**Table 3:** X-chromosome SNPs with significant signals of association in different severe malaria phenotypes

	**Frequency (%) of derived allele in controls (normal/het/hom [n])**	**Frequency (%) of derived allele in cases (normal/het/hom [n])**	Model*	**Unadjusted**		**Adjusted**†	
				OR (95% CI)	p value	OR (95% CI)	p value
***CD40LG* (rs3092945; T→C)**							
Severe malaria (male and female)	21% (2760/646/521)	26% (1459/388/379)	A	1·14 (1·06–1·22)	0·0002	1·15 (1·06–1·23)	0·0002
Cerebral malaria (male and female)	21% (2760/646/521)	25% (815/199/209)	A	1·32 (1·10–1·59)	0·003	1·37 (1·13–1·67)	0·003
Severe malarial anaemia (male and female)	21% (2760/646/521)	26% (442/121/120)	A	1·17 (1·05–1·31)	0·0001	1·18 (1·05–1·33)	0·003
Respiratory distress (male and female)	21% (2760/646/521)	25% (454/128/98)	A	1·28 (1·01–1·63)	0·04	1·27 (1·09–1·64)	0·04
Death (male and female)	21% (2760/646/521)	25% (454/128/98)	A	1·30 (0·88–1·92)	0·18	1·32 (0·88–2·00)	0·15
***CD40LG* (rs3092945; T→C)**							
Severe malaria (female)	21% (1215/646/81)	25% (614/388/80)	A	1·25 (1·10–1·42)	0·0004	1·28 (1·13–1·45)	0·0002
Cerebral malaria (female)	21% (1215/646/81)	26% (355/199/48)	A	1·89 (1·30–2·76)	0·001	1·23 (1·05–1·43)	0·007
Severe malarial anaemia (female)	21% (1215/646/81)	25% (181/121/26)	A	1·33 (1·10–1·62)	0·003	1·36 (1·12–1·64)	0·001
Respiratory distress (female)	21% (1215/646/81)	24% (182/128/15)	A	1·29 (1·01–1·63)	0·03	1·31 (1·03–1·66)	0·02
Death (female)	21% (1215/646/81)	24% (182/128/15)	A	1·30 (0·88–1·92)	0·18	1·32 (0·90–1·95)	0·15
***CD40LG* (rs3092945; T→C)**							
Severe malaria (male)	78% (440/0/1545)	73% (299/0/845)	HM	0·83 (0·70–0·99)	0·03	0·80 (0·68–0·95)	0·01
Cerebral malaria (male)	78% (440/0/1545)	76% (161/0/460)	HM	0·84 (0·68–1·04)	0·11	0·81 (0·66–1·00)	0·05
Severe malarial anaemia (male)	78% (440/0/1545)	73% (94/0/261)	HM	0·81 (0·63–1·05)	0·12	0·79 (0·61–1·02)	0·07
Respiratory distress (male)	78% (440/0/1545)	75% (83/0/272)	HM	0·95 (0·72–1·23)	0·71	0·93 (0·71–1·21)	0·61
Death (male)	78% (440/0/1545)	75% (83/0/272)	HM	0·93 (0·62–1·40)	0·75	0·91 (0·61–1·36)	0·65
***G6PD* (rs1050828; C→T)**							
Severe malaria (male and female)	19% (2863/639/438)	19% (1643/306/271)	H	0·82 (0·70–0·97)	0·01	0·80 (0·68–0·96)	0·01
Cerebral malaria (male and female)	19% (2863/639/438)	18% (921/168/131)	H	0·80 (0·65–0·98)	0·02	0·79 (0·64–0·97)	0·02
Severe malarial anaemia (male and female)	19% (2863/639/438)	23% (479/90/111)	R	1·60 (1·26–2·03)	0·0001	1·72 (1·33–2·22)	0·0001
Respiratory distress (male and female)	19% (2863/639/438)	18% (510/98/72)	H	0·90 (0·74–1·09)	0·28	0·91 (0·74–1·11)	0·28
Death (male and female)	19% (2863/639/438)	18% (510/98/72)	H	0·64 (0·46–0·89)	0·006	0·64 (0·45–0·90)	0·006
***G6PD* (rs1050828; C→T)**							
Severe malaria (female)	19% (1250/639/62)	18% (729/306/39)	H	0·82 (0·70–0·97)	0·01	0·81 (0·69–0·96)	0·01
Cerebral malaria (female)	19% (1250/639/62)	18% (410/168/122)	H	0·80 (0·65–0·98)	0·03	0·79 (0·65–0·97)	0·02
Severe malarial anaemia (female)	19% (1250/639/62)	17% (224/90/11)	H	0·79 (0·61–1·03)	0·07	0·78 (0·60–1·02)	0·06
Respiratory distress (female)	19% (1250/639/62)	19% (215/198/12)	H	0·89 (0·58–1·38)	0·61	0·88 (0·68–1·14)	0·35
Death (female)	19% (1250/639/62)	19% (215/198/12)	H	0·56 (0·37–0·85)	0·004	0·57 (0·38–0·86)	0·005
***G6PD* (rs1050828; C→T)**							
Severe malaria (male)	18% (1613/0/376)	20% (914/0/232)	HM	1·11 (0·92–1·33)	0·26	1·08 (0·90–1·30)	0·36
Cerebral malaria (male)	18% (1613/0/376)	18% (511/0/109)	HM	0·92 (0·72–1·17)	0·48	0·91 (0·72–1·15)	0·45
Severe malarial anaemia (male)	18% (1613/0/376)	28% (255/0/100)	HM	1·72 (1·32–2·23)	6·98 × 10^−5^	1·68 (1·30–2·17)	0·0001
Respiratory distress (male)	18% (1613/0/376)	17% (295/0/60)	HM	0·89 (0·66–1·21)	0·89	0·87 (0·64–1·17)	0·36
Death (male)	18% (1613/0/376)	17% (295/0/60)	HM	0·77 (0·48–1·24)	0·28	0·77 (0·48–1·23)	0·26

Data are from models that were most significant. Individuals with multiple phenotypes are included in each of those phenotype groups for analysis. Analyses were run on males and females separately and combined, using standard meta-analysis techniques. het=heterozygous. hom=homozygous. OR=odds ratio. SNP=single nucleotide polymorphism.

* Statistical models were additive (A), heterozygous (H), hemizygous male (HM), or recessive (R).

† Adjusted for ethnic group, gender, and rs334 genotype.

As anticipated, the strongest association with risk of severe malaria was with rs334 in *HBB*, which leads to the sickle-cell trait (adjusted OR 0·15, 95% CI 0·11–0·20; p=2·61 × 10^−58^), with similar effect sizes for all three major severe malaria phenotypes (table 2; appendix pp 7–16). Strong associations with risk of severe malaria were also seen for polymorphisms in *ABO* and *HBA*, including rs8176719 in *ABO* (representing blood group O; adjusted OR 0·74, 95% CI 0·66–0·72; p=6·26 × 10^−8^), rs8176746 in *ABO* (identifying blood group B; 1·38, 95% CI 1·22–1·50; p=2·38 × 10^−7^), and a 3·7 kb deletion in *HBA* (−α^3·7^-thalassaemia; 0·83, 0·76–0·90; p=2·06 × 10^−6^).

Further polymorphisms associated with severe malaria were noted for rs1541255 in *ATP2B4* (adjusted OR 0·76, 95% CI 0·63–0·92; p=0·001) and rs186873296 located in an intergenic region between *FREM3* and *GYPE* (0·64, 0·53–0·79; p=3·18 × 10^−14^). rs186873296 between *FREM3* and *GYPE* was associated with similar levels of protection against all three major phenotypes of severe malaria and protected against inpatient mortality (table 2). The effect of rs186873296 was additive for severe malaria overall (heterozygosity, adjusted OR 0·57, 95% CI 0·49–0·68; p=3·22 × 10^−11^; homozygosity, 0·26, 0·11–0·62; p=0·002; appendix p 17). The rs186873296 *FREM3* polymorphism also correlated closely with the polymorphism that is specific to the Dantu blood group antigen (concordance 1907 of 1938 [98%]; appendix p 28).

Because some individuals with more than one phenotype for severe malaria were included in more than one analysis group, analyses were also done in which individuals were only included in single analytical groups (appendix pp 18–23, 30). On visual inspection and comparison, these analyses were largely concordant with our overall analyses. To exclude the possibility of epistasis between individual malaria-associated genes, which holds the potential for concealing true associations in overall analyses, pairwise analyses were done to test for gene–gene interactions between the most significant polymorphisms (appendix pp 24, 31). The smallest p value we found (p=0·02) was between polymorphisms in *HBB* (rs334) and *HBA* loci, which result in sickle-cell trait or sickle-cell disease and in –α^3·7^-thalassaemia, respectively. This analysis showed evidence of a negative interaction between sickle-cell trait and –α^3·7^-thalassaemia through which the protective effects of each polymorphism inherited independently seemed to be lost when both were inherited together (appendix pp 24, 32).

The effect of polymorphisms associated with risk for severe malaria on other variables of severe malaria—including parasite densities (the number of parasites in circulation, assessed by visually counting parasites on peripheral blood films) or haematological indices—was also investigated. Three significant associations were noted between candidate polymorphisms and malaria parasite densities (table 4). Parasitaemia was significantly lower among cases with severe malaria and sickle-cell trait compared with those with severe malaria and normal haemoglobin (p=3·66 × 10^−6^), among homozygotes for the derived allele (GG) at rs1541255 of *ATP2B4* compared with homozygotes for the ancestral allele (AA; p=0·04), and among children with blood group O compared with non-blood group O cases (p=0·02). Significant associations were also noted between polymorphisms and haematological indices (appendix p 25). Haemoglobin concentrations and red-blood-cell counts were significantly higher in children with sickle-cell trait and significantly lower in those with sickle-cell disease compared with children with normal haemoglobin, whereas haemoglobin concentrations were higher in children with the rs186873296 SNP in *FREM3* homozygous for the derived allele (GG) compared with those homozygous for the ancestral allele (AA; appendix p 25).

**Table 4:** *Plasmodium falciparum* parasite densities in patients with severe malaria and loci included in the univariate analysis

	**Patients (n)**	**Parasite density per μL**	p value*
***ATP2B4* (rs1541255)**			
Normal (AA)	989	43 540 (37 370–50 729)	..
Heterozygous (AG)	1005	40 027 (34 396–46 579)	0·72
Homozygous (GG)	181	28 635 (20 033–40 929)	0·04
***IL10* (rs1800890)**			
Normal (AA)	1294	39 224 (34 329–44 817)	..
Heterozygous (AT)	808	42 156 (35 612–49 902)	0·78
Homozygous (TT)	95	46 900 (28 675–76 707)	0·77
***LPHN2* (also known as *ADGRL2*; rs72933304)**			
Normal (CC)	1880	40 179 (35 962–44 890)	..
Heterozygous (AC)	292	42 316 (31 939–56 066)	0·93
Homozygous (AA)	8	36 451 (6660–199 493)	0·99
***LOC727982* (rs1371478)**			
Normal (CC)	1065	40 204 (34 692–46 591)	..
Heterozygous (CT)	946	41 676 (35 640–48 734)	0·94
Homozygous (TT)	162	34 272 (23 482–50 019)	0·72
***ARL14* (rs75731597)**			
Normal (AA)	1726	41 555 (37 009–46 660)	..
Heterozygous (AC)	394	34 628 (27 171–44 130)	0·37
Homozygous (CC)	18	34 337 (11 042–106 776)	0·94
***FREM3* (rs186873296)**			
Normal (AA)	1933	41 607 (37 305–46 405)	..
Heterozygous (AG)	232	32 556 (23 759–44 611)	0·31
Homozygous (GG)	5	119 644 (13 995–1 022 828)	0·59
***INPP4B* (rs77389579)**			
Normal (GG)	1985	39 666 (35 611–44 182)	..
Heterozygous (GT)	186	48 607 (34 175–69 133)	0·52
Homozygous (TT)	2	293 153 (9811–8 759 196)	0·48
***ABO* (rs8176719)**†			
Non-blood group O	1161	46 170 (40 068–54 455)	..
Blood group O	1026	35 092 (30 220–40 749)	0·02
***ABO* (rs8176746)**‡			
Normal (CC)	1504	38 608 (34 118–43 688)	..
Heterozygous (AC)	613	44 829 (36 937–54 406)	0·40
Homozygous (AA)	74	46 817 (26 814–81 739)	0·78
***HBB* (rs334)**			
Normal (AA)	2125	42 857 (38 648–47 525)	..
Heterozygous (AT)	56	8649 (4575–16 350)	3·66 × 10^−6^
Homozygous (TT)	11	8850 (2103–37 235)	0·08
***RPS6KL1* (rs3742785)**			
Normal (CC)	1015	42 920 (36 915–44 901)	..
Heterozygous (AC)	881	39 668 (33 743–46 633)	0·76
Homozygous (AA)	185	44 458 (31 235–63 279)	0·98
***HBA* (α^−3·7^-thalassaemia)**			
Normal	843	39 969 (33 877–47 157)	..
Heterozygous	1009	40 292 (34 639–46 867)	0·99
Homozygous	257	38 243 (28 345–51 598)	0·96
***CD40LG* (rs3092945)**			
Normal (TT)	1432	39 307 (34 627–44 619)	..
Heterozygous (CT)	383	44 242 (34 624–56 532)	0·67
Homozygous (CC)	375	42 007 (32 789–53 815)	0·88
***G6PD* (rs1050828)**			
Normal (CC)	1616	41 464 (36 808–46 708)	..
Heterozygous (CT)	301	41 157 (31 232–54 236)	0·99
Homozygous (TT)	267	35 063 (26 158–47 000)	0·55

Data are geometric mean (95% CI).

* Adjusted for ethnicity, gender and rs334 genotype; for this subanalysis of parasite densities, we judged p<0·05 significant.

† The rs8176719 polymorphism identifies blood group O in the homozygous deletion form.

‡ The derived allele of rs8176746 identifies blood group B.

The proportion of variance in the risk of severe malaria that could be accounted for by genetic polymorphisms and other factors was estimated, and 5·2% of the total variance in the overall risk of severe malaria was accounted for by polymorphisms in 12 genes: *HBB, FREM3, ABO, HBA* (*HBA1* and *HBA2*), *ATP2B4*, LOC727982, *ARL14, IL10, RPS6KL1, LPHN2* (*ADGRL2*), *INPP4B*, and *EMR1* (appendix p 34). Inheritance of sickle-cell trait at *HBB* (rs334) accounted alone for 2·9% of the total variance, *FREM3* (rs186873296) for 0·6%, and the remaining ten genes had a combined total of 1·7% (appendix pp 26, 27). Ethnic origin contributed to 1·9% of the total variance in the risk of contracting severe malaria and accounted for less than 10% of the variance from any of the individual genes. Gender contributed only 0·009% of the total variance in the risk of severe malaria. Thus, 93% of the variance in the overall risk observed could not be accounted for in our model. The inheritance of polymorphisms in *FREM3* and *INPP4B* were in high linkage disequilibrium (appendix p 33); each constituted a proportion of the variance attributable to the other, accounting for the lower variances for these polymorphisms individually when combined in one model (appendix pp 26, 27).

## Discussion

We found significant associations between risk for severe malaria and polymorphisms in 15 genes. Although data for some of these genes have been reported previously,^11^ our analysis includes new candidates that have been identified from subsequent genome-wide association studies^9^, ^12^ and provides additional data about the relations between specific polymorphisms and a range of clinical and laboratory phenotypes among severe malaria cases (eg, malaria parasite densities and haematological indices). Of note, most of the significant associations were linked to genes that have a role in red-blood-cell phenotype or function, the haematological outcomes for some of which remain largely undocumented.

In keeping with the epidemiological setting of our study site, within an area of relatively low *P falciparum* transmission, cerebral malaria accounted for the largest proportion of severe malaria cases and was associated with the highest mortality.^18^ However, we also recorded considerable overlap in the phenotypic presentation of severe malaria. This observation, which accords with those of previous studies,^19^ made it difficult for us to detect syndrome-specific effects, and is a weakness of our case-control approach. Moreover, we confirmed the strong protective advantage against severe malaria of several well established candidate polymorphisms, including inherited red-blood-cell characteristics resulting in sickle-cell trait, G6PD deficiency, and blood group O. The relations between these polymorphisms and various forms of clinical malaria have been discussed previously.^20^ Although we also confirmed the long-recognised protective genetic advantage of –α^3·7^-thalassaemia against severe malaria, by contrast with previous reports,^21^ we noted that protection was not restricted to severe malaria anaemia but was seen equally against all other phenotypes of severe malaria. This finding is most likely accounted for by the limited statistical power of earlier reports,^21^ emphasising the need for caution when interpreting the results of smaller studies.

Our study confirms the relevance of associations between severe malaria and several genes that have been described previously. *ATP2B4* on chromosome 1 encodes for PMCA4, the main red-blood-cell calcium (Ca^2+^) pump.^22^ On the basis of studies conducted in vitro, variant forms of *ATP2B4* have been shown to affect platelet and endothelial function.^23^ Other genes of interest relate to the glycophorin molecules, which have long been recognised as receptors for red-blood-cell invasion by *P falciparum* merozoites.^24^ In the case of GYPA, red-blood-cell invasion is mediated through its specific interaction with Duffy binding like (DBL) domains on the *P falciparum* merozoite surface protein EBA-175,^25^ but some strains of *P falciparum* parasites can also invade through pathways that involve interactions between other glycophorin molecules and a range of alternative merozoite ligands.^26^ Mutant forms of these glycophorin molecules could, therefore, be attractive targets for drug and vaccine development.

A significant association signal of *ATP2B4* was first identified in a genome-wide association case-control study of severe malaria undertaken in Ghana, and replicated in subsequent severe malaria case-control studies.^20^ However, to the best of our knowledge, the haematological and wider health outcomes of this polymorphism, and its association with risk for specific phenotypes of severe malaria, were not known previously. The polymorphism rs1541255 in *ATP2B4* was common in our study population, with an allele frequency of 33% among controls, consistent with selection through a survival advantage against malaria. Association signals detected within *ATP2B4* in case-control studies of severe malaria undertaken to date might reflect their linkage disequilibrium with a functional mutation in the promoter region of *ATP2B4* that results in reduced expression of red-blood-cell PMCA4 and, as a result, in reduced red-blood-cell Ca^2+^ extrusion.^27^ In view of the central role that Ca^2+^ has in the physiology of *P falciparum* red-blood-cell invasion,^28^ it is noteworthy that homozygosity at rs1541255 was one of the few genotypes to be associated with reduced parasite densities in our study. Ca^2+^ is also important in both clumping of *P falciparum*-infected red blood cells and in their sequestration to the vascular endothelium, both of which could lead to severe and complicated malaria through the occlusion of blood vessels in essential tissues, including the brain.^29^ As such, several plausible explanations could account for how mutations in *ATP2B4* might be relevant to protection against severe malaria that could potentially be exploited for development of therapeutic agents. Further studies are being done to confirm the precise mutation within *ATP2B4* that is associated with malaria protection and describing its effects both on tissue-specific PMCA4 structure and function and on malaria-specific pathophysiological processes.

A second association, previously identified in a genome-wide association study of severe malaria that included a subset of data and samples in our study,^9^, ^12^ was seen at SNP rs186873296 in *FREM3*. The rs186873296 association tags a haplotype for the neighbouring region comprising *GYPA, GYPB*, and *GYPE*^9^ and, in particular, a hybrid gene comprising *GYPA* and *GYPB,* which encodes a blood antigen known as Dantu.^12^ Instead of expressing normal GYPA and GYPB molecules, people with Dantu produce a hybrid molecule that consists of the extracellular domain of GYPB and the transmembrane plus intracellular domains of GYPA.^30^ In our extended sample set of severe malaria cases from Kenya, we found that the rs186873296 *FREM3* polymorphism correlated closely with the polymorphism that is specific to Dantu.^12^ A second association signal, at *INPP4B* within the same gene region, seemed to also account for its close chromosomal proximity and strong linkage disequilibrium with Dantu. This finding supports the conclusion that the statistical association between severe malaria risk and SNPs in both *FREM3*—a gene of unknown biological relevance—and *INPP4B* are not in themselves causal with respect to malaria susceptibility but rather their associations might be accounted for by their close linkage disequilibrium with Dantu, which could most plausibly provide a malaria-protective mechanism on the basis of biological theory. Nevertheless, by contrast with sickle-cell trait, *ATP2B4*, and blood group O, we saw no discernible effect of rs186873296 on parasite densities in severe malaria cases within our study.

On the one hand, although the Dantu polymorphism does not seem to offer protection by reducing red-blood-cell parasite invasion, the absence of any apparent effect on parasite densities might simply show that patients have severe malaria, and does not exclude the possibility that the malaria-protective effect of Dantu might be restricted to red-blood-cell invasion via a glycophorin-specific pathway by a subset of *P falciparum* parasites. On the other hand, the mechanism by which Dantu protects against severe malaria might not be mediated by reduced parasite invasion at all, but instead relate to the pathophysiology of malaria more broadly. Further work will be needed to elucidate the mechanisms definitively.

Comparative genetic analysis of chimpanzees and human beings has shown that the *GYPA, GYPB*, and *GYPE* loci lie in an area of the genome that has characteristics of ancient balancing selection.^12^ Thus, Dantu might also be under balancing selection, in which its positive selection by malarial disease might be balanced by negative selection from a biological disadvantage yet to be discovered. As such, it will also be important to ascertain whether homozygosity for Dantu is associated with any negative outcomes.

The associations between sickle-cell trait and homozygosity for rs186873296 in *FREM3* and haematological indices among severe malaria cases are noteworthy. The most likely reason for the high haemoglobin concentrations in cases with both these genotypes is that such children are protected from all forms of clinical malaria that result cumulatively in higher levels of anaemia in children without these protective polymorphisms. Although this protection is well recognised for sickle-cell trait,^21^ further studies will be needed to show that this is also true for *FREM3*. Moreover, the lower mean red-blood-cell volume and higher red-blood-cell count that we observed in sickle-cell trait cases is consistent with the findings from a previous study conducted in the same population.^31^

How the remaining polymorphisms described both here and in previous studies can be exploited for therapeutic advantage, in terms of their associations with the overall risk of severe malaria, remains unclear. For example, in agreement with previous studies,^32^, ^33^ we found that polymorphisms in *CD40LG* were associated with a significantly increased risk of severe malaria, but by contrast with findings of a case-control study of severe childhood malaria in Tanzania,^20^ we found no evidence that this association was specific to a phenotype of severe malaria. The absence of an association between *CD40LG* and a specific phenotype of severe malaria in our current study presumably reflects the greater power of our study relative to earlier studies, because small studies are prone to false-positive findings as a result of statistical chance. Likewise, although significant associations were seen between severe malaria and polymorphisms in seven additional genes (*IL10, LPHN2* [*ADGRL2*], LOC727982, *ARL14, RPS6KL1, CAND1*, and *GNAS*), without further data their potential for translation remains elusive.

Despite strong associations between severe malaria and several candidate polymorphisms, our analyses suggest that together they only accounted for a small proportion (5·2%) of the total variance in risk for contracting severe malaria in Kilifi County overall, only a quarter of the overall heritability attributed in an earlier study.^3^ Perhaps additional genetic associations remain to be discovered, our modelling approach was inadequate, or the proportion of variance that we attributed to heritability in our previous study was overestimated by the verbally reported pedigree-based method that we used,^3^ because of confounding between families and household-related environmental factors that were not captured completely by fitting both these terms in our model. The low variance attributable to genes with strong individual protective effects might be caused partly by the low population frequencies of some of these alleles. For example, the rs334 mutation in *HBB* contributes only 2·9% to the total variance in risk for severe malaria despite its large effect size because protection is only afforded to a small proportion (about 15%) of the population.

In a previous study,^32^ a higher proportion of variance was accounted for by both *HBB* (6·3%) and other polymorphisms compared with the proportions we reported in our study. However, the estimates in that previous study were not adjusted for the ascertainment bias that will have resulted from the study's case-control design. It is also not clear whether the earlier estimates were generated on the observed or underlying liability scale, resulting in bias from the overall prevalence. These failures to account for prevalence and study design in previous studies highlight the need to apply the adjustments we made to compare heritability estimates across populations validly.

In conclusion, in accordance with findings of previous studies, we report that the strongest protective genetic associations against severe malaria all relate consistently to polymorphisms that affect the structure or function of red blood cells. As a result, some polymorphisms have been selected to very high levels. For example, almost two-thirds of the population in our study area were affected by –α^3·7^-thalassaemia, and large proportions of the population are affected by other polymorphisms, many of which have unknown haematological implications. Moreover, because such polymorphisms are so common, many children inherit more than one, with outcomes that—in most cases—are even less well understood. It will be important to better understand the mechanisms by which these red-blood-cell conditions protect against severe malaria, along with their broader haematological outcomes. Future research should determine how malaria-protective mechanisms might be emulated to uncover potential new approaches to the prevention and treatment of severe malaria.
