# Supplementary material for: Human candidate gene polymorphisms and risk of severe malaria in children in Kilifi, Kenya: a case-control association study
Source: Lancet Haematol. 2018 Jul 20;5(8):e333–45. doi: 10.1016/S2352-3026(18)30107-8 (PMC6069675; doi:10.1016/S2352-3026(18)30107-8)
Supplement: Supplementary appendix [file mmc1.pdf]

THE LANCET
Haematology

Supplementary appendix

This appendix formed part of the original submission and has been peer reviewed. We post it as supplied by the authors.

Supplement to: Ndila CM, Uyoga S, Macharia AW, et al, and the MalariaGEN Consortium.Human candidate gene polymorphisms and risk of severe malaria in children in Kilifi, Kenya: a case-control association study. *Lancet Haematol* 2018; published online July 19. http://dx.doi.org/10.1016/S2352-3026(18)30107.

Supplementary Material for Ndila *et al.*

Human candidate gene polymorphisms and risk of severe malaria in children in Kilifi, Kenya: a case-control association study

**Table S1: Summary of genes and genetic loci included in the study.**	**2-4**
**Table S2: Assay definitions for the Agena iPLEX genotyping platform.**	**5-6**
**Table S3: Odds ratios for all SM for all polymorphisms investigated.**	**7-8**
**Table S4: Odds ratios for all CM for all polymorphisms investigated.**	**9-10**
**Table S5: Odds ratios for all SMA for all polymorphisms investigated.**	**11-12**
**Table S6: Odds ratios for all RD for all polymorphisms investigated.**	**13-14**
**Table S7: Odds ratios for other-SM for all polymorphisms investigated.**	**15-16**
**Table S8: Tests of association by specific genotypes for the most significant loci in the case-control study.**	**17**
**Table S9: Odds ratios for CM only for all polymorphisms investigated.**	**18-19**
**Table S10: Odds ratios for SMA only for all polymorphisms investigated.**	**20-21**
**Table S11: Odds ratios for RD only for all polymorphisms investigated.**	**22-23**
**Table S12: Pairwise interactions between the polymorphisms (1 per gene) at autosomal loci at which we found significant associations in our primary analyses using the likelihood ratio approach (Main Text Table 2).**	**24**
**Table S13: Association of Red Blood Cell indices for all severe malaria cases.**	**25**
**Table S14: Proportions of variance explained by individual markers.**	**26-27**
**Table S15: Concordance between iPLEX assay for rs186873296 and the BlpI RFLP assay for the Dantu hybrid in paediatric cases of severe malaria**	**28**
**Figure S1: Flow diagram showing the selection of severe malaria cases.**	**29**
**Figure S2: Manhattan plot showing the distribution of p-values for association tests with severe malaria.**	**30**
**Figure S3: Network diagram for interaction of polymorphim pairs in severe malaria.**	**31**
**Figure S4: Two-way epistatic interactions between rs334 (HbS) and $\alpha^{-3.7}$-thalassaemia for all severe malaria cases.**	**32**
**Figure S5: Linkage disequilibrium among the 13 polymorphims explaining most of the variance in the population**.	**33**
**Figure S6: Proportion of variance in the risk of severe malaria among children that is explained by genetic polymorphisms and other factors.**	**34**
**Appendix 1: MalariaGEN Consortium members**	**35-39**

#	Gene Symbol	Gene Description	SNP ID	Chr	Position	Removed in QC
**1**	*AJAP1*	Adherens junctions associated protein 1	rs113788643	1	4817664	
			rs6674631	1	4834821	
**2**	*LPHN2*	Latrophilin 2	rs146428334	1	81688714	
			rs72933304	1	81726138	
			rs72933310	1	81727427	
			rs72933350	1	81751439	
			rs4650365	1	81770827	
**3**	*GBP7*	Guanylate binding protein 7	rs1803632	1	89582690	
**4**	*DARC*	Duffy blood group, atypical chemokine receptor	rs2814778	1	159174683	
**5**	*ATP2B4*	ATPase, Ca^{2+} transporting, plasma membrane 4	rs55868763	1	203652140	
			rs1541255	1	203652141	
			rs10900585	1	203654024	
			rs4951074	1	203660781	
			rs3753036	1	203677250	
**6**	*IL10*	Interleukin 10	rs3024500	1	206940831	
			rs1800896	1	206946897	
			rs1800890	1	206949365	
**7**	*CR1*	Complement receptor 1	rs17047660	1	207782856	
			rs17047661	1	207782889	
**8**	*LOC727982*	Long intergenic non-protein coding RNA 1249	rs1371478	2	4901589	
			rs1371474	2	4909777	
			rs10188961	2	4926593	
**9**	*LAPTM4A*	Lysosomal protein transmembrane 4 alpha	rs973128	2	20332487	
**10**	*SDC1*	Syndecan 1	rs11899121	2	20367973	
**11**	*IL1A*	Interleukin 1, alpha	rs17561	2	113537223	
**12**	*IL1B*	Interleukin 1, beta	rs1143634	2	113590390	
**13**	*ZSWIM2*	Zinc finger, SWIM-type containing 2	rs4316902	2	188007364	
			rs144778284	2	188012821	
**14**	*IL17RE*	Interleukin 17 receptor E	rs708567	3	9960070	
**15**	*OXNAD1*	Oxidoreductase NAD-binding domain containing 1	rs200704287	3	16407519	monomorphic
			rs79691057	3	16408251	monomorphic
			rs75180423	3	16408723	monomorphic
**16**	*TLR9*	Toll-like receptor 9	rs187084	3	52261031	
**17**	*IL17RD*	Interleukin 17 receptor D	rs6780995	3	57138419	
**18**	*ARL14*	ADP-ribosylation factor-like 14	rs76033371	3	160362359	
			rs75731597	3	160364808	
			rs74954675	3	160381509	
**19**	*B3GALNT1*	Beta-1,3-N-acetylgalactosaminyltransferase 1 (Globoside blood group)	rs12107243	3	160793678	
**20**	*TLR1*	Toll-like receptor 1	rs4833095	4	38799710	
**21**	*TLR6*	Toll-like receptor 6	rs5743810	4	38830350	
			rs5743809	4	38830514	
**22**	*INPP4B*	Inositol polyphosphate-4-phosphatase, type II, 105kda	rs77389579	4	143538511	
			rs13103597	4	143558581	
**23**	*USP38*	Ubiquitin specific peptidase 38	rs4266246	4	143971242	
			rs28459062	4	144039139	
**24**	*GAB1*	GRB2-associated binding protein 1	rs7663712	4	144261117	
**25**	*GUSBP5*	Glucuronidase, beta pseudogene 5	rs148111931	4	144540045	monomorphic
**26**	*FREM3*	FRAS1 related extracellular matrix 3	rs184908374	4	144665753	
			rs149914432	4	144666678	
			rs186790584	4	144680140	
			rs184895969	4	144698528	
			rs186873296	4	144702474	
**27**	*C6*	Complement component 6	rs1801033	5	41199959	
**28**	*IRF1*	Interferon regulatory factor 1	rs2706384	5	131826880	
**29**	*IL13*	Interleukin 13	rs20541	5	131995964	
**30**	*IL4*	Interleukin 4	rs2243250	5	132009154	
**31**	*GABBR1*	Gamma-aminobutyric acid (GABA) B receptor, 1	rs192151845	6	29588309	

**32**	*HCG4*	HLA complex group 4	rs114980857	6	29772098	
**33**	*LTA*	Lymphotoxin alpha	rs2239704	6	31540141	
			rs909253	6	31540313	
**34**	*TNF*	Tumour necrosis factor	rs1799964	6	31542308	
			rs1800750	6	31542963	
			rs1800629	6	31543031	
			rs361525	6	31543101	
			rs3093662	6	31544189	
**35**	*HSPA1B*	Heat shock protein family A (HSP70) member 1B	rs6457452	6	31795550	
**36**	*SNORD48*	Small nucleolar RNA, C/D box 48	rs116288147	6	31803074	
**37**	*CTL4*	SLC44A4 (Solid carrier family 44, member 4)	rs2242665	6	31839309	
**38**	*IL20RA*	Interleukin 20 receptor, alpha	rs1555498	6	137325847	
**39**	*PLEKHG1*	Pleckstrin homology domain containing, family G member 1	rs55958968	6	150942218	
			rs144224092	6	150973623	missingness
			rs79100774	6	150975934	
			rs114726617	6	150980481	
			rs2131263	6	150981102	
			rs76924464	6	150982529	
			rs151293197	6	150994429	
			rs142712208	6	151026346	
			rs15116938	6	151046029	
			rs141555199	6	151048708	
**40**	*NOD1*	Nucleotide-binding oligomerization domain containing 1	rs2075820	7	30492237	
**41**	*CD36*	CD36 molecule (thrombospondin receptor)	rs3211938	7	80300449	
**42**	*TLR4*	Toll-like receptor 4	rs4986790	9	120475302	
			rs4986791	9	120475602	
**43**	*ABO*	ABO blood group	rs8176750	9	136131057	
			rs56390333	9	136131064	
			rs8176746	9	136131322	
			rs8176719	9	136132909	
**44**	*MKI67*	Marker of proliferation Ki-67	rs11016116	10	129975450	
			rs148494166	10	129976030	
			rs115947774	10	130072795	
**45**	*RHOG*	Ras homolog family member G	rs138826089	11	3847190	monomorphic
**46**	*RRM1*	Ribonucleotide reductase M1	rs116472045	11	4111415	
**47**	*HBB*	Haemoglobin beta chain	rs334	11	5248232	
**48**	*TRIM5*	Tripartite motif containing 5	rs7935564	11	5718517	
**49**	*RTN3*	Reticulon 3	rs542998	11	63487386	
**50**	*GRIP1*	Glutamate receptor interacting protein 1	rs192909543	12	67366471	monomorphic
			rs1394263	12	67366537	
**51**	*CAND1*	Cullin-associated and neddylation-dissociated 1	rs1566830	12	67369898	
			rs12307123	12	67394950	
			rs10459266	12	67455888	
**52**	*IL22*	Interleukin 22	rs2227507	12	68642647	
			rs1012356	12	68644618	
			rs2227491	12	68646521	
			rs2227485	12	68647713	
			rs2227478	12	68648622	
**53**	*TPTE2*	Transmembrane phosphoinositide 3-phosphatase and tensin homolog 2	rs182873742	13	20050239	
**54**	*SPTB*	Spectrin, beta, erythrocytic	rs229587	14	65263300	
**55**	*LTBP2*	Latent transforming growth factor beta binding protein 2	rs74063230	14	75066093	monomorphic
**56**	*YLPM1*	YLP motif containing 1	rs10139016	14	75274288	
**57**	*RPS6KL1*	Ribosomal protein S6 kinase-like 1	rs3742785	14	75373034	
**58**	*ADCY9*	Adenylate cyclase 9	rs2230739	16	4033436	
			rs10775349	16	4079823	
**59**	*HBA*	Haemoglobin, alpha	α^{+}thalassaemia	16	222846	
**60**	*IL4R*	Interleukin 4 receptor	rs1805015	16	27374180	
**61**	*ADORA2B*	Adenosine A2b receptor	rs2535611	17	15861332	
**62**	*NOS2*	Nitric oxide synthase 2A (inducible, hepatocytes)	rs2297518	17	26096597	
			rs1800482	17	26128509	

			rs9282799	17	26128728	
			rs8078340	17	26129212	
**63**	*BCAS3*	Microtubule associated cell migration factor	rs184142841	17	58855323	monomorphic
**64**	*TBX2*	T-box 2	rs73991577	17	59323072	
**65**	*EMR1*	ADGRE1 (Adhesion G-protein-coupled receptor E1)	rs373533	19	6919624	
			rs461645	19	6919753	
**66**	*ICAM*	Intercellular adhesion molecule 1	rs5498	19	10395683	
**67**	*GNAS*	Guanine nucleotide binding protein, alpha	rs8386	20	57485812	
**68**	*DERL3*	Derlin 3	rs1128127	22	24179132	
**60**	*CD40LG*	CD40 ligand (TNF superfamily, member 5)	rs3092945	X	135729609	
			rs1126535	X	135730555	
**70**	*G6PD*	Glucose-6-phosphate dehydrogenase	rs1050829	X	153763492	
			rs1050828	X	153764217	

Chr = chromosome, Removed in QC = SNPs that failed QC and were removed from further analysis. Co-ordinate system is with respect to GRCh37.

Table S1: Summary of genes and genetic loci included in the study.

well	term	snp_id	2nd-pcrp	1st-pcrp	amp_len	up_conf	mp_conf	Tm(NN)	pcgc	pwarn	uep_dir	uep_mass	uep_seq	ext1_call	ext1_mass	ext1_seq	ext2_call	ext2_mass	ext2_seq	ext3_call	ext3_mass	ext3_seq	ext4_call	ext4_mass	ext4_seq
W1	iPLEX	rs2227478	ACGTTGGATGTGAGATGGCACAGACCTAAG	ACGTTGGATGTCTGGCCACCTTCACAAATG	104	99.9	70.5	48	60		R	4534.9	GTGGTTCCGACCCTT	G	4782.1	GTGGTTCCGACCCTTC	A	4862	GTGGTTCCGACCCTTT						
W1	iPLEX	rs361525	ACGTTGGATGCAGGGTCCTACACACAAATC	ACGTTGGATGAAGCATCAAGGATACCCCTC	115	99.1	70.3	52	68.8	d	R	4689	CCCATCCTCCCTGCTC	G	4936.2	CCCATCCTCCCTGCTCC	A	5016.1	CCCATCCTCCCTGCTCT						
W1	iPLEX	rs5498	ACGTTGGATGTGTCACTCGAGATCTTGAGG	ACGTTGGATGACTCACAGAGCACATTCACG	115	99.1	70.3	48	50	Sd	R	4801.1	ACATTCACGGTCACCT	G	5048.3	ACATTCACGGTCACCTC	A	5128.2	ACATTCACGGTCACCTT						
W1	iPLEX	rs1800890	ACGTTGGATGCAAGCCCAGATGCATAGTAG	ACGTTGGATGCTGATTTCCCAGTACATCCC	110	99.6	70.5	46	50	D	R	4819.2	CCCCCACTGGAAAAAT	T	5090.4	CCCCCACTGGAAAAATA	A	5146.3	CCCCCACTGGAAAAATT						
W1	iPLEX	rs1800896	ACGTTGGATGCCAAGACAACACTACTAAGG	ACGTTGGATGATTCCATGGAGGCTGGATAG	111	98.1	70.5	47	58.8		F	4977.2	CCTATCCCTACTTCCCC	C	5224.4	CCTATCCCTACTTCCCCC	T	5304.3	CCTATCCCTACTTCCCCT						
W1	iPLEX	rs8386	ACGTTGGATGTCATTTCACCTGCGCTGTGG	ACGTTGGATGTGCATGCGCTGAATGATGTC	98	98.5	70.5	52	56.3	D	R	5244.4	gTGAACACACGGCGGAT	T	5515.6	gTGAACACACGGCGGATA	C	5531.6	gTGAACACACGGCGGATG						
W1	iPLEX	rs17561	ACGTTGGATGTTTCACATTGCTCAGGAAGC	ACGTTGGATGATCTGCACTTGTGATCATGG	96	85.9	70.5	48	44.4	Dh	F	5467.6	ATCATCAAGCCTAGGTCA	G	5754.8	ATCATCAAGCCTAGGTCAG	T	5794.7	ATCATCAAGCCTAGGTCAT						
W1	iPLEX	rs8176719	ACGTTGGATGTGTCGATGTTGAATGTGCCC	ACGTTGGATGCATGTGCAGTAGGAAGGATG	99	100	71.7	52	55.6	d	F	5570.6	GAAGGATGTCCTCGTGGT	D	5841.8	GAAGGATGTCCTCGTGGTA	I	5857.8	GAAGGATGTCCTCGTGGTG						
W1	iPLEX	rs9282799	ACGTTGGATGTGGACAGTGGTAGCAAAGTG	ACGTTGGATGTTAGGGAGAAGTTGAGAAGC	98	98.5	70.5	48	52.9	d	R	5649.7	ttTCACCCTTGATCTCACC	T	5920.9	ttTCACCCTTGATCTCACCA	C	5936.9	ttTCACCCTTGATCTCACCG						
W1	iPLEX	rs334	ACGTTGGATGTCAAACAGACACCATGGTGC	ACGTTGGATGCAACTTCATCCACGTTCACC	106	99.9	70.5	49	52.9	d	R	5723.7	ctTAACGGCAGACTTCTCC	T	5994.9	ctTAACGGCAGACTTCTCCA	A	6050.8	ctTAACGGCAGACTTCTCCT						
W1	iPLEX	rs229587	ACGTTGGATGAAGGCCGCAATGAGAGAGAC	ACGTTGGATGCAAAGCATGTCCCTCATTAC	94	97	70.5	56	57.9	D	F	5819.8	CACGAGGCGCTGGTTTTCA	C	6067	CACGAGGCGCTGGTTTTCAC	T	6146.9	CACGAGGCGCTGGTTTTCAT						
W1	iPLEX	rs1050829	ACGTTGGATGTAGAAGAGGCGGTTGGCCTG	ACGTTGGATGAGTACGATGATGCAGCCTCC	105	92.9	70.5	48	56.3	DsH	R	5966.9	ccctCCTCAACAGCCACATG	T	6238.1	ccctCCTCAACAGCCACATGA	C	6254.1	ccctCCTCAACAGCCACATGG						
W1	iPLEX	rs3024500	ACGTTGGATGTCTCTGATCAGGTTTGGAGC	ACGTTGGATGGAGTGGGTGAATGAATTCTG	111	98.1	70.5	52	68.8	d	R	5967.9	ccctGAAGCCAGCTACCCCC	G	6215.1	ccctGAAGCCAGCTACCCCCC	A	6295	ccctGAAGCCAGCTACCCCCT						
W1	iPLEX	rs2227485	ACGTTGGATGGTTTTGTCTTAGTAGAGTTC	ACGTTGGATGCTGGTAGGAAAATGAGTCCG	116	86.9	70.5	48	40	D	R	6085	TGACCAAAATGCTTACTCAG	G	6332.2	TGACCAAAATGCTTACTCAGC	A	6412.1	TGACCAAAATGCTTACTCAGT						
W1	iPLEX	rs909253	ACGTTGGATGGTCAGAGAAACCCCAAGGTG	ACGTTGGATGTCTCTGTCACACATTCTCTG	115	92	70.5	49	45	DH	R	6314.1	cCATTCTCTGTTTCTGCCATG	T	6585.3	cCATTCTCTGTTTCTGCCATGA	C	6601.3	cCATTCTCTGTTTCTGCCATGG						
W1	iPLEX	rs3211938	ACGTTGGATGCTCCAGTTGAAAACCCAGAC	ACGTTGGATGTTGCATTTGCTGATGTCTAG	117	87.4	70.5	47	52.9	dh	R	6372.2	gtttCTGATGTCTAGCACACC	G	6619.3	gtttCTGATGTCTAGCACACCC	T	6643.4	gtttCTGATGTCTAGCACACCA						
W1	iPLEX	rs2227491	ACGTTGGATGTTCCACGGAGTCAGTGTAAG	ACGTTGGATGATCACCACCACCCCAAGTAC	98	89.3	70.5	61	76.5	Dh	F	6458.2	gggcTGGACGGCACACGGCCC	C	6705.4	gggcTGGACGGCACACGGCCCC	T	6785.3	gggcTGGACGGCACACGGCCCT						
W1	iPLEX	rs3093662	ACGTTGGATGGGAAAGAGCTGTTGAATGCC	ACGTTGGATGGTGTCTGGTTTTCTCTCTCC	84	99	70.5	46	40	d	R	6568.31	ttGTTTTCTCTCTCCATTCATC	G	6815.5	ttGTTTTCTCTCTCCATTCATCC	A	6895.4	ttGTTTTCTCTCTCCATTCATCT						
W1	iPLEX	rs17047661	ACGTTGGATGATGCACAGCTCCAGAAGTTG	ACGTTGGATGCCCGGGCTGACATCTAAATC	117	97.4	70.5	49	52.6	D	R	6925.5	aaacCTCCTGTTTCCTGGTACTC	G	7172.7	aaacCTCCTGTTTCCTGGTACTCC	A	7252.6	aaacCTCCTGTTTCCTGGTACTCT						
W1	iPLEX	rs1050828	ACGTTGGATGTCACTCTGTTTGCGGATGTC	ACGTTGGATGATGGCCTTCTGCCCGAAAAC	102	98.5	70.5	52	52.6	d	R	6928.5	ttcgGCCCGAAAACACCTTCATC	T	7199.7	ttcgGCCCGAAAACACCTTCATCA	C	7215.7	ttcgGCCCGAAAACACCTTCATCG						
W1	iPLEX	rs4986791	ACGTTGGATGGGTAATAACACCATTGAAGC	ACGTTGGATGAGGTTGCTGTTCTCAAAGTG	96	92.9	70.5	52	34.8	dH	F	7077.6	TTCTCAAAGTGATTTTGGGACAA	C	7324.8	TTCTCAAAGTGATTTTGGGACAAC	T	7404.7	TTCTCAAAGTGATTTTGGGACAAT						
W1	iPLEX	rs1555498	ACGTTGGATGCAATCATCAGAGTTCAAGGC	ACGTTGGATGGATGGAATAGCCCATCACAG	113	97.9	70.5	46	26.1		F	7130.7	AAAAAGAAACACGGTAATAGATA	C	7377.9	AAAAAGAAACACGGTAATAGATAC	T	7457.8	AAAAAGAAACACGGTAATAGATAT						
W1	iPLEX	rs1799964	ACGTTGGATGGGGAAGCAAAGGAGAAGCTG	ACGTTGGATGTACATGTGGCCATATCTCCC	112	98	70.5	57	54.2	s	R	7150.6	CCTCCAGACCCTGACTTTTCCTTC	T	7421.9	CCTCCAGACCCTGACTTTTCCTTCA	C	7437.9	CCTCCAGACCCTGACTTTTCCTTCG						
W1	iPLEX	rs2239704	ACGTTGGATGAGGTGCAGGAGGGACCGAG	ACGTTGGATGGTGCTTCGTGCTTTGGACTA	91	80.5	70.5	59	65	D	R	7296.7	gctgCGTGCTTTGGACTACCGCCC	G	7543.9	gctgCGTGCTTTGGACTACCGCCCC	T	7567.9	gctgCGTGCTTTGGACTACCGCCCA						
W1	iPLEX	rs2297518	ACGTTGGATGTTGTTGTTGAGCTCTTTCAG	ACGTTGGATGTTGAGAACTCTGTCATTCCC	95	92.9	70.5	47	30.4		R	7359.8	gCTCTTTCTAGAAACTGAAGAAAT	G	7607	gCTCTTTCTAGAAACTGAAGAAATC	A	7686.9	gCTCTTTCTAGAAACTGAAGAAATT						
W1	iPLEX	rs1012356	ACGTTGGATGCCCCGATCTCTTTTATACAG	ACGTTGGATGGCTTACCAATTCAGACTTCC	107	92.7	70.5	49	34.8		F	7510.9	tcCCAATTCAGACTTCCATTTAACT	A	7782.1	tcCCAATTCAGACTTCCATTTAACTA	T	7838	tcCCAATTCAGACTTCCATTTAACTT						
W1	iPLEX	rs187084	ACGTTGGATGTATTCCCCTGCTGGAATGTC	ACGTTGGATGTGCTGGGCACTGTACTGGAT	100	91.8	70.5	56	44	ds	F	7659	GATGCAGATAAAAGATCACTGCCCT	C	7906.2	GATGCAGATAAAAGATCACTGCCCTC	T	7986.1	GATGCAGATAAAAGATCACTGCCCTT						
W1	iPLEX	rs2230739	ACGTTGGATGGAAGGTGGGACTAGCAAACG	ACGTTGGATGTCCCAGTCATTCTGCTCCAC	100	97.1	70.5	59	56.5	d	F	7811.1	ctaCACTCCTGCTCCACACAGGTCAT	A	8082.3	ctaCACTCCTGCTCCACACAGGTCATA	G	8098.3	ctaCACTCCTGCTCCACACAGGTCATG						
W1	iPLEX	rs2814778	ACGTTGGATGAGACAGAAGGGCTGGGACG	ACGTTGGATGAACCTGATGGCCCTCATTAG	97	89.3	70.5	60	50		R	7879.1	TGGCCCTCATTAGTCCTTGGCTCTTA	G	8126.3	TGGCCCTCATTAGTCCTTGGCTCTTAC	A	8206.2	TGGCCCTCATTAGTCCTTGGCTCTTAT						
W1	iPLEX	rs2535611	ACGTTGGATGGAGTGAAACTCTGTCTCAAG	ACGTTGGATGCGACATTGTAGAACCAGGAC	92	98.3	70.5	57	56.5	D	F	7932.1	ggcCCAGGACCTGTCAGGTACACTTC	C	8179.3	ggcCCAGGACCTGTCAGGTACACTTCC	T	8259.2	ggcCCAGGACCTGTCAGGTACACTTCT						
W1	iPLEX	rs4986790	ACGTTGGATGAGCATACTTAGACTACTACC	ACGTTGGATGCACCAGGGAAAATGAAGAAAC	99	89.2	70.5	45	18.5		R	8291.5	GTCAAACAATTAAATAAGTCAATAATA	G	8538.7	GTCAAACAATTAAATAAGTCAATAATAC	A	8618.6	GTCAAACAATTAAATAAGTCAATAATAT						
W1	iPLEX	rs1805015	ACGTTGGATGTCTCTGGGACACGGTGACTG	ACGTTGGATGACCTGACTTGCACAGAGACG	114	92.3	70.5	64	57.1		F	8438.5	CAACCCTGCTTACCGCAGCTTCAGCAAC	C	8685.7	CAACCCTGCTTACCGCAGCTTCAGCAACC	T	8765.6	CAACCCTGCTTACCGCAGCTTCAGCAACT						
W2	iPLEX	rs1800629	ACGTTGGATGGGAGGCAATAGGTTTTGAGG	ACGTTGGATGCTGATTTGTGTGTAGGACCC	86	99.2	73.9	52	73.3	D	R	4513.9	GGCTGAACCCCGTCC	G	4761.1	GGCTGAACCCCGTCCC	A	4841	GGCTGAACCCCGTCCT						
W2	iPLEX	rs2075820	ACGTTGGATGAAGCGCAGCAGGAAGGCAAAC	ACGTTGGATGACCTGCTCTTCAAGCACTAC	90	92.5	73.9	55	80	DsH	F	4861.2	aCCAGAGCGGGACCCC	A	5132.4	aCCAGAGCGGGACCCCA	G	5148.4	aCCAGAGCGGGACCCCG						
W2	iPLEX	rs2242665	ACGTTGGATGGGCTCTCCCTTCCAGCTCT	ACGTTGGATGTGGTGGTGTCATTGGTGATC	107	79.8	73.9	54	62.5	h	R	4971.2	AGCGCCGGTGGAGTAA	G	5218.4	AGCGCCGGTGGAGTAAC	A	5298.3	AGCGCCGGTGGAGTAAT						
W2	iPLEX	rs1800482	ACGTTGGATGGGGCAGATCACTTGAGCTTC	ACGTTGGATGCCACCACACCCAGCTAATAT	119	94.7	73.9	49	50	D	R	5198.4	tTGTTGTCCATGCTGGT	G	5445.6	tTGTTGTCCATGCTGGTC	C	5485.6	tTGTTGTCCATGCTGGTG						
W2	iPLEX	rs6780995	ACGTTGGATGTCCCCTGGAGAAACATTTTG	ACGTTGGATGGTTCTTTGGTCTGTTGCAGG	92	94	73.9	46	47.1	d	R	5261.5	GGAGCAAACTACAGAGA	G	5508.6	GGAGCAAACTACAGAGAC	A	5588.6	GGAGCAAACTACAGAGAT						
W2	iPLEX	rs542998	ACGTTGGATGACTCTTTGGGTTCTGGAGTG	ACGTTGGATGCTCTGCCAGTCCATTTCATC	99	100	73.9	46	47.1	d	R	5274.4	GGTGGTCATCAGGTAAA	T	5545.7	GGTGGTCATCAGGTAAAA	C	5561.7	GGTGGTCATCAGGTAAAG						
W2	iPLEX	rs17047660	ACGTTGGATGATCAAGTTGGTGTTTGGAGC	ACGTTGGATGGCATTTTCAACTTCTGGAGC	99	97.1	73.9	46	53.3	d	R	5466.6	cccCTGGAGCTGTGCATT	G	5713.7	cccCTGGAGCTGTGCATTC	A	5793.6	cccCTGGAGCTGTGCATTT						
W2	iPLEX	rs461645	ACGTTGGATGGTTCCCAACAGGTAGACAAG	ACGTTGGATGAAAGACGGCTTCTCAGATCC	115	99.1	73.9	45	44.4	D	R	5467.6	ATCTACACTCTGGAGAAC	T	5738.8	ATCTACACTCTGGAGAACA	C	5754.8	ATCTACACTCTGGAGAACG						
W2	iPLEX	rs8078340	ACGTTGGATGTCTGTAGTGATGGAGGGATG	ACGTTGGATGGTGTCCTCTCCCTTGTAAAC	101	100	73.9	46	47.1	d	F	5525.6	aCTTGAACAAGGCAGAAC	C	5772.8	aCTTGAACAAGGCAGAACC	T	5852.7	aCTTGAACAAGGCAGAACT						
W2	iPLEX	rs1803632	ACGTTGGATGTCCTCAGCCATTCCAATGTC	ACGTTGGATGTAGAGAGAGTGACTCACGAG	94	98.7	73.9	53	61.1	d	F	5813.8	gCACGAGGCCCATTGACTG	C	6061	gCACGAGGCCCATTGACTGC	G	6101	gCACGAGGCCCATTGACTGG						
W2	iPLEX	rs2243250	ACGTTGGATGTAACAGGCAGACTCTCCTAC	ACGTTGGATGTGATACGACCTGTCCTTCTC	102	100	73.9	47	38.9	DH	F	5891.9	gAACTTGGGAGAACATTGT	C	6139	gAACTTGGGAGAACATTGTC	T	6218.9	gAACTTGGGAGAACATTGTT						
W2	iPLEX	rs2706384	ACGTTGGATGGGTATATCTCCCGAACGCAG	ACGTTGGATGGAGATTCGGCCCAGTGTTC	99	94.6	73.9	57	70.6	dH	F	6118	attCAAGTGCCCGGGCGATC	C	6365.2	attCAAGTGCCCGGGCGATCC	A	6389.2	attCAAGTGCCCGGGCGATCA						
W2	iPLEX	rs1801033	ACGTTGGATGGGAATGCATGGTTGAAAGGC	ACGTTGGATGGTTAGATCTGTCTTGCGTCC	100	99	73.9	54	64.7	dH	F	6158	tgtGGGACAGCCATGCACTG	C	6405.2	tgtGGGACAGCCATGCACTGC	A	6429.2	tgtGGGACAGCCATGCACTGA						
W2	iPLEX	rs708567	ACGTTGGATGGTTCTCCTCACCATTCCTAG	ACGTTGGATGGAGGAAAAGTTTGGAGGAGC	83	92.6	73.9	53	58.8	Dh	R	6247.1	aggTGGAGGAGCAGCTGACT	G	6494.2	aggTGGAGGAGCAGCTGACTC	A	6574.2	aggTGGAGGAGCAGCTGACTT						
W2	iPLEX	rs7935564	ACGTTGGATGACAGGAAAAGCTGCAGGTAG	ACGTTGGATGTTCCAGGCGGTTCTCTCTTG	119	97.1	73.9	49	50		R	6284.1	cccGCTTCTCAGCCTCTTGAT	G	6531.3	cccGCTTCTCAGCCTCTTGATC	A	6611.2	cccGCTTCTCAGCCTCTTGATT						
W2	iPLEX	rs1143634	ACGTTGGATGCAGTTCAGTGATCGTACAGG	ACGTTGGATGGTGCTCCACATTTCAGAACC	106	99.9	73.9	46	33.3	D	F	6306.1	CATTTCAGAACCTATCTTCTT	C	6553.3	CATTTCAGAACCTATCTTCTTC	T	6633.2	CATTTCAGAACCTATCTTCTTT						
W2	iPLEX	rs1126535	ACGTTGGATGGGCTTGTGGTTCATCTTACC	ACGTTGGATGATCACCCAGATGATTGGGTC	99	88.2	73.7	48	42.9	h	F	6476.2	GCTGTGTATCTTCATAGAAGG	C	6723.4	GCTGTGTATCTTCATAGAAGGC	T	6803.3	GCTGTGTATCTTCATAGAAGGT						
W2	iPLEX	rs8176746	ACGTTGGATGTTGCACCGACCCCCCGAAGAA	ACGTTGGATGCCCAGTCCCAGGCCTACAT	97	75.3	73.9	52	50		F	6695.4	ccACGAGGGCGATTTCTACTAC	C	6942.5	ccACGAGGGCGATTTCTACTACC	A	6966.6	ccACGAGGGCGATTTCTACTACA						
W2	iPLEX	rs1128127	ACGTTGGATGGGCAGGTAATTGGGGTCTTC	ACGTTGGATGTGTGCTGGCCTGTGCTCAAC	103	93	73.9	55	52.4	Dg	R	6768.4	aACAGAAAGCTGCTCCTGGATG	G	7015.6	aACAGAAAGCTGCTCCTGGATGC	A	7095.5	aACAGAAAGCTGCTCCTGGATGT						
W2	iPLEX	rs373533	ACGTTGGATGTCCTTTGTGGGCATGGAATC	ACGTTGGATGTGATCTCAGAGGTGGTCAAG	99	100	73.9	57	60	DH	F	6847.4	ggCAAGGGAGCCTGGTGGTCTT	G	7134.6	ggCAAGGGAGCCTGGTGGTCTTG	T	7174.5	ggCAAGGGAGCCTGGTGGTCTTT						
W2	iPLEX	rs2227507	ACGTTGGATGAACAGCAAATCCAGTTCTCC	ACGTTGGATGGAAAACATGGCATAAATGCTC	119	87.6	73.9	50	40.9		R	7053.6	aCTTATTTTCACAGCTTGGAGAG	T	7324.8	aCTTATTTTCACAGCTTGGAGAGA	C	7340.8	aCTTATTTTCACAGCTTGGAGAGG						
W2	iPLEX	rs5743810	ACGTTGGATGATTTTTATCAGAACTCACC	ACGTTGGATGAGGCATTTCCAAGTCGTTTC	98	78.3	73.7	50	39.1	d	R	7191.7	GAGGGTAAAATTCAGTAAGGTTG	T	7462.9	GAGGGTAAAATTCAGTAAGGTTGA	C	7478.9	GAGGGTAAAATTCAGTAAGGTTGG						
W2	iPLEX	rs20541	ACGTTGGATGCCAGTTTGTAAAGGACCTGC	ACGTTGGATGTGATGCTTTCGAAGTTTCAG	100	94.4	73.9	51	40.9	dh	F	7358.8	cgGCTTTCGAAGTTTCAGTTGAAC	C	7606	cgGCTTTCGAAGTTTCAGTTGAACC	T	7685.9	cgGCTTTCGAAGTTTCAGTTGAACT						
W2	iPLEX	rs10775349	ACGTTGGATGGTTGGCTCTTCAGTGAAGTG	ACGTTGGATGAAAACCAGACCACTCAGTAG	86	97.8	73.9	51	33.3		R	7377.8	ACCACTCAGTAGAAAAATGTGTAA	G	7625	ACCACTCAGTAGAAAAATGTGTAAC	C	7665.1	ACCACTCAGTAGAAAAATGTGTAAG						
W2	iPLEX	rs3092945	ACGTTGGATGCAGATCTCTTAACTGCAGCC	ACGTTGGATGCTGTGTACACTGTTCCAATC	109	98.2	73.9	49	32		R	7646	GTTCCAATCCATTAGATAATTGTTG	T	7917.2	GTTCCAATCCATTAGATAATTGTTGA	C	7933.2	GTTCCAATCCATTAGATAATTGTTGG						
W2	iPLEX	rs5743809	ACGTTGGATGGCGAATAAACTAGTTGGGTG	ACGTTGGATGAATGAGACAGAAAGTCTAC	94	86.5	73.7	49	33.3	D	F	7707.1	gATGAGACAGAAAGTCTACAAATTC	C	7954.2	gATGAGACAGAAAGTCTACAAATTCC	T	8034.1	gATGAGACAGAAAGTCTACAAATTCT						
W2	iPLEX	rs4833095	ACGTTGGATGACTTCCTAAGTATTCTGGCG	ACGTTGGATGCCAAGTTGTTTCAATGTTGT	100	84.3	73.7	50	28	Ds	F	7741.1	TGTTTCAATGTTGTTTAAGGTAAGA	C	7988.2	TGTTTCAATGTTGTTTAAGGTAAGAC	T	8068.2	TGTTTCAATGTTGTTTAAGGTAAGAT						
W3	iPLEX	rs1566830	ACGTTGGATGGAGTGGCAATGGGCATAATC	ACGTTGGATGAACCCTGTATCTGCCTTTGG	117	98.9	68.8	46	53.3	D	F	4568	GCCTTTGGTCACAGA	G	4855.2	GCCTTTGGTCACAGAG	T	4895.1	GCCTTTGGTCACAGAT						
W3	iPLEX	rs11899121	ACGTTGGATGAGAAAGGACCACATCATGGG	ACGTTGGATGTCAAAGGCCCCAGAGAGAAG	119	97.1	68.8	51	62.5	Ds	F	4753.1	CTCAGCCTGCACCTCT	C	5000.3	CTCAGCCTGCACCTCTC	G	5040.3	CTCAGCCTGCACCTCTG						
W3	iPLEX	rs7663712	ACGTTGGATGTTCCTCTATTCTGACAGCCG	ACGTTGGATGGGTGTAGCACTGGTGTTTTC	112	99.4	68.8	46	50	D	F	4930.2	AGTCCTGGAAGACAGT	A	5201.4	AGTCCTGGAAGACAGTA	G	5217.4	AGTCCTGGAAGACAGTG						
W3	iPLEX	rs113788643	ACGTTGGATGAGAGCTTCTACAACAGGAGG	ACGTTGGATGCCATGGAAACACATCACAGG	97	100	68.8	46	60	DH	F	4968.2	tGGGAGTGGGTCTACT	A	5239.4	tGGGAGTGGGTCTACTA	G	5255.4	tGGGAGTGGGTCTACTG						
W3	iPLEX	rs200704287	ACGTTGGATGTCAGCTGCAAAGGCCATTTC	ACGTTGGATGATACCCTTGGCCTGTGCTTG	99	98.6	68.8	51	52.9	d	R	5023.3	TTGCCCTTTCCTACCCT	T	5294.5	TTGCCCTTTCCTACCCTA	C	5310.5	TTGCCCTTTCCTACCCTG						
W3	iPLEX	rs76924464	ACGTTGGATGCAGGATTCAGTCAGTAAAGC	ACGTTGGATGAACCCTGGCTTAAGTGGAAG	96	98.5	68.8	47	47.1	d	R	5274.4	AAGTGGAAGATTGCCTG	G	5521.6	AAGTGGAAGATTGCCTGC	A	5601.5	AAGTGGAAGATTGCCTGT						
W3	iPLEX	rs10900585	ACGTTGGATGATTGCACTCCAGCCTGCCT	ACGTTGGATGCACTTTTAATGTAGTTCTCC	135	78.2	83.2	47	44.4		F	5498.6	AGAAGGAGTCTCACTCTT	G	5785.8	AGAAGGAGTCTCACTCTTG	T	5825.7	AGAAGGAGTCTCACTCTTT						
W3	iPLEX	rs116472045	ACGTTGGATGACATAGGCCTTTCAGCTTGG	ACGTTGGATGATATGCTGGCAAAGCACTTG	102	98.5	61.8	48	44.4	D	R	5556.6	GCAAAGCACTTGGAAATG	G	5803.8	GCAAAGCACTTGGAAATGC	A	5883.7	GCAAAGCACTTGGAAATGT						
W3	iPLEX	rs116288147	ACGTTGGATGATGATGACCCCAGGTAACTC	ACGTTGGATGTGAGAAATGCTGGACCGAGG	104	94.2	68.8	47	60	d	R	5572.6	aaaGGTGATGGCATCAGC	T	5843.9	aaaGGTGATGGCATCAGCA	C	5859.9	aaaGGTGATGGCATCAGCG						
W3	iPLEX	rs72933350	ACGTTGGATGGAGAGATCAAGCCAGGAAAC	ACGTTGGATGTTACTAAACCCACCAGAGCC	118	98.7	74.8	52	52.6	D	F	5674.7	CCACTTCAGGCCCTCTTTA	C	5921.9	CCACTTCAGGCCCTCTTTAC	T	6001.8	CCACTTCAGGCCCTCTTTAT						
W3	iPLEX	rs192909543	ACGTTGGATGGAGCATTTCACTGTGATGAG	ACGTTGGATGGCTCTTCAGATATGTGTGAC	100	97.1	68.8	50	47.4	D	F	5689.7	ACCTGTCCTCAAGCTTTTC	G	5976.9	ACCTGTCCTCAAGCTTTTCG	T	6016.8	ACCTGTCCTCAAGCTTTTCT						
W3	iPLEX	rs138826089	ACGTTGGATGCACAGTCCCCTGCTAAAGTT	ACGTTGGATGGTACATACCCTAGGGCACAC	101	91.8	68.8	50	42.1	Ds	R	5767.8	GGCACACACCAAATCAAAA	T	6039	GGCACACACCAAATCAAAAA	C	6055	GGCACACACCAAATCAAAAG						
W3	iPLEX	rs15116938	ACGTTGGATGATTGCCTTAGCTAAGGGGTG	ACGTTGGATGATTATAGGCATGGGCCACCG	113	97.9	68.8	47	35	Dg	F	6073	TTAGGTTCTTTTTCACCAGA	C	6320.2	TTAGGTTCTTTTTCACCAGAC	T	6400.1	TTAGGTTCTTTTTCACCAGAT						
W3	iPLEX	rs1371474	ACGTTGGATGGGCCGTGTTTCCTAAATTAC	ACGTTGGATGAGTGCACCAGAGCAAAAAAG	101	97.1	68.8	51	60	DgH	F	6142	aaagcATTGTCCTGGGGCCA	C	6389.2	aaagcATTGTCCTGGGGCCAC	T	6469.1	aaagcATTGTCCTGGGGCCAT						
W3	iPLEX	rs3742785	ACGTTGGATGCTTGTGATTTCAGACTTGGG	ACGTTGGATGACTTGCTCTAAGGGGCAGAC	116	91.9	68.8	55	55	g	F	6179	tCATTCCTTCCTCCCCCCATT	C	6426.2	tCATTCCTTCCTCCCCCCATTC	A	6450.2	tCATTCCTTCCTCCCCCCATTA						
W3	iPLEX	rs146428334	ACGTTGGATGCTAAAGCATTTTGTACTGAC	ACGTTGGATGACCGAGAGAAGTAAATTTGC	95	82.7	68.8	46	33.3	D	R	6346.1	TGCATAAACTATTCTTGTCTC	G	6593.3	TGCATAAACTATTCTTGTCTCC	A	6673.2	TGCATAAACTATTCTTGTCTCT						
W3	iPLEX	rs10139016	ACGTTGGATGGCATCACCAGTTACCATCAG	ACGTTGGATGGTATTTGGCAACTGGGTTTC	120	97	68.8	46	33.3	D	F	6493.3	TATTGAGTAGATAGGACCAAT	C	6740.4	TATTGAGTAGATAGGACCAATC	T	6820.4	TATTGAGTAGATAGGACCAATT						
W3	iPLEX	rs12107243	ACGTTGGATGGGAATCTGGGACAGTAAAGG	ACGTTGGATGAAAATTGCAGTCCCTATTCC	115	93.5	68.8	48	31.8	D	F	6699.4	ACATGAGATCTCATTTATTGCT	C	6946.6	ACATGAGATCTCATTTATTGCTC	G	6986.6	ACATGAGATCTCATTTATTGCTG						
W3	iPLEX	rs73991577	ACGTTGGATGTTGTAGAAGTACCTCCCTGC	ACGTTGGATGACTGCTTTTTCTGAGAGCCC	114	99.2	68.8	53	50	h	R	6719.4	GAGAGCCCAATAGCTCATCTTG	G	6966.6	GAGAGCCCAATAGCTCATCTTGC	A	7046.5	GAGAGCCCAATAGCTCATCTTGT						
W3	iPLEX	rs4266246	ACGTTGGATGAGCCAATATACATCTAGAGG	ACGTTGGATGCTTGCAACCTGCCACATAGC	100	93	68.8	46	36.4	D	F	6782.4	CTGTCCATAATATGGAAGTAAG	C	7029.6	CTGTCCATAATATGGAAGTAAGC	T	7109.5	CTGTCCATAATATGGAAGTAAGT						

W3	iPLEX	rs148111931	ACGTTGGATGTGGTTTCTTGGTAGGTTGGG	ACGTTGGATGGAGCTGTGATGGAGTCACTG	106	98.4	68.8	53	52.4	dg	F	6864.5	gGGGTGATAGAGTGAAACCCTG	A	7135.7	gGGGTGATAGAGTGAAACCCTGA	T	7191.6	gGGGTGATAGAGTGAAACCCTGT			
W3	iPLEX	rs182873742	ACGTTGGATGCTCTTGCTCATGTGTATCTG	ACGTTGGATGAGGAATGAGCATAGGGAGAG	104	98.5	68.8	52	43.5	D	F	6927.5	TGTCATCTCCTCAAACTAGACAC	C	7174.7	TGTCATCTCCTCAAACTAGACACC	G	7214.7	TGTCATCTCCTCAAACTAGACACG			
W3	iPLEX	rs184895969	ACGTTGGATGAGGGTCCCCAAATTTTTATC	ACGTTGGATGTGACCTAATCACCTCCCAAC	100	90.2	68.8	47	30.4	D	R	7012.6	CAGTTTTTAATGACATCACATTG	T	7283.8	CAGTTTTTAATGACATCACATTGA	C	7299.8	CAGTTTTTAATGACATCACATTGG			
W3	iPLEX	rs114726617	ACGTTGGATGTCCAATCCAAGGAGCAGAG	ACGTTGGATGCTCTGTAAGCTCCAAGGCAT	119	94.6	68.8	50	30.4	DH	F	7079.6	AAAAAGTATTTTGGGTACAACCA	A	7350.9	AAAAAGTATTTTGGGTACAACCAA	G	7366.9	AAAAAGTATTTTGGGTACAACCAG			
W3	iPLEX	rs79691057	ACGTTGGATGGGCATAACTACCTACTTCAC	ACGTTGGATGGCATAGTGATGACCATGGAC	136	93.6	68.8	53	37.5	DA	R	7246.7	AACTTAGCATTGACTTCATCCTCA	G	7493.9	AACTTAGCATTGACTTCATCCTCAC	A	7573.8	AACTTAGCATTGACTTCATCCTCAT			
W3	iPLEX	rs74063230	ACGTTGGATGATTCTTCCCAGACTGGCTCC	ACGTTGGATGCCAGGCTGAGATAAAATGAG	111	96.7	68.8	49	37.5	D	F	7473.9	CTGAGATAAAATGAGAAGTCTAGG	A	7745.1	CTGAGATAAAATGAGAAGTCTAGGA	G	7761.1	CTGAGATAAAATGAGAAGTCTAGGG			
W3	iPLEX	rs10188961	ACGTTGGATGGCAATGTTTAGAATTCAGAG	ACGTTGGATGATCACCTCTACTCTCTTCAC	95	86.4	68.8	48	24		R	7601	ACTTCAAAAATAACTGCAACTATTA	G	7848.2	ACTTCAAAAATAACTGCAACTATTAC	A	7928.1	ACTTCAAAAATAACTGCAACTATTAT			
W3	iPLEX	rs6674631	ACGTTGGATGATGTCTGTTACTATCCTCCC	ACGTTGGATGCTGGGCTTTTGACCTTGATG	93	98.4	68.8	59	52	D	F	7624	TGGATTTGTTAGAAGTGCCCTCCCC	A	7895.2	TGGATTTGTTAGAAGTGCCCTCCCCA	G	7911.2	TGGATTTGTTAGAAGTGCCCTCCCCG			
W3	iPLEX	rs115947774	ACGTTGGATGATCCAATCAAGGATCATAG	ACGTTGGATGTTCCAATGCAAGGAATGCCC	119	86.7	68.8	56	42.3	D	R	7803.1	TAACTAGATTAACCCCCCAATCCTCA	G	8050.3	TAACTAGATTAACCCCCCAATCCTCAC	A	8130.2	TAACTAGATTAACCCCCCAATCCTCAT			
W3	iPLEX	rs2131263	ACGTTGGATGCCTCTGTATCTGAAACCAAC	ACGTTGGATGGCCTTACATGTAACTACTCAC	128	90.3	68.8	50	28	DA	F	7946.2	gTGATCCAAAATTTAGAAACTTCACA	C	8193.4	gTGATCCAAAATTTAGAAACTTCACAC	T	8273.3	gTGATCCAAAATTTAGAAACTTCACAT			
W3	iPLEX	rs4316902	ACGTTGGATGGTTCCCATGGTATTCATAA	ACGTTGGATGGGCTTTTGATTAAAAGGGCAG	118	80	68.8	45	15.4	D	F	8082.3	AGAAATTTAAAAGTAATGTGAAAAAT	A	8353.5	AGAAATTTAAAAGTAATGTGAAAAATA	G	8369.5	AGAAATTTAAAAGTAATGTGAAAAATG			
W3	iPLEX	rs55958968	ACGTTGGATGGCAACAACTCCAGGAGTTTG	ACGTTGGATGCTTTGGCCTCTCTTGGATTG	133	95.7	68.8	46	18.5	DA	F	8237.4	GTTTTCTACCTTTAAAATGAATTTAAT	A	8508.6	GTTTTCTACCTTTAAAATGAATTTAATA	G	8524.6	GTTTTCTACCTTTAAAATGAATTTAATG			
W3	iPLEX	rs1371478	ACGTTGGATGTGCCTATTGTCCAATGTCAG	ACGTTGGATGATTGCTTCTGTCCAGCAAAG	98	97.1	68.8	55	37	D	F	8300.4	GCTTCTGTCCAGCAAAGAATTAGAAAA	C	8547.6	GCTTCTGTCCAGCAAAGAATTAGAAAAC	T	8627.5	GCTTCTGTCCAGCAAAGAATTAGAAAAT			
W3	iPLEX	rs186873296	ACGTTGGATGGATGTGAAGAAGCTGGGAAC	ACGTTGGATGTCCAGAGTAAGCATCCTTCC	100	100	68.8	52	27.6	d	R	8862.8	AGAATAAGCTTTCTTTGTCATTTCTTGTA	G	9110	AGAATAAGCTTTCTTTGTCATTTCTTGTAC	A	9189.9	AGAATAAGCTTTCTTTGTCATTTCTTGTAT			
W3	iPLEX	rs144778284	ACGTTGGATGTGCACTTATAGCCCCATTTC	ACGTTGGATGGGTAAATTACAAAGGGTGGG	107	97	68.8	60	44.8		R	8885.8	GGGTCCCTTTGCAAAGGAACATCACAATT	T	9157	GGGTCCCTTTGCAAAGGAACATCACAATTA	C	9173	GGGTCCCTTTGCAAAGGAACATCACAATTG			
W4	iPLEX	rs11016116	ACGTTGGATGTTGAGAGGACCATCTGTACC	ACGTTGGATGGCTTTCATCTCACATGTGCC	119	98.6	18.5	46	60	D	R	4522	CACTGTACCGAGCAC	G	4769.1	CACTGTACCGAGCACC	A	4849.1	CACTGTACCGAGCACT			
W4	iPLEX	rs8176750	ACGTTGGATGAAGCTGAGGTTCACTGCGG	ACGTTGGATGCAGCCCTCCCAGAGAAAATG	102	94.6	85	61	80	H	F	4578.9	GGCAGCCGCTCACGG	I	4866.1	GGCAGCCGCTCACGGG	D	4906	GGCAGCCGCTCACGGT			
W4	iPLEX	rs144224092	ACGTTGGATGAACTCCTGACCTCGTGATCC	ACGTTGGATGGTGGTATCACTTTTTACTG	136	82.3	18.5	47	60	DA	F	4633	CAGCACTTTGGGAGG	C	4880.2	CAGCACTTTGGGAGGC	T	4960.1	CAGCACTTTGGGAGGT			
W4	iPLEX	rs10459266	ACGTTGGATGTTCCAAGTCCCTCCCTTTTC	ACGTTGGATGTTCCAACAGAGGAGTGTCAG	112	99.4	78.4	46	53.3	dh	R	4832.2	tCTCATCTTGGCAGCA	C	5119.4	tCTCATCTTGGCAGCAG	A	5159.3	tCTCATCTTGGCAGCAT			
W4	iPLEX	rs192151845	ACGTTGGATGACACCCACACTCACTTCTGC	ACGTTGGATGTTAATGACAGACTTGGAGCG	112	96.6	18.5	55	68.8	D	R	5027.3	TTGGAGCGAGGAGGCG	G	5274.5	TTGGAGCGAGGAGGCGC	A	5354.4	TTGGAGCGAGGAGGCGT			
W4	iPLEX	rs77389579	ACGTTGGATGTGTCATCCCTGTGACCTAAG	ACGTTGGATGCCTGGCTGTGGAAAGATGAA	122	95.6	18.5	47	47.1	DA	F	5043.3	CTCCAACTCACACACAT	G	5330.5	CTCCAACTCACACACATG	T	5370.4	CTCCAACTCACACACATT			
W4	iPLEX	rs56390333	ACGTTGGATGTCCCAGAGCCCCTGGCAG	ACGTTGGATGGAAGCTGAGGTTCACTGCG	97	59.7	18.5	55	64.7	dh	R	5189.4	AGAACCACCAGGCGGTC	G	5436.6	AGAACCACCAGGCGGTCC	C	5476.6	AGAACCACCAGGCGGTCG	A	5516.5	AGAACCACCAGGCGGTCT
W4	iPLEX	rs973128	ACGTTGGATGATATTGGAGGCTTAGCCCAG	ACGTTGGATGGGCCTGTTTTCATCCCTTGC	107	98.4	18.5	55	55.6	Ds	R	5392.5	TTCATCCCTTGCCTGGCT	G	5639.7	TTCATCCCTTGCCTGGCTC	C	5679.7	TTCATCCCTTGCCTGGCTG			
W4	iPLEX	rs148494166	ACGTTGGATGGGATCTGTTGTAGGCAGAAG	ACGTTGGATGATTCTGGGAGATCTGCCCTG	111	98.1	18.5	46	47.1	d	F	5545.6	cCGTCTAGGGTTTTGGAA	C	5792.8	cCGTCTAGGGTTTTGGAAC	T	5872.7	cCGTCTAGGGTTTTGGAAT			
W4	iPLEX	rs55868763	ACGTTGGATGTAGCCGTCCGAAGTCTAGAT	ACGTTGGATGTTCCACTCAGTTCCCCCATC	101	96.1	18.5	52	52.6	DS	F	5723.7	CTCGCTGCCAGACTTCATA	C	5970.9	CTCGCTGCCAGACTTCATAC	G	6010.9	CTCGCTGCCAGACTTCATAG			
W4	iPLEX	rs4951074	ACGTTGGATGCAAGTTCATCATCCTCTGGC	ACGTTGGATGTTGCTCCCTATCTGCTAGAC	100	100	18.5	46	42.1	D	R	5820.8	CTCCTAGAGATAAAGCAGT	G	6068	CTCCTAGAGATAAAGCAGTC	A	6147.9	CTCCTAGAGATAAAGCAGTT			
W4	iPLEX	rs186790584	ACGTTGGATGATCTTCTGGAGAGCTTGATG	ACGTTGGATGTGGCCTTTAGTTGTTTCTAC	143	86.4	18.5	45	38.9	DA	F	5851.8	cTTAAAGCAGCTGATTGAG	A	6123	cTTAAAGCAGCTGATTGAGA	T	6178.9	cTTAAAGCAGCTGATTGAGT			
W4	iPLEX	rs76033371	ACGTTGGATGCCCAGTGTTGTTAGCCTTTC	ACGTTGGATGATCAGGCTTTCTAGTCTCCC	132	96	18.5	46	40	DA	R	6026.9	TTCTAGTCTCCCTTAACAAG	G	6274.1	TTCTAGTCTCCCTTAACAAGC	A	6354	TTCTAGTCTCCCTTAACAAGT			
W4	iPLEX	rs3753036	ACGTTGGATGTGGACCTCATTGTCAATGGC	ACGTTGGATGTTCCAACCACCCACTCTAAG	120	98.4	78.4	47	44.4	d	R	6045	aaACCCACTCTAAGTGTCTA	G	6292.1	aaACCCACTCTAAGTGTCTAC	A	6372.1	aaACCCACTCTAAGTGTCTAT			
W4	iPLEX	rs12307123	ACGTTGGATGTCTGGAGAGACAATCAGAAC	ACGTTGGATGGCCAGTTCAACTTATCCCTG	114	97.8	18.5	46	40	d	F	6089	TGTTTATACTAGCTCTGGTC	C	6336.2	TGTTTATACTAGCTCTGGTCC	T	6416.1	TGTTTATACTAGCTCTGGTCT			
W4	iPLEX	rs4650365	ACGTTGGATGCTCAACATAAACATATGAGC	ACGTTGGATGCTGACAGTCTATAGAGTCCC	108	87.5	18.5	45	50	Ds	F	6317.1	tggCTGAACTCCCTACTACTC	C	6564.3	tggCTGAACTCCCTACTACTCC	T	6644.2	tggCTGAACTCCCTACTACTCT			
W4	iPLEX	rs141555199	ACGTTGGATGGTGCCATCCTTAATAAAAG	ACGTTGGATGTAGAGGGATTTGTCATGAAG	108	82.8	18.5	49	42.9	dh	F	6445.2	GTCTAGCTACATAGAAGCTGT	C	6692.4	GTCTAGCTACATAGAAGCTGTC	G	6732.4	GTCTAGCTACATAGAAGCTGTG			
W4	iPLEX	rs79100774	ACGTTGGATGCCCCCTGGATGGTTATATTC	ACGTTGGATGTAGAAGAATGATTGGTGGC	121	61.8	18.5	48	38.1	dA	F	6509.3	ATTGGTGGCTATAGCATAAAG	C	6756.4	ATTGGTGGCTATAGCATAAAGC	G	6796.5	ATTGGTGGCTATAGCATAAAGG			
W4	iPLEX	rs184908374	ACGTTGGATGGGTGGCATTTGTTTGGTTAC	ACGTTGGATGCACAACTCTGGTGATGAATG	100	97.1	18.5	51	38.1		R	6773.4	tTGGTGATGAATGCACCAATAT	G	7020.6	tTGGTGATGAATGCACCAATATC	A	7100.5	tTGGTGATGAATGCACCAATATT			
W4	iPLEX	rs149914432	ACGTTGGATGAAAGTACTGCCACTGCCAAC	ACGTTGGATGAGCAGAGGCAAGTTGGTAG	131	94.7	18.5	49	31.8	DAs	F	6873.5	AAGTTGGTAGCAAAAAAGAGAA	C	7120.7	AAGTTGGTAGCAAAAAAGAGAAC	A	7144.7	AAGTTGGTAGCAAAAAAGAGAAA			
W4	iPLEX	rs151293197	ACGTTGGATGGCAGAGGTCACAGATTACAC	ACGTTGGATGGATTTTTCTGTCTTCAAGC	101	87.9	18.5	52	38.1	d	F	7047.6	ggTGTCTTCAAGCAAATGCACAT	C	7294.8	ggTGTCTTCAAGCAAATGCACATC	G	7334.8	ggTGTCTTCAAGCAAATGCACATG			
W4	iPLEX	rs72933310	ACGTTGGATGCCTCTATGTCTTTTCATGGC	ACGTTGGATGGTAGGATATCTATGCAATGG	96	92.9	18.5	46	30.4	D	F	7070.6	GATATCTATGCAATGGAATACTA	C	7317.8	GATATCTATGCAATGGAATACTAC	T	7397.7	GATATCTATGCAATGGAATACTAT			
W4	iPLEX	rs6457452	ACGTTGGATGAAAACGGCCAGCCTGAGGAG	ACGTTGGATGGTTCGCTCTGGAAAGCCTTG	107	92.8	18.5	54	50	s	R	7081.6	tGGAGTCACTCTCGAAAGACGAA	T	7352.8	tGGAGTCACTCTCGAAAGACGAAA	C	7368.8	tGGAGTCACTCTCGAAAGACGAAG			
W4	iPLEX	rs72933304	ACGTTGGATGGGGAAAGCTATCCAAAGGTG	ACGTTGGATGATCTGTTTCTGCCCTTCACC	137	94.7	18.5	58	50	DA	F	7174.7	GTTTCTGCCCTTCACCCCTTACAA	C	7421.9	GTTTCTGCCCTTCACCCCTTACAAC	A	7445.9	GTTTCTGCCCTTCACCCCTTACAAA			
W4	iPLEX	rs28459062	ACGTTGGATGAGTCTAGTTCATGGAGAGGG	ACGTTGGATGAATCCAGTCCCATCACTTTC	108	98.3	18.5	47	30.4	D	R	7236.7	aCCATCACTTTCAATGTTATCTAT	T	7507.9	aCCATCACTTTCAATGTTATCTATA	C	7523.9	aCCATCACTTTCAATGTTATCTATG			
W4	iPLEX	rs1394263	ACGTTGGATGCTCATCACAGTGAAATGCTC	ACGTTGGATGTTGGACCTTAGCTGAGTGTG	123	96.5	18.5	46	26.1	DA	R	7463.9	gTGAATTAGAATGCATTAGTAAGA	T	7735.1	gTGAATTAGAATGCATTAGTAAGAA	C	7751.1	gTGAATTAGAATGCATTAGTAAGAG			
W4	iPLEX	rs13103597	ACGTTGGATGCCCTGCAAATCCTGTCAATC	ACGTTGGATGAAGGGCTACAAACGTTATGG	117	97.4	18.5	46	24		R	7654	TACAAACGTTATGGTTTTAAATATC	T	7925.2	TACAAACGTTATGGTTTTAAATATCA	C	7941.2	TACAAACGTTATGGTTTTAAATATCG			
W4	iPLEX	rs184142841	ACGTTGGATGGTTTTCTTTAGTACCACTCC	ACGTTGGATGTCACCAAAGAAGACATACGG	108	92.8	61.8	46	20	D	R	7656	ATGAACACATAAAAATATGTTCATT	G	7903.2	ATGAACACATAAAAATATGTTCATTC	A	7983.1	ATGAACACATAAAAATATGTTCATTT			
W4	iPLEX	rs75180423	ACGTTGGATGTGCCATTCCAGATGGCAAAC	ACGTTGGATGGAGGAACCTATTTCTAGTGAG	116	93.5	18.5	45	24	d	R	7672	GATAATTAGTGTCCTTACAAATAAA	C	7959.2	GATAATTAGTGTCCTTACAAATAAAG	A	7999.1	GATAATTAGTGTCCTTACAAATAAAT			
W4	iPLEX	rs1541255	ACGTTGGATGTAGCCGTCCGAAGTCTAGAT	ACGTTGGATGTTCCACTCAGTTCCCCCATC	101	96.1	18.5	57	50	DSH	R	7848.1	CCGTCCGAAGTCTAGATCCTTTCTTC	G	8095.3	CCGTCCGAAGTCTAGATCCTTTCTTCC	T	8119.3	CCGTCCGAAGTCTAGATCCTTTCTTCA	A	8175.2	CCGTCCGAAGTCTAGATCCTTTCTTCT
W4	iPLEX	rs142712208	ACGTTGGATGTTTCATTCTTCTGCAGACAC	ACGTTGGATGGTGTGTACTAACAATCACCAG	129	86.2	18.5	45	18.5	A	F	8295.4	CCATTATGATAATTGATGTATTAAAAT	A	8566.7	CCATTATGATAATTGATGTATTAAAATA	G	8582.7	CCATTATGATAATTGATGTATTAAAATG			
W4	iPLEX	rs114980857	ACGTTGGATGGTGACCTTTCCTCTAGGTCC	ACGTTGGATGGAGTCCAGGCTGATAAGGTC	85	96.3	18.5	56	44.4	d	F	8421.5	GATAAGGTCTCACATGGAGATGAGGAA	C	8668.7	GATAAGGTCTCACATGGAGATGAGGAAC	T	8748.6	GATAAGGTCTCACATGGAGATGAGGAAT			
W4	iPLEX	rs75731597	ACGTTGGATGGTTCACATAAGATATGACTG	ACGTTGGATGTGCTTTCCATCACAACCTG	113	85.3	85.3	45	14.3		F	8547.6	TGTTTTAATTAATATTTCTCTGAATTTA	C	8794.8	TGTTTTAATTAATATTTCTCTGAATTTAC	A	8818.8	TGTTTTAATTAATATTTCTCTGAATTTAA			
W4	iPLEX	rs74954675	ACGTTGGATGTTAAGGCCATGCAGAGGTTG	ACGTTGGATGATGTACATTTTAAAGAAGAG	135	66.3	18.5	47	21.4	dA	R	8611.7	GAAATAAATCTTTAAATGATCACAAATG	C	8898.9	GAAATAAATCTTTAAATGATCACAAATGG	A	8938.8	GAAATAAATCTTTAAATGATCACAAATGT			

Assay definitions for the Agena iPLEX Mass-Array genotyping platform for the 129 SNPs investigated in this study. The designs were undertaken using the MassARRAY® Assay Design 3.1 software, which grouped the assays into 4 multiplexes assigned W1 - W4 (WELL column). Field definitions are as per the Agena assay design software users' guide (MassARRAY® Assay Design 3.1, Sequenom Inc., San Diego, Ca, USA).

Table S2: Assay definitions for the Agena iPLEX genotyping platform.

Gene	SNPID	Alleles (ancestral / derived)	Chr	DAF Controls	No. of genotype in controls	Sample	Case Phenotype	DAF Cases	No. of genotypes in cases	MO	Unadjusted§				Adjusted§			
											OR	LCL	UCL	P	OR	LCL	UCL	P
AJAP1	rs113788643	A/G	1	0.08	3940	All	SM	0.07	2215	A	0.95	0.82	1.09	0.4382	0.92	0.79	1.06	0.4900
AJAP1	rs6674631	G/A	1	0.13	3928	All	SM	0.12	2212	A	0.89	0.79	1.00	0.0434	0.88	0.78	1.00	0.04
ATP2B4	rs10900585	G/T	1	0.34	3762	All	SM	0.33	2200	R	1.29	1.07	1.56	0.0056	1.23	1.10	1.81	0.0090
ATP2B4	rs1541255	A/G	1	0.33	3933	All	SM	0.31	2211	R	0.74	0.62	0.89	0.0012	0.76	0.63	0.92	0.001
ATP2B4	rs3753036	G/A	1	0.04	3939	All	SM	0.04	2217	R	1.60	0.42	6.07	0.4940	1.25	0.28	5.49	0.8400
ATP2B4	rs4951074	G/A	1	0.32	3934	All	SM	0.31	2215	R	0.77	0.64	0.93	0.0068	0.77	0.65	0.94	0.007
ATP2B4	rs55868763	G/C	1	0.33	3931	All	SM	0.31	2209	R	0.74	0.62	0.89	0.0013	0.75	0.61	0.92	0.0010
CR1	rs17047660	A/G	1	0.16	3923	All	SM	0.17	2228	R	1.24	0.90	1.72	0.1932	1.14	0.81	1.61	0.18
CR1	rs17047661	G/A	1	0.32	3934	All	SM	0.33	2223	A	1.07	0.98	1.15	0.1249	1.06	0.98	1.16	0.1300
DARC	rs2814778	G/A	1	0.00	3944	All	SM	0.00	1933	H	0.83	0.33	2.10	0.6934	0.77	0.30	1.97	0.69
GBP7	rs1803632	G/C	1	0.49	3942	All	SM	0.51	2230	R	1.11	0.98	1.25	0.0940	1.10	0.96	1.25	0.1200
IL10	rs1800890	A/T	1	0.24	3945	All	SM	0.23	2233	R	0.69	0.55	0.88	0.0025	0.72	0.56	0.93	0.002
IL10	rs1800896	T/C	1	0.38	3943	All	SM	0.36	2225	R	0.90	0.77	1.05	0.1961	0.91	0.77	1,07	0.2200
IL10	rs3024500	G/A	1	0.45	3928	All	SM	0.47	2226	R	1.18	1.04	1.34	0.0129	1.17	1.02	1.33	0.01
LPHN2	rs146428334	A/G	1	0.00	3930	All	SM	0.00	2216	H	1.29	0.74	2.23	0.3751	1.30	0.74	2.30	0.3600
LPHN2	rs4650365	T/C	1	0.20	3936	All	SM	0.19	2215	H	0.85	0.76	0.95	0.0050	0.86	0.76	0.97	0.003
LPHN2	rs72933304	C/A	1	0.09	3942	All	SM	0.07	2216	A	0.80	0.69	0.92	0.0019	0.83	0.72	0.96	0.0020
LPHN2	rs72933310	T/C	1	0.09	3927	All	SM	0.08	2214	A	0.85	0.74	0.97	0.0158	0.87	0.75	0.98	0.01
LPHN2	rs72933350	T/C	1	0.05	3940	All	SM	0.04	2215	D	0.75	0.62	0.92	0.0037	0.82	0.67	0.94	0.0040
IL1A	rs17561	G/T	2	0.16	3938	All	SM	0.18	2232	R	1.47	1.08	2.00	0.0152	1.49	1.08	2.05	0.01
IL1B	rs1143634	C/T	2	0.11	3941	All	SM	0.13	2229	A	1.08	0.96	1.21	0.1850	1.06	0.94	1.19	0.2300
LAPTM4A	rs973128	C/G	2	0.44	3885	All	SM	0.44	2183	H	1.03	0.92	1.14	0.6460	0.98	0.87	1.11	0.68
LOC727982	rs10188961	A/G	2	0.40	3934	All	SM	0.42	2211	D	1.21	1.08	1.35	0.0009	1.14	1.05	1.24	0.0007
LOC727982	rs1371474	T/C	2	0.27	3933	All	SM	0.29	2216	H	1.29	1.16	1.43	4.19×10^{-6}	1.34	1.19	1.50	3.86×10^{-6}
LOC727982	rs1371478	C/T	2	0.27	3909	All	SM	0.29	2209	H	1.30	1.17	1.45	1.90×10^{-6}	1.35	1.20	1.51	2.31×10^{-6}
SDC1	rs11899121	C/G	2	0.47	3921	All	SM	0.47	2210	H	1.06	0.96	1.18	0.2570	1.06	0.95	1.19	0.23
ZSWIM2	rs144778284	C/T	2	0.00	3937	All	SM	0.00	2216	R	-	-	-	-	-	-	-	-
ZSWIM2	rs4316902	A/G	2	0.18	3910	All	SM	0.17	2205	R	0.79	0.57	1.08	0.1344	0.81	0.58	1.14	0.12
ARL14	rs74954675	A/C	3	0.08	3907	All	SM	0.10	2200	H	1.24	1.07	1.43	0.0039	1.22	1.05	1.42	0.0030
ARL14	rs75731597	A/C	3	0.08	3491	All	SM	0.10	2173	H	1.27	1.10	1.47	0.0013	1.25	1.07	1.46	0.001
ARL14	rs76033371	A/G	3	0.09	3939	All	SM	0.10	2207	H	1.22	1.06	1.40	0.0060	1.19	1.03	1.38	0.0060
B3GALNT1	rs12107243	G/C	3	0.49	3934	All	SM	0.49	2213	D	0.95	0.84	1.08	0.4393	0.97	0.85	1.10	0.45
IL17RD	rs6780995	A/G	3	0.41	3939	All	SM	0.40	2230	R	0.90	0.78	1.03	0.1283	0.91	0.79	1.06	0.1700
IL17RE	rs708567	A/G	3	0.50	3881	All	SM	0.50	2221	R	0.97	0.86	1.10	0.6779	0.98	0.86	1.12	0.66
TLR9	rs187084	T/C	3	0.33	3933	All	SM	0.32	1937	A	0.95	0.88	1.04	0.2620	0.92	0.84	1.00	0.2200
FREM3	rs149914432	A/C	4	0.10	3915	All	SM	0.06	2202	A	0.60	0.51	0.69	8.57×10^{-13}	0.64	0.53	0.78	6.20×10^{-13}
FREM3	rs184895969	C/T	4	0.00	3940	All	SM	0.00	2217	H	0.83	0.45	1.53	0.5508	1.00	0.51	1.94	0.5600
FREM3	rs184908374	G/A	4	0.00	3943	All	SM	0.00	2217	H	0.95	0.50	1.82	0.8842	1.14	0.56	2.30	0.9
FREM3	rs186790584	A/T	4	0.10	3941	All	SM	0.06	2217	A	0.59	0.50	0.68	2.71×10^{-13}	0.60	0.51	0.7	2.24×10^{-13}
FREM3	rs186873296	A/G	4	0.10	3926	All	SM	0.06	2206	A	0.57	0.49	0.66	4.23×10^{-14}	0.64	0.53	0.79	3.18×10^{-14}
GAB1	rs7663712	A/G	4	0.02	3938	All	SM	0.03	2216	H	1.09	0.86	1.39	0.4862	1.04	0.81	1.31	0.4830
INPP4B	rs13103597	C/T	4	0.27	3925	All	SM	0.23	2215	A	0.82	0.75	0.89	7.41×10^{-6}	0.89	0.80	0.98	6.29×10^{-6}
INPP4B	rs77389579	G/T	4	0.07	3937	All	SM	0.04	2209	A	0.61	0.51	0.72	4.48×10^{-9}	0.64	0.53	0.77	4.79×10^{-9}
TLR1	rs4833095	C/T	4	0.09	3921	All	SM	0.10	1936	R	1.49	0.87	2.57	0.1519	1.26	0.70	2.26	0.15
TLR6	rs5743809	T/C	4	0.06	3884	All	SM	0.06	1847	R	1.41	0.62	3.21	0.4159	1.52	0.65	3.56	0.4120
TLR6	rs5743810	C/T	4	0.02	3936	All	SM	0.01	1940	R	-	-	-	-	-	-	-	-
USP38	rs28459062	T/C	4	0.16	3931	All	SM	0.18	2217	A	1.14	1.03	1.26	0.0095	1.10	1.10	1.27	0.0100
USP38	rs4266246	C/T	4	0.22	3928	All	SM	0.22	2212	R	1.09	0.86	1.40	0.4724	1.02	0.79	1.31	0.416
C6	rs1801033	A/C	5	0.47	3927	All	SM	0.47	1933	A	0.99	0.92	1.07	0.8171	0.99	0.91	1.07	0.8230
IL13	rs20541	C/T	5	0.22	3796	All	SM	0.21	1850	R	0.88	0.67	1.14	0.3288	0.86	0.65	1.14	0.39
IRF1	rs2706384	C/A	5	0.38	3819	All	SM	0.36	1886	A	0.95	0.87	1.03	0.1835	0.94	0.86	1.02	0.2140
CTL4	rs2242665	A/G	6	0.26	3853	All	SM	0.27	1911	R	1.16	0.94	1.43	0.1737	1.15	0.92	1.43	0.17
GABBR1	rs192151845	G/A	6	0.01	3943	All	SM	0.01	2212	H	1.07	0.69	1.66	0.7515	1.07	0.68	1.69	0.8300
HCG4	rs114980857	C/T	6	0.21	3843	All	SM	0.22	2188	H	1.04	0.93	1.17	0.4713	1.01	0.89	1.14	0.495
HSPA1B	rs6457452	C/T	6	0.19	3917	All	SM	0.18	2212	D	0.88	0.79	0.98	0.0243	0.88	0.78	0.99	0.0200
IL20RA	rs1555498	C/T	6	0.46	3947	All	SM	0.49	2228	A	1.05	0.98	1.13	0.1810	1.06	0.98	1.14	0.179
LTA	rs2239704	G/T	6	0.12	3896	All	SM	0.13	2187	D	1.07	0.95	1.21	0.2709	1.03	0.90	1.18	0.3110
LTA	rs909253	T/C	6	0.49	3921	All	SM	0.49	2216	H	1.03	0.92	1.14	0.6090	1.06	0.95	1.19	0.501
PLEKHG1	rs114726617	G/A	6	0.04	3940	All	SM	0.04	2217	A	1.15	0.95	1.39	0.1474	1.20	0.98	1.47	0.1720
PLEKHG1	rs141555199	C/G	6	0.02	3943	All	SM	0.02	2218	R	0.00	0.00	-	0.1424	-	-	-	-
PLEKHG1	rs142712208	G/A	6	0.03	3927	All	SM	0.04	2218	A	1.18	0.96	1.44	0.1147	1.27	1.02	1.57	0.1160
PLEKHG1	rs15116938	C/T	6	0.03	3939	All	SM	0.04	2216	A	1.13	0.93	1.39	0.2207	1.23	0.99	1.52	0.225
PLEKHG1	rs151293197	C/G	6	0.03	3940	All	SM	0.04	2217	A	1.12	0.91	1.37	0.2790	1.19	0.96	1.48	0.2850
PLEKHG1	rs2131263	C/T	6	0.04	3938	All	SM	0.04	2216	A	1.14	0.95	1.38	0.1629	1.20	0.98	1.46	0.172
PLEKHG1	rs55958968	A/G	6	0.05	3932	All	SM	0.06	2217	H	1.20	1.01	1.43	0.0376	1.21	1.01	1.45	0.0300
PLEKHG1	rs76924464	G/A	6	0.03	3940	All	SM	0.04	2217	A	1.12	0.92	1.37	0.2673	1.20	0.97	1.48	0.273
PLEKHG1	rs79100774	G/C	6	0.03	3939	All	SM	0.04	2217	A	1.11	0.91	1.36	0.2934	1.20	0.97	1.48	0.2980
SNORD48	rs116288147	C/T	6	0.24	3896	All	SM	0.23	2191	D	0.93	0.83	1.03	0.1621	0.92	0.82	1.04	0.138
TNF	rs1799964	T/C	6	0.27	3938	All	SM	0.27	2204	H	0.95	0.86	1.06	0.3902	0.94	0.83	1.05	0.4240
TNF	rs1800629	G/A	6	0.09	3940	All	SM	0.09	2230	H	0.92	0.80	1.06	0.2422	0.92	0.80	1.07	0.286
TNF	rs1800750	G/A	6	0.05	3868	All	SM	0.05	1281	A	0.94	0.77	1.16	0.5740	0.93	0.74	1.17	0.5100
TNF	rs3093662	A/G	6	0.11	3936	All	SM	0.11	2219	R	0.75	0.45	1.23	0.2424	0.74	0.45	1.23	0.247
TNF	rs361525	G/A	6	0.07	3948	All	SM	0.07	2224	R	0.66	0.29	1.49	0.3048	0.74	0.32	1.71	0.3080
CD36	rs3211938	T/G	7	0.10	3942	All	SM	0.09	2223	R	1.11	0.66	1.85	0.6936	1.13	0.65	1.93	0.685
NOD1	rs2075820	G/A	7	0.38	3941	All	SM	0.38	1940	H	1.04	0.93	1.16	0.5074	1.05	0.93	1.17	0.5400
ABO	rs56390333	G/A	9	0.00	3942	All	SM	0.00	2217	H	0.68	0.17	2.64	0.5641	0.76	0.19	3.06	0.571
ABO	rs8176719*	O vs non-O	9	0.26	3888	All	SM	0.31	2230	R	0.73	0.65	0.81	3.99×10^{-9}	0.74	0.66	0.82	6.26×10^{-8}
ABO	rs8176746*	C/A	9	0.13	3924	All	SM	0.17	2227	A	1.37	1.22	1.55	9.60×10^{-9}	1.38	1.22	1.50	2.38×10^{-7}
ABO	rs8176750	I/D	9	0.05	3923	All	SM	0.05	2211	R	0.61	0.19	1.94	0.3878	0.58	0.17	1.97	0.3940
TLR4	rs4986790	A/G	9	0.04	3929	All	SM	0.04	1926	R	0.17	0.02	1.29	0.0282	0.16	0.02	1.28	0.028
TLR4	rs4986791	C/T	9	0.01	3937	All	SM	0.01	1930	H	1.09	0.67	1.78	0.7198	1.14	0.68	1.89	0.7150

MKI67	rs148494166	T/C	10	0.00	3913	All	SM	0.00	2137	H	1.36	0.63	2.91	0.4401	1.16	0.53	2.56	0.427
MKI67	rs11016116	A/G	10	0.02	3944	All	SM	0.02	2218	R	1.65	0.41	6.68	0.4877	1.84	0.42	8.00	0.4810
MKI67	rs115947774	A/G	10	0.00	3931	All	SM	0.00	2206	H	0.49	0.23	1.06	0.0564	0.48	0.22	1.05	0.05
HBB	rs334	A/T	11	0.08	3949	All	SM	0.02	2227	H	0.14	0.12	0.21	6.81×10^{-59}	0.15	0.11	0.20	2.61×10^{-58}
RRM1	rs116472045	A/G	11	0.02	3939	All	SM	0.02	2213	R	-	-	-	-	-	-	-	-
RTN3	rs542998	T/C	11	0.43	3929	All	SM	0.44	1911	D	1.06	0.94	1.20	0.3118	1.09	0.96	1.23	0.2660
TRIM5	rs7935564	G/A	11	0.44	3871	All	SM	0.46	1930	A	1.08	1.00	1.17	0.0473	1.00	0.92	1.09	0.044
CAND1	rs10459266	C/A	12	0.21	3938	All	SM	0.22	2217	D	1.14	1.02	1.27	0.0179	1.13	1.01	1.27	0.0130
CAND1	rs12307123	C/T	12	0.23	3933	All	SM	0.24	2216	A	1.09	1.00	1.19	0.0566	1.11	0.99	1.24	0.04
CAND1	rs1566830	G/T	12	0.26	3940	All	SM	0.28	2215	A	1.11	1.02	1.20	0.0188	1.09	1.00	1.20	0.0150
GRIP1	rs1394263	T/C	12	0.26	3914	All	SM	0.28	2205	A	1.11	1.02	1.21	0.0150	1.10	1.01	1.20	0.012
IL22	rs1012356	A/T	12	0.49	3940	All	SM	0.48	2225	H	1.08	0.97	1.20	0.1519	1.07	0.96	1.20	0.1480
IL22	rs2227478	A/G	12	0.38	3939	All	SM	0.39	2230	R	0.97	0.84	1.13	0.7107	0.95	0.81	1.11	0.688
IL22	rs2227485	G/A	12	0.42	3928	All	SM	0.41	2221	R	0.86	0.75	0.99	0.0402	0.84	0.72	0.97	0.0370
IL22	rs2227491	C/T	12	0.40	3870	All	SM	0.42	2192	D	1.09	0.98	1.22	0.1196	1.07	0.95	1.20	0.1
IL22	rs2227507	T/C	12	0.04	3948	All	SM	0.04	2235	A	0.96	0.80	1.16	0.6840	0.96	0.79	1.17	0.7360
TPTE2	rs182873742	C/G	13	0.00	3940	All	SM	0.00	2202	H	4.92	0.50	48.34	0.1362	4.92	0.40	60.10	0.133
RPS6KL1	rs3742785	C/A	14	0.29	3714	All	SM	0.30	2115	H	1.20	1.08	1.34	0.0010	1.18	1.05	1.33	0.0016
SPTB	rs229587	T/C	14	0.34	3872	All	SM	0.34	2167	A	1.02	0.94	1.10	0.6677	1.01	0.93	1.09	0.637
YLPM1	rs10139016	C/T	14	0.25	3937	All	SM	0.27	2214	D	1.12	1.01	1.25	0.0313	1.11	0.99	1.24	0.0300
ADCY9	rs10775349	C/G	16	0.20	3941	All	SM	0.20	1937	R	1.09	0.82	1.44	0.5625	1.11	0.83	1.50	0.614
ADCY9	rs2230739	A/G	16	0.09	3944	All	SM	0.08	1940	H	0.87	0.74	1.01	0.0689	0.83	0.70	0.97	0.0500
HBA	$\alpha^{-3.7}$-thalassaemia	I/D	16	0.41	3946	All	SM	0.36	2143	A	0.82	0.76	0.89	6.59×10^{-7}	0.83	0.76	0.90	2.06×10^{-6}
IL4	rs2243250	T/C	16	0.20	3902	All	SM	0.22	2205	H	1.11	0.99	1.24	0.0632	1.09	0.97	1.23	0.0500
IL4R	rs1805015	T/C	16	0.42	3927	All	SM	0.43	2228	H	1.05	0.94	1.17	0.3772	1.05	0.94	1.18	0.368
ADORA2B	rs2535611	T/C	17	0.10	3911	All	SM	0.11	1935	A	1.08	0.96	1.23	0.2005	1.06	0.93	1.21	0.2020
NOS2	rs1800482	G/C	17	0.08	3922	All	SM	0.07	2220	A	0.97	0.84	1.12	0.6718	0.95	0.82	1.10	0.581
NOS2	rs2297518	G/A	17	0.14	3910	All	SM	0.13	1924	D	0.95	0.84	1.08	0.4227	0.96	0.84	1.10	0.4430
NOS2	rs8078340	C/T	17	0.21	3948	All	SM	0.21	2233	R	0.82	0.62	1.09	0.1624	0.78	0.58	1.04	0.146
NOS2	rs9282799	C/T	17	0.03	3949	All	SM	0.03	2224	H	1.14	0.91	1.44	0.2515	1.13	0.88	1.44	0.2990
TBX2	rs73991577	A/G	17	0.00	3937	All	SM	0.00	2215	H	0.48	0.19	1.20	0.0976	0.52	0.20	1.34	0.1
EMR1	rs373533	G/T	19	0.46	3863	All	SM	0.49	1927	A	1.09	1.01	1.18	0.0345	1.07	0.99	1.17	0.0400
EMR1	rs461645	C/T	19	0.47	3933	All	SM	0.49	1939	A	1.09	1.00	1.17	0.0374	1.07	0.99	1.16	0.048
ICAM	rs5498	A/G	19	0.11	3908	All	SM	0.12	2197	H	1.05	0.93	1.20	0.4327	1.03	0.89	1.18	0.4550
GNAS	rs8386	C/T	20	0.19	3944	All	SM	0.19	2229	H	0.91	0.81	1.02	0.1060	0.92	0.82	1.04	0.11
DERL3	rs1128127	G/A	22	0.50	3911	All	SM	0.47	2226	A	0.94	0.88	1.02	0.1241	0.93	0.86	1.01	0.1250
CD40LG	rs1126535	T/C	X	0.15	3934	M+F	SM	0.16	2230	H	0.86	0.72	1.02	0.0843	0.85	0.71	1.03	0.09
CD40LG	rs1126535	T/C	X	0.16	1947	F	SM	0.14	1080	A	0.85	0.73	0.99	0.0325	0.87	0.76	1.00	0.0425
CD40LG	rs1126535	C/T	X	0.85	1987	M	SM	0.83	1150	HM	0.84	0.69	1.03	0.0903	0.85	0.70	1.04	0.100257
CD40LG	rs3092945	T/C	X	0.21	3927	M+F	SM	0.26	2226	A	1.14	1.07	1.23	0.0002	1.15	1.06	1.23	0.0002
CD40LG	rs3092945	T/C	X	0.21	1942	F	SM	0.25	1082	A	1.25	1.10	1.42	0.0004	1.28	1.13	1.45	0.0002
CD40LG	rs3092945	C/T	X	0.78	1985	M	SM	0.74	1144	HM	0.83	0.70	0.99	0.0338	0.80	0.68	0.95	0.0100
G6PD	rs1050828	C/T	X	0.19	3940	M+F	SM	0.19	2220	H	0.82	0.69	0.96	0.0153	0.80	0.68	0.96	0.015
G6PD	rs1050828	C/T	X	0.20	1951	F	SM	0.18	1074	H	0.82	0.70	0.97	0.0100	0.81	0.69	0.96	0.0100
G6PD	rs1050828	C/T	X	0.19	1989	M	SM	0.20	1146	HM	1.11	0.92	1.34	0.2686	1.08	0.90	1.30	0.36
G6PD	rs1050829	T/C	X	0.40	3928	M+F	SM	0.42	2221	H	0.85	0.73	0.99	0.0342	0.86	0.73	1.01	0.0540
G6PD	rs1050829	T/C	X	0.41	1952	F	SM	0.41	1075	H	0.85	0.73	0.99	0.0358	0.86	0.74	1.00	0.04581
G6PD	rs1050829	C/T	X	0.60	1976	M	SM	0.58	1146	HM	0.90	0.77	1.04	0.1486	0.93	0.79	1.05	0.1526

* The rs8176719 polymorphism identifies blood-group O in the homozygous deletion form. The derived allele of rs8176746 identifies blood-group B. Chr = chromosome; DAF = derived allele frequency; MO = genotype model [A – additive, D – dominant, H – heterozygote, HM – hemizygous males, R – recessive] with respect to the derived allele; OR = Odds Ratio; LCI = Lower Confidence Interval (95%); UCI = Upper Confidence Interval; P = p-value using a logistic regression model as described in methods. § ORs and p-values unadjusted and adjusted for ethnic group, gender and rs334 genotype.

Table S3: Odds ratios for all SM for all polymorphisms investigated.

Gene	SNPID	Alleles (ancestral/ derived)	Chr	DAF Controls	No. of genotype in controls	Sample	Case Phenotype	DAF Cases	No. of genotypes in cases	MO	Unadjusted				Adjusted§			
											OR	LCL	UCL	P	OR	LCL	UCL	P
AJAP1	rs113788643	A/G	1	0.08	3940	All	CM	0.07	1231	H	0.94	0.77	1.13	0.5004	0.92	0.76	1.12	0.5601
AJAP1	rs6674631	G/A	1	0.13	3928	All	CM	0.11	1225	A	0.83	0.72	0.96	0.0100	0.83	0.71	0.96	0.01161
ATP2B4	rs10900585	G/T	1	0.34	3762	All	CM	0.32	1223	R	1.21	1.06	1.54	0.0210	1.39	0.89	2.18	0.0187
ATP2B4	rs1541255	A/G	1	0.33	3933	All	CM	0.30	1226	R	0.68	0.54	0.86	0.0011	0.69	0.54	0.89	0.0011
ATP2B4	rs3753036	G/A	1	0.04	3939	All	CM	0.04	1229	R	2.16	0.50	9.27	0.3208	1.51	0.28	8.19	0.7274
ATP2B4	rs4951074	G/A	1	0.32	3934	All	CM	0.30	1229	R	0.73	0.58	0.93	0.0092	0.97	0.60	1.55	0.0084671
ATP2B4	rs55868763	G/C	1	0.33	3931	All	CM	0.30	1227	R	0.68	0.54	0.87	0.0011	0.67	0.52	0.86	0.0009
CR1	rs17047660	A/G	1	0.16	3923	All	CM	0.18	1221	A	1.14	1.01	1.29	0.0397	1.11	0.98	1.26	0.058625
CR1	rs17047661	G/A	1	0.32	3934	All	CM	0.34	1217	A	1.10	1.00	1.21	0.0614	1.12	1.01	1.24	0.0732
DARC	rs2814778	G/A	1	0.00	3944	All	CM	0.00	1093	H	0.80	0.26	2.49	0.6942	0.74	0.24	2.34	0.69406
GBP7	rs1803632	G/C	1	0.49	3942	All	CM	0.51	1223	A	1.07	0.98	1.17	0.1484	1.08	0.98	1.18	0.1564
IL10	rs1800890	A/T	1	0.24	3945	All	CM	0.23	1225	R	0.74	0.55	0.99	0.0387	0.76	0.55	0.99	0.040069
IL10	rs1800896	T/C	1	0.38	3943	All	CM	0.36	1221	R	0.87	0.72	1.06	0.1673	0.87	0.71	1.06	0.1683
IL10	rs3024500	G/A	1	0.45	3928	All	CM	0.49	1222	R	1.26	1.08	1.47	0.0041	1.24	1.06	1.46	0.0083114
LPHN2	rs146428334	A/G	1	0.00	3930	All	CM	0.01	1230	H	1.51	0.80	2.86	0.2188	1.51	0.79	2.90	0.2102
LPHN2	rs4650365	T/C	1	0.20	3936	All	CM	0.19	1227	H	0.83	0.72	0.96	0.0091	0.84	0.72	0.97	0.0064098
LPHN2	rs72933304	C/A	1	0.09	3942	All	CM	0.07	1230	A	0.82	0.69	0.98	0.0279	0.86	0.71	0.99	0.0371
LPHN2	rs72933310	T/C	1	0.09	3927	All	CM	0.08	1229	D	0.86	0.72	1.03	0.1014	0.90	0.75	1.08	0.10928
LPHN2	rs72933350	T/C	1	0.05	3940	All	CM	0.04	1229	D	0.76	0.60	0.96	0.0203	0.82	0.64	1.05	0.0245
IL1A	rs17561	G/T	2	0.16	3938	All	CM	0.18	1224	R	1.40	0.96	2.04	0.0860	1.43	0.97	2.11	0.084987
IL1B	rs1143634	C/T	2	0.11	3941	All	CM	0.12	1221	H	1.07	0.91	1.25	0.4032	1.06	0.90	1.25	0.4466
LAPTM4A	rs973128	C/G	2	0.44	3885	All	CM	0.45	1214	D	1.08	0.93	1.24	0.3029	1.02	0.88	1.18	0.28849
LOC727982	rs10188961	A/G	2	0.40	3934	All	CM	0.41	1225	D	1.14	1.00	1.31	0.0551	1.17	1.01	1.35	0.0526
LOC727982	rs1371474	T/C	2	0.27	3933	All	CM	0.29	1230	H	1.32	1.16	1.50	4.21×10^{-5}	1.37	1.20	1.58	5.97×10^{-5}
LOC727982	rs1371478	C/T	2	0.27	3909	All	CM	0.29	1226	H	1.31	1.15	1.50	4.61×10^{-5}	1.37	1.19	1.57	7.19×10^{-5}
SDC1	rs11899121	C/G	2	0.47	3921	All	CM	0.48	1227	H	1.08	0.95	1.23	0.2659	1.06	0.93	1.21	0.22618
ZSWIM2	rs144778284	C/T	2	0.00	3937	All	CM	0.00	1229	R	-	-	-	-	-	-	-	-
ZSWIM2	rs4316902	A/G	2	0.18	3910	All	CM	0.17	1225	R	0.71	0.47	1.08	0.0984	0.76	0.50	1.17	0.10588
ARL14	rs74954675	A/C	3	0.08	3907	All	CM	0.09	1219	H	1.23	1.03	1.46	0.0227	1.24	1.03	1.49	0.0167
ARL14	rs75731597	A/C	3	0.08	3491	All	CM	0.10	1206	H	1.25	1.05	1.49	0.0123	1.25	1.04	1.51	0.010905
ARL14	rs76033371	A/G	3	0.09	3939	All	CM	0.10	1222	H	1.18	1.00	1.40	0.0529	1.19	1.00	1.42	0.0452
B3GALNT1	rs12107243	G/C	3	0.49	3934	All	CM	0.48	1227	D	0.91	0.79	1.06	0.2386	0.93	0.80	1.09	0.23785
IL17RD	rs6780995	A/G	3	0.41	3939	All	CM	0.39	1223	A	0.92	0.84	1.01	0.0763	0.93	0.84	1.02	0.0880
IL17RE	rs708567	A/G	3	0.50	3881	All	CM	0.49	1220	A	0.98	0.90	1.08	0.6937	0.95	0.81	1.10	0.65809
TLR9	rs187084	T/C	3	0.33	3933	All	CM	0.32	1095	A	0.92	0.83	1.02	0.0973	0.89	0.80	0.99	0.0894
FREM3	rs149914432	A/C	4	0.10	3915	All	CM	0.06	1221	A	0.60	0.50	0.73	2.12×10^{-8}	0.64	0.50	0.81	3.08×10^{-8}
FREM3	rs184895969	C/T	4	0.00	3940	All	CM	0.00	1230	H	0.73	0.33	1.60	0.4133	0.82	0.35	1.95	0.4217
FREM3	rs184908374	G/A	4	0.00	3943	All	CM	0.00	1230	H	0.90	0.40	2.01	0.7950	1.02	0.42	2.46	0.80742
FREM3	rs186790584	A/T	4	0.10	3941	All	CM	0.06	1229	A	0.60	0.50	0.72	1.44×10^{-8}	0.61	0.50	0.8	2.38×10^{-8}
FREM3	rs186873296	A/G	4	0.10	3926	All	CM	0.06	1225	A	0.59	0.49	0.71	7.16×10^{-9}	0.63	0.49	0.79	1.12×10^{-8}
GAB1	rs7663712	A/G	4	0.02	3938	All	CM	0.03	1229	A	1.18	0.89	1.57	0.2524	1.15	0.85	1.54	0.2709
INPP4B	rs13103597	C/T	4	0.27	3925	All	CM	0.23	1229	A	0.81	0.73	0.91	0.0002	0.88	0.78	0.98	0.00031243
INPP4B	rs77389579	G/T	4	0.07	3937	All	CM	0.05	1223	A	0.63	0.51	0.78	1.48×10^{-5}	0.66	0.52	0.83	1.64×10^{-5}
TLR1	rs4833095	C/T	4	0.09	3921	All	CM	0.10	1095	R	1.39	0.72	2.70	0.3349	1.35	0.69	2.67	0.33249
TLR6	rs5743809	T/C	4	0.06	3884	All	CM	0.06	1045	H	1.07	0.86	1.33	0.5686	0.73	0.20	2.60	0.6295
TLR6	rs5743810	C/T	4	0.02	3936	All	CM	0.01	1098	A	0.71	0.46	1.09	0.1017	0.62	0.39	0.97	0.10323
USP38	rs28459062	T/C	4	0.16	3931	All	CM	0.18	1230	A	1.16	1.03	1.31	0.0130	1.15	1.02	1.31	0.0169
USP38	rs4266246	C/T	4	0.22	3928	All	CM	0.22	1228	H	1.08	0.94	1.24	0.2589	1.03	0.89	1.19	0.23556
C6	rs1801033	A/C	5	0.47	3927	All	CM	0.48	1093	D	1.07	0.92	1.24	0.3939	1.06	0.91	1.24	0.3683
IL13	rs20541	C/T	5	0.22	3796	All	CM	0.21	1052	R	0.77	0.55	1.08	0.1217	0.75	0.53	1.06	0.14752
IRF	rs2706384	C/A	5	0.38	3819	All	CM	0.35	1072	D	0.90	0.78	1.04	0.1448	0.90	0.78	1.04	0.1709
CTL4	rs2242665	A/G	6	0.26	3853	All	CM	0.26	1077	H	1.02	0.89	1.18	0.7356	0.92	0.69	1.22	0.7311
GABBR1	rs192151845	G/A	6	0.01	3943	All	CM	0.01	1226	H	1.25	0.76	2.08	0.3858	1.31	0.78	2.19	0.3749
HCG4	rs114980857	C/T	6	0.21	3843	All	CM	0.22	1222	D	1.06	0.93	1.21	0.4043	1.04	0.91	1.20	0.40318
HSPA1B	rs6457452	C/T	6	0.19	3917	All	CM	0.18	1211	H	0.90	0.78	1.04	0.1480	0.92	0.79	1.06	0.1841
IL20RA	rs1555498	C/T	6	0.46	3947	All	CM	0.48	1222	R	1.03	0.88	1.20	0.7074	1.03	0.87	1.20	0.74783
LTA	rs2239704	G/T	6	0.12	3896	All	CM	0.13	1206	R	1.36	0.82	2.24	0.2373	1.58	0.94	2.67	0.2342
LTA	rs909253	T/C	6	0.49	3921	All	CM	0.49	1219	H	1.04	0.91	1.18	0.5970	1.06	0.93	1.22	0.5589
PLEKHG1	rs114726617	G/A	6	0.04	3940	All	CM	0.04	1230	A	1.20	0.95	1.50	0.1254	1.26	0.99	1.60	0.1354
PLEKHG1	rs141555199	C/G	6	0.02	3943	All	CM	0.02	1230	R	-	-	-	-	-	-	-	-
PLEKHG1	rs142712208	G/A	6	0.03	3927	All	CM	0.04	1231	D	1.21	0.94	1.57	0.1408	1.29	1.00	1.68	0.1658
PLEKHG1	rs15116938	C/T	6	0.03	3939	All	CM	0.04	1230	A	1.15	0.90	1.46	0.2823	1.23	0.95	1.59	0.32486
PLEKHG1	rs151293197	C/G	6	0.03	3940	All	CM	0.04	1230	A	1.16	0.90	1.48	0.2509	1.24	0.95	1.60	0.2907
PLEKHG1	rs2131263	C/T	6	0.04	3938	All	CM	0.04	1230	A	1.20	0.95	1.50	0.1261	1.26	0.99	1.60	0.13612
PLEKHG1	rs55958968	A/G	6	0.05	3932	All	CM	0.06	1230	H	1.17	0.94	1.46	0.1558	1.12	0.90	1.38	0.2841
PLEKHG1	rs76924464	G/A	6	0.03	3940	All	CM	0.04	1230	A	1.17	0.92	1.49	0.2128	1.25	0.97	1.62	0.24749
PLEKHG1	rs79100774	G/C	6	0.03	3939	All	CM	0.04	1230	A	1.17	0.92	1.49	0.2121	1.25	0.97	1.62	0.2448
SNORD48	rs116288147	C/T	6	0.24	3896	All	CM	0.24	1210	R	1.13	0.87	1.48	0.3661	1.15	0.86	1.53	0.49978
TNF	rs1799964	T/C	6	0.27	3938	All	CM	0.28	1210	R	1.07	0.84	1.36	0.5924	1.09	0.85	1.41	0.5856
TNF	rs1800629	G/A	6	0.09	3940	All	CM	0.09	1225	D	0.97	0.82	1.15	0.7156	0.97	0.82	1.16	0.76027
TNF	rs1800750	G/A	6	0.05	3868	All	CM	0.05	737	D	0.89	0.68	1.17	0.3967	0.90	0.68	1.19	0.3455
TNF	rs3093662	A/G	6	0.11	3936	All	CM	0.12	1219	R	0.70	0.37	1.32	0.2524	0.69	0.36	1.31	0.25352
TNF	rs361525	G/A	6	0.07	3948	All	CM	0.07	1220	R	0.75	0.28	2.00	0.5554	0.88	0.32	2.38	0.5562
CD36	rs3211938	T/G	7	0.10	3942	All	CM	0.09	1217	H	0.93	0.78	1.11	0.4026	0.93	0.77	1.12	0.39169
NOD1	rs2075820	G/A	7	0.38	3941	All	CM	0.39	1097	D	1.12	0.97	1.29	0.1114	1.11	0.96	1.29	0.1276
ABO	rs56390333	G/A	9	0.00	3942	All	CM	0.00	1231	H	1.21	0.31	4.75	0.7847	1.31	0.32	5.30	0.77807
ABO	rs8176719*	O vs non-O	9	0.26	3888	All	CM	0.30	1224	R	0.77	0.68	0.88	9.36×10^{-5}	0.78	0.69	0.92	0.0004
ABO	rs8176746*	C/A	9	0.13	3924	All	CM	0.16	1223	A	1.23	1.06	1.43	0.0005	1.22	1.04	1.42	0.01
ABO	rs8176750	I/D	9	0.05	3923	All	CM	0.05	1227	R	0.55	0.12	2.53	0.4175	0.56	0.12	2.73	0.4225
TLR4	rs4986790	A/G	9	0.04	3929	All	CM	0.04	1088	R	0.30	0.04	2.30	0.1705	0.29	0.04	2.28	0.17038
TLR4	rs4986791	C/T	9	0.01	3937	All	CM	0.01	1092	H	0.95	0.50	1.80	0.8704	0.97	0.50	1.89	0.8734
MKI67	rs11016116	A/G	10	0.02	3944	All	CM	0.03	1230	R	2.94	0.72	11.96	0.1403	3.34	0.77	14.58	0.13806
MKI67	rs115947774	A/G	10	0.00	3931	All	CM	0.00	1224	H	0.57	0.23	1.39	0.1912	0.57	0.23	1.41	0.1946

MKI67	rs148494166	T/C	10	0.00	3913	All	CM	0.00	1193	H	1.89	0.82	4.37	0.1512	1.59	0.67	3.74	0.14647
HBB	rs334	A/T	11	0.08	3949	All	CM	0.02	1222	H	0.14	0.09	0.21	8.64×10^{-39}	0.14	0.09	0.21	1.67×10^{-38}
RRM1	rs116472045	A/G	11	0.02	3939	All	CM	0.01	1227	A	0.66	0.43	1.03	0.0559	0.60	0.38	0.96	0.046517
RTN3	rs542998	T/C	11	0.43	3929	All	CM	0.42	1085	A	0.96	0.87	1.06	0.4499	0.99	0.82	1.19	0.4858
TRIM5	rs7935564	G/A	11	0.44	3871	All	CM	0.45	1093	R	1.12	0.94	1.33	0.2020	1.00	0.84	1.19	0.19199
CAND1	rs10459266	C/A	12	0.21	3938	All	CM	0.21	1229	A	1.05	0.94	1.18	0.3494	1.04	0.92	1.16	0.3012
CAND1	rs12307123	C/T	12	0.23	3933	All	CM	0.24	1230	D	1.16	1.02	1.33	0.0242	1.17	1.02	1.34	0.016717
CAND1	rs1566830	G/T	12	0.26	3940	All	CM	0.28	1229	A	1.13	1.02	1.26	0.0174	1.12	1.01	1.25	0.0132
GRIP1	rs1394263	T/C	12	0.26	3914	All	CM	0.28	1226	A	1.14	1.03	1.27	0.0116	1.13	1.02	1.26	0.0086331
IL22	rs1012356	A/T	12	0.49	3940	All	CM	0.48	1219	R	0.94	0.80	1.10	0.4187	0.96	0.82	1.13	0.4263
IL22	rs2227478	A/G	12	0.38	3939	All	CM	0.38	1224	R	0.91	0.76	1.09	0.3045	0.88	0.73	1.07	0.3128
IL22	rs2227485	G/A	12	0.42	3928	All	CM	0.41	1219	R	0.92	0.78	1.10	0.3618	0.89	0.74	1.07	0.3404
IL22	rs2227491	C/T	12	0.40	3870	All	CM	0.42	1200	A	1.06	0.96	1.16	0.2375	1.04	0.94	1.15	0.23557
IL22	rs2227507	T/C	12	0.04	3948	All	CM	0.04	1226	H	0.96	0.76	1.22	0.7470	0.97	0.76	1.24	0.7489
TPTE2	rs182873742	C/G	13	0.00	3940	All	CM	0.00	1222	H	3.67	0.22	61.54	0.3809	4.16	0.15	116.27	0.37748
RPS6KL1	rs3742785	C/A	14	0.29	3714	All	CM	0.29	1170	H	1.18	1.03	1.35	0.0143	1.19	1.03	1.37	0.0141
SPTB	rs229587	T/C	14	0.34	3872	All	CM	0.34	1192	R	0.91	0.74	1.12	0.3571	0.91	0.73	1.13	0.40718
YLPM1	rs10139016	C/T	14	0.25	3937	All	CM	0.27	1228	D	1.11	0.98	1.27	0.1059	1.10	0.96	1.26	0.1063
ADCY9	rs10775349	C/G	16	0.20	3941	All	CM	0.20	1097	H	1.05	0.91	1.22	0.4706	1.08	0.93	1.25	0.4308
ADCY9	rs2230739	A/G	16	0.09	3944	All	CM	0.07	1098	A	0.84	0.70	1.00	0.0503	0.83	0.69	1.00	0.0479
HBA	$\alpha^{-3.7}$-thalassaemia	I/D	16	0.41	3946	All	CM	0.37	1189	A	0.83	0.75	0.91	0.0001	0.83	0.76	0.90	0.0001
IL4	rs2243250	T/C	16	0.20	3902	All	CM	0.22	1212	D	1.13	0.98	1.29	0.0855	1.11	0.96	1.28	0.0858
IL4R	rs1805015	T/C	16	0.42	3927	All	CM	0.44	1223	D	1.12	0.98	1.29	0.1027	1.15	0.99	1.33	0.093058
ADORA2B	rs2535611	T/C	17	0.10	3911	All	CM	0.12	1094	D	1.10	0.93	1.30	0.2651	1.09	0.92	1.29	0.2820
NOS2	rs1800482	G/C	17	0.08	3922	All	CM	0.07	1218	H	0.94	0.78	1.14	0.5488	0.94	0.78	1.13	0.50249
NOS2	rs2297518	G/A	17	0.14	3910	All	CM	0.13	1091	A	0.92	0.80	1.06	0.2360	0.93	0.80	1.08	0.2342
NOS2	rs8078340	C/T	17	0.21	3948	All	CM	0.20	1225	R	0.66	0.45	0.97	0.0275	0.64	0.43	0.94	0.028385
NOS2	rs9282799	C/T	17	0.03	3949	All	CM	0.03	1220	H	1.29	0.98	1.69	0.0735	1.27	0.95	1.70	0.0796
TBX2	rs73991577	A/G	17	0.00	3937	All	CM	0.00	1230	H	0.57	0.19	1.70	0.2890	0.60	0.20	1.80	0.2945
EMR1	rs373533	G/T	19	0.46	3863	All	CM	0.49	1091	D	1.14	0.98	1.33	0.0908	1.12	0.95	1.32	0.1102
EMR1	rs461645	C/T	19	0.47	3933	All	CM	0.49	1097	D	1.14	0.98	1.33	0.0924	1.12	0.95	1.31	0.11209
ICAM	rs5498	A/G	19	0.11	3908	All	CM	0.13	1204	H	1.08	0.92	1.26	0.3494	1.05	0.89	1.24	0.3330
GNAS	rs8386	C/T	20	0.19	3944	All	CM	0.19	1223	R	1.46	1.05	2.03	0.0299	1.42	1.01	1.99	0.029177
DERL3	rs1128127	G/A	22	0.50	3911	All	CM	0.47	1221	R	0.92	0.79	1.08	0.3098	0.91	0.77	1.07	0.3245
CD40LG	rs1126535	T/C	X	0.15	3934	M+F	CM	0.17	1223	R	1.23	0.99	1.54	0.0649	1.20	0.95	1.51	0.089102
CD40LG	rs1126535	T/C	X	0.16	1947	F	CM	0.15	601	D	0.94	0.76	1.15	0.5486	0.95	0.77	1.16	0.5686
CD40LG	rs1126535	C/T	X	0.85	1987	M	CM	0.81	622	HM	0.77	0.61	0.98	0.0348	0.79	0.62	0.99	0.044779
CD40LG	rs3092945	T/C	X	0.21	3927	M+F	CM	0.25	1223	R	1.32	1.10	1.59	0.0034	1.38	1.14	1.67	0.0037
CD40LG	rs3092945	T/C	X	0.21	1942	F	CM	0.25	602	R	1.89	1.30	2.76	0.0012	1.23	1.05	1.43	0.007
CD40LG	rs3092945	C/T	X	0.78	1985	M	CM	0.74	621	HM	0.84	0.68	1.04	0.1097	0.81	0.66	1.00	0.0500
G6PD	rs1050828	C/T	X	0.19	3940	M+F	CM	0.18	1220	H	0.82	0.70	0.97	0.0100	0.80	0.68	0.96	0.0108
G6PD	rs1050828	C/T	X	0.20	1951	F	CM	0.18	600	H	0.80	0.65	0.98	0.0202	0.79	0.64	0.97	0.0200
G6PD	rs1050828	C/T	X	0.19	1989	M	CM	0.18	620	HM	0.92	0.72	1.17	0.4891	0.91	0.72	1.15	0.45
G6PD	rs1050829	T/C	X	0.40	3928	M+F	CM	0.41	1221	H	0.84	0.70	1.01	0.0609	0.87	0.71	1.06	0.0658
G6PD	rs1050829	T/C	X	0.41	1952	F	CM	0.41	601	H	0.84	0.70	1.01	0.0631	0.85	0.72	1.10	0.083127
G6PD	rs1050829	C/T	X	0.60	1976	M	CM	0.58	620	HM	0.94	0.78	1.14	0.5353	0.95	0.79	1.17	0.5353

* The rs8176719 polymorphism identifies blood-group O in the homozygous deletion form. The derived allele of rs8176746 identifies blood-group B. Chr = chromosome; DAF = derived allele frequency; MO = genotype model [A – additive, D – dominant, H – heterozygote, HM – hemizygous males, R – recessive] with respect to the derived allele; OR = Odds Ratio; LCI = Lower Confidence Interval (95%); UCI = Upper Confidence Interval; P = p-value using a logistic regression model as described in methods. § ORs and p-values unadjusted and adjusted for ethnic group, gender and rs334 genotype. All individuals having cerebral malaria were included in this analysis even if they were identified with multiple severe phenotypes.

Table S4: Odds ratios for all-CM for all polymorphisms investigated.

Gene	SNPID	Alleles (ancestral / derived)	Chr	DAF Controls	No. of genotype in controls	Sample	Case Phenotype	DAF Cases	No. of genotypes in cases	MO	Unadjusted				Adjusted[§]			
											OR	LCL	UCL	P	OR	LCL	UCL	P
AJAP1	rs113788643	A/G	1	0.08	3940	All	SMA	0.07	666	H	0.85	0.66	1.10	0.2075	0.82	0.63	1.07	0.2054
AJAP1	rs6674631	G/A	1	0.13	3928	All	SMA	0.11	667	A	0.84	0.70	1.00	0.0502	0.82	0.67	0.99	0.045017
ATP2B4	rs10900585	G/T	1	0.34	3762	All	SMA	0.31	659	R	1.52	1.11	2.09	0.0034	1.00	0.54	1.85	0.0040
ATP2B4	rs1541255	A/G	1	0.33	3933	All	SMA	0.29	665	R	0.63	0.46	0.87	0.0030	0.61	0.43	0.84	0.003432
ATP2B4	rs3753036	G/A	1	0.04	3939	All	SMA	0.05	667	D	1.21	0.91	1.60	0.1976	1.19	0.88	1.61	0.2236
ATP2B4	rs4951074	G/A	1	0.32	3934	All	SMA	0.29	666	R	0.66	0.48	0.91	0.0087	0.67	0.53	0.92	0.009973
ATP2B4	rs55868763	G/C	1	0.33	3931	All	SMA	0.29	661	R	0.62	0.45	0.85	0.0019	0.64	0.48	0.89	0.0021
CR1	rs17047660	A/G	1	0.16	3923	All	SMA	0.15	683	H	0.87	0.72	1.05	0.1335	0.85	0.69	1.03	0.096844
CR1	rs17047661	G/A	1	0.32	3934	All	SMA	0.33	685	A	1.06	0.93	1.20	0.3728	1.06	0.93	1.21	0.3946
DARC	rs2814778	G/A	1	0.00	3944	All	SMA	0.00	556	H	0.76	0.17	3.41	0.7084	0.74	0.16	3.35	0.71045
GBP7	rs1803632	G/C	1	0.49	3942	All	SMA	0.50	685	R	1.10	0.91	1.33	0.3224	1.10	0.90	1.34	0.4239
IL10	rs1800890	A/T	1	0.24	3945	All	SMA	0.24	687	R	0.83	0.58	1.19	0.3086	0.89	0.62	1.30	0.3312
IL10	rs1800896	T/C	1	0.38	3943	All	SMA	0.38	682	R	1.05	0.84	1.33	0.6612	1.08	0.84	1.37	0.5771
IL10	rs3024500	G/A	1	0.45	3928	All	SMA	0.47	682	H	0.80	0.68	0.95	0.0094	0.81	0.68	0.97	0.012456
LPHN2	rs146428334	A/G	1	0.00	3930	All	SMA	0.00	668	H	0.60	0.18	1.99	0.3751	0.58	0.17	1.93	0.3811
LPHN2	rs4650365	T/C	1	0.20	3936	All	SMA	0.18	667	D	0.85	0.71	1.01	0.0586	0.86	0.72	1.04	0.056572
LPHN2	rs72933304	C/A	1	0.09	3942	All	SMA	0.07	666	A	0.85	0.68	1.06	0.1517	0.88	0.70	1.11	0.1714
LPHN2	rs72933310	T/C	1	0.09	3927	All	SMA	0.08	664	A	0.90	0.73	1.12	0.3428	0.92	0.74	1.14	0.33259
LPHN2	rs72933350	T/C	1	0.05	3940	All	SMA	0.04	667	D	0.72	0.52	0.98	0.0303	0.79	0.57	1.10	0.0344
IL1A	rs17561	G/T	2	0.16	3938	All	SMA	0.17	687	R	1.05	0.63	1.76	0.8487	1.06	0.61	1.83	0.82324
IL1B	rs1143634	C/T	2	0.11	3941	All	SMA	0.13	686	A	1.10	0.92	1.30	0.3102	1.06	0.88	1.28	0.3764
LAPTM4A	rs973128	C/G	2	0.44	3885	All	SMA	0.46	658	A	1.11	0.98	1.25	0.0888	1.11	0.98	1.26	0.079476
LOC727982	rs10188961	A/G	2	0.40	3934	All	SMA	0.45	665	D	1.44	1.20	1.72	0.0001	1.48	1.22	1.80	0.00005
LOC727982	rs1371474	T/C	2	0.27	3933	All	SMA	0.30	667	H	1.32	1.11	1.55	0.0013	1.42	1.19	1.69	0.001172
LOC727982	rs1371478	C/T	2	0.27	3909	All	SMA	0.30	663	D	1.31	1.11	1.55	0.0014	1.45	1.22	1.74	0.0011
SDC1	rs11899121	C/G	2	0.47	3921	All	SMA	0.47	666	D	0.97	0.81	1.16	0.7102	1.01	0.90	1.15	0.91039
ZSWIM2	rs144778284	C/T	2	0.00	3937	All	SMA	0.00	667	R	-	-	-	-	-	-	-	-
ZSWIM2	rs4316902	A/G	2	0.18	3910	All	SMA	0.18	666	R	0.87	0.53	1.42	0.5709	0.88	0.52	1.48	0.58979
ARL14	rs74954675	A/C	3	0.08	3907	All	SMA	0.10	662	H	1.31	1.05	1.62	0.0192	1.27	1.00	1.60	0.0243
ARL14	rs75731597	A/C	3	0.08	3491	All	SMA	0.10	646	H	1.27	1.02	1.59	0.0353	1.30	1.07	1.59	0.046063
ARL14	rs76033371	A/G	3	0.09	3939	All	SMA	0.11	661	H	1.26	1.02	1.56	0.0353	1.20	0.95	1.50	0.0431
B3GALNT1	rs12107243	G/C	3	0.49	3934	All	SMA	0.49	667	D	1.02	0.84	1.23	0.8608	1.03	0.91	1.17	0.92778
IL17RD	rs6780995	A/G	3	0.41	3939	All	SMA	0.39	687	R	0.84	0.67	1.05	0.1222	0.87	0.68	1.10	0.1597
IL17RE	rs708567	A/G	3	0.50	3881	All	SMA	0.49	685	D	0.95	0.79	1.15	0.5998	0.94	0.77	1.15	0.54384
TLR9	rs187084	T/C	3	0.33	3933	All	SMA	0.33	557	H	0.95	0.79	1.14	0.5787	0.95	0.79	1.15	0.5583
FREM3	rs149914432	A/C	4	0.10	3915	All	SMA	0.06	663	A	0.58	0.45	0.74	2.26×10^{-6}	0.68	0.50	0.94	3.68×10^{-6}
FREM3	rs184895969	C/T	4	0.00	3940	All	SMA	0.00	668	H	1.02	0.42	2.47	0.9662	1.26	0.48	3.27	0.9530
FREM3	rs184908374	G/A	4	0.00	3943	All	SMA	0.00	666	H	1.25	0.51	3.07	0.6373	1.62	0.61	4.31	0.62615
FREM3	rs186790584	A/T	4	0.10	3941	All	SMA	0.06	667	A	0.57	0.44	0.73	1.55×10^{-6}	0.63	0.48	0.8	2.72×10^{-6}
FREM3	rs186873296	A/G	4	0.10	3926	All	SMA	0.05	663	A	0.55	0.43	0.70	4.56×10^{-7}	0.67	0.44	0.83	8.20×10^{-7}
GAB1	rs7663712	A/G	4	0.02	3938	All	SMA	0.03	668	R	-	-	-	-	1.25	0.85	1.82	0.3793
INPP4B	rs13103597	C/T	4	0.27	3925	All	SMA	0.22	666	D	0.76	0.64	0.90	0.0013	0.75	0.64	0.89	0.001257
INPP4B	rs77389579	G/T	4	0.07	3937	All	SMA	0.04	666	A	0.56	0.42	0.75	3.57×10^{-5}	0.60	0.43	0.82	5.35×10^{-5}
TLR1	rs4833095	C/T	4	0.09	3921	All	SMA	0.08	558	H	0.79	0.60	1.03	0.0697	0.84	0.66	1.07	0.44251
TLR6	rs5743809	T/C	4	0.06	3884	All	SMA	0.05	533	H	0.89	0.65	1.20	0.4320	0.89	0.64	1.24	0.3248
TLR6	rs5743810	C/T	4	0.02	3936	All	SMA	0.01	558	A	0.78	0.44	1.37	0.3732	0.72	0.40	1.28	0.37936
USP38	rs28459062	T/C	4	0.16	3931	All	SMA	0.17	666	A	1.07	0.92	1.25	0.3788	1.04	0.88	1.22	0.4579
USP38	rs4266246	C/T	4	0.22	3928	All	SMA	0.23	667	R	1.23	0.86	1.77	0.2740	1.14	0.78	1.66	0.25133
C6	rs1801033	A/C	5	0.47	3927	All	SMA	0.46	556	R	0.92	0.74	1.14	0.4372	0.89	0.71	1.12	0.4362
IL13	rs20541	C/T	5	0.22	3796	All	SMA	0.22	535	R	0.63	0.38	1.03	0.0520	0.59	0.35	1.00	0.062928
IRF	rs2706384	C/A	5	0.38	3819	All	SMA	0.37	546	H	1.25	1.04	1.49	0.0164	1.28	1.06	1.55	0.0102
CTL4	rs2242665	A/G	6	0.26	3853	All	SMA	0.25	550	H	0.86	0.71	1.03	0.1016	0.81	0.67	0.99	0.080986
GABBR1	rs192151845	G/A	6	0.01	3943	All	SMA	0.01	665	H	1.13	0.59	2.18	0.7198	1.15	0.59	2.25	0.7063
HCG4	rs114980857	C/T	6	0.21	3843	All	SMA	0.21	654	R	0.87	0.57	1.31	0.4867	0.81	0.52	1.26	0.50211
HSPA1B	rs6457452	C/T	6	0.19	3917	All	SMA	0.17	674	D	0.84	0.70	1.00	0.0478	0.81	0.66	0.99	0.0443
IL20RA	rs1555498	C/T	6	0.46	3947	All	SMA	0.50	684	R	1.16	0.96	1.40	0.1235	1.17	0.96	1.43	0.1376
LTA	rs2239704	G/T	6	0.12	3896	All	SMA	0.11	670	H	0.86	0.70	1.06	0.1431	0.81	0.65	1.01	0.1256
LTA	rs909253	T/C	6	0.49	3921	All	SMA	0.50	680	D	1.10	0.91	1.33	0.3397	1.22	0.99	1.49	0.25607
PLEKHG1	rs114726617	G/A	6	0.04	3940	All	SMA	0.04	668	R	2.32	0.60	8.93	0.2513	2.99	0.74	12.04	0.2482
PLEKHG1	rs141555199	C/G	6	0.02	3943	All	SMA	0.02	667	R	-	-	-	-	-	-	-	-
PLEKHG1	rs142712208	G/A	6	0.03	3927	All	SMA	0.04	667	R	3.51	0.87	14.27	0.1071	5.40	1.28	22.73	0.1055
PLEKHG1	rs15116938	C/T	6	0.03	3939	All	SMA	0.04	668	R	4.83	1.34	17.36	0.0270	7.43	1.99	27.71	0.026517
PLEKHG1	rs151293197	C/G	6	0.03	3940	All	SMA	0.04	667	R	3.52	0.87	14.30	0.1065	5.40	1.28	22.76	0.1049
PLEKHG1	rs2131263	C/T	6	0.04	3938	All	SMA	0.04	668	R	2.32	0.60	8.92	0.2515	2.99	0.74	12.04	0.24833
PLEKHG1	rs55958968	A/G	6	0.05	3932	All	SMA	0.06	668	H	1.21	0.93	1.58	0.1617	1.27	0.96	1.67	0.1399
PLEKHG1	rs76924464	G/A	6	0.03	3940	All	SMA	0.04	668	R	3.51	0.87	14.26	0.1071	5.37	1.28	22.62	0.10551
PLEKHG1	rs79100774	G/C	6	0.03	3939	All	SMA	0.04	665	R	3.53	0.87	14.32	0.1060	5.39	1.28	22.72	0.1044
SNORD48	rs116288147	C/T	6	0.24	3896	All	SMA	0.22	658	D	0.89	0.75	1.06	0.1831	0.86	0.72	1.03	0.17358
TNF	rs1799964	T/C	6	0.27	3938	All	SMA	0.28	674	R	1.10	0.81	1.49	0.5481	1.07	0.78	1.48	0.5174
TNF	rs1800629	G/A	6	0.09	3940	All	SMA	0.10	685	R	1.92	0.90	4.09	0.1097	1.84	0.81	4.15	0.19385
TNF	rs1800750	G/A	6	0.05	3868	All	SMA	0.05	423	R	0.53	0.07	4.01	0.5007	0.72	0.09	5.54	0.5090
TNF	rs3093662	A/G	6	0.11	3936	All	SMA	0.10	682	R	0.43	0.16	1.20	0.0698	0.45	0.16	1.26	0.072694
TNF	rs361525	G/A	6	0.07	3948	All	SMA	0.06	684	R	-	-	-	-	-	-	-	-
CD36	rs3211938	T/G	7	0.10	3942	All	SMA	0.09	684	A	0.91	0.75	1.12	0.3659	0.88	0.71	1.09	0.37691
NOD1	rs2075820	G/A	7	0.38	3941	All	SMA	0.37	557	H	1.07	0.89	1.28	0.4797	1.09	0.91	1.32	0.4949
ABO	rs56390333	G/A	9	0.00	3942	All	SMA	0.00	666	H	-	-	-	-	-	-	-	-
ABO	rs8176719*	O vs non-O	9	0.26	3888	All	SMA	0.32	686	R	0.66	0.56	0.78	8.45×10^{-7}	0.67	0.44	0.83	3.68×10^{-6}
ABO	rs8176746*	C/A	9	0.13	3924	All	SMA	0.18	682	A	1.44	1.23	1.73	8.50×10^{-5}	1.44	1.19	1.74	0.0001
ABO	rs8176750	I/D	9	0.05	3923	All	SMA	0.05	665	H	0.97	0.72	1.29	0.8100	0.76	0.55	1.05	0.7616
TLR4	rs4986790	A/G	9	0.04	3929	All	SMA	0.04	554	R	-	-	-	-	-	-	-	-
TLR4	rs4986791	C/T	9	0.01	3937	All	SMA	0.01	554	H	1.06	0.48	2.36	0.8889	1.07	0.46	2.51	0.8788
MKI67	rs11016116	A/G	10	0.02	3944	All	SMA	0.02	667	R	1.32	0.15	11.95	0.8091	1.52	0.15	15.27	0.80321
MKI67	rs115947774	A/G	10	0.00	3931	All	SMA	0.00	664	H	0.37	0.09	1.56	0.1190	0.41	0.09	1.75	0.1222

MKI67	rs148494166	T/C	10	0.00	3913	All	SMA	0.00	645	H	0.40	0.05	3.04	0.3102	0.33	0.04	2.54	0.31262
HBB	rs334	A/T	11	0.08	3949	All	SMA	0.02	683	H	0.07	0.04	0.15	2.11×10^{-31}	0.07	0.03	0.17	2.61×10^{-31}
RRM1	rs116472045	A/G	11	0.02	3939	All	SMA	0.02	666	R	-	-	-	-	-	-	-	-
RTN3	rs542998	T/C	11	0.43	3929	All	SMA	0.45	546	D	1.14	0.94	1.39	0.1843	1.15	0.94	1.41	0.1740
TRIM5	rs7935564	G/A	11	0.44	3871	All	SMA	0.47	554	D	1.24	1.01	1.51	0.0381	1.18	0.95	1.47	0.032002
CAND1	rs10459266	C/A	12	0.21	3938	All	SMA	0.23	667	D	1.22	1.03	1.44	0.0218	1.23	1.03	1.48	0.0210
CAND1	rs12307123	C/T	12	0.23	3933	All	SMA	0.24	666	D	1.14	0.97	1.35	0.1207	1.16	0.97	1.38	0.083643
CAND1	rs1566830	G/T	12	0.26	3940	All	SMA	0.28	666	A	1.13	0.99	1.29	0.0768	1.13	0.98	1.30	0.0594
GRIP1	rs1394263	T/C	12	0.26	3914	All	SMA	0.28	661	A	1.13	0.99	1.29	0.0741	1.13	0.99	1.30	0.057273
IL22	rs1012356	A/T	12	0.49	3940	All	SMA	0.49	685	H	1.18	1.00	1.39	0.0455	1.17	0.99	1.40	0.0560
IL22	rs2227478	A/G	12	0.38	3939	All	SMA	0.38	686	R	0.85	0.67	1.08	0.1800	0.87	0.68	1.12	0.20618
IL22	rs2227485	G/A	12	0.42	3928	All	SMA	0.41	681	H	1.27	1.08	1.50	0.0038	1.27	1.07	1.51	0.0060
IL22	rs2227491	C/T	12	0.40	3870	All	SMA	0.40	676	H	1.13	0.96	1.33	0.1469	0.82	0.64	1.06	0.17937
IL22	rs2227507	T/C	12	0.04	3948	All	SMA	0.04	687	A	0.83	0.61	1.12	0.2136	0.84	0.61	1.16	0.2545
TPTE2	rs182873742	C/G	13	0.00	3940	All	SMA	0.00	663	H	6.43	0.39	104.8	0.2186	5.95	0.26	135.3	0.21702
RPS6KL1	rs3742785	C/A	14	0.29	3714	All	SMA	0.32	650	D	1.33	1.12	1.57	0.0010	1.37	1.15	1.64	0.0010
SPTB	rs229587	T/C	14	0.34	3872	All	SMA	0.35	660	R	1.13	0.88	1.45	0.3452	1.07	0.81	1.39	0.33503
YLPM1	rs10139016	C/T	14	0.25	3937	All	SMA	0.28	666	D	1.25	1.06	1.47	0.0095	1.26	1.06	1.51	0.0104
ADCY9	rs10775349	C/G	16	0.20	3941	All	SMA	0.21	557	H	1.08	0.89	1.30	0.4300	1.06	0.87	1.30	0.40226
ADCY9	rs2230739	A/G	16	0.09	3944	All	SMA	0.08	558	R	0.49	0.12	2.07	0.2842	0.50	0.12	2.13	0.2861
HBA	$\alpha^{-3.7}$-thalassaemia	I/D	16	0.41	3946	All	SMA	0.34	639	A	0.72	0.64	0.82	1.55×10^{-6}	0.72	0.65	0.84	5.45×10^{-6}
IL4	rs2243250	T/C	16	0.20	3902	All	SMA	0.21	677	R	0.69	0.43	1.11	0.1067	1.02	0.87	1.19	0.6437
IL4R	rs1805015	T/C	16	0.42	3927	All	SMA	0.42	684	D	0.97	0.82	1.15	0.7331	1.03	0.86	1.24	0.64537
ADORA2B	rs2535611	T/C	17	0.10	3911	All	SMA	0.11	554	A	1.07	0.87	1.30	0.5378	1.01	0.81	1.25	0.5881
NOS2	rs1800482	G/C	17	0.08	3922	All	SMA	0.07	683	R	0.46	0.11	1.96	0.2429	0.22	0.04	1.40	0.24753
NOS2	rs2297518	G/A	17	0.14	3910	All	SMA	0.14	553	R	0.87	0.45	1.71	0.6893	0.98	0.50	1.93	0.7002
NOS2	rs8078340	C/T	17	0.21	3948	All	SMA	0.20	686	A	0.96	0.83	1.11	0.5666	0.93	0.80	1.08	0.42929
NOS2	rs9282799	C/T	17	0.03	3949	All	SMA	0.03	685	R	-	-	-	-	-	-	-	-
TBX2	rs73991577	A/G	17	0.00	3937	All	SMA	0.00	667	H	0.27	0.04	2.03	0.1199	0.33	0.04	2.54	0.12192
EMR1	rs373533	G/T	19	0.46	3863	All	SMA	0.49	551	D	1.14	0.93	1.40	0.2161	1.10	0.89	1.36	0.2489
EMR1	rs461645	C/T	19	0.47	3933	All	SMA	0.49	558	A	1.07	0.95	1.22	0.2667	1.06	0.93	1.21	0.24864
ICAM	rs5498	A/G	19	0.11	3908	All	SMA	0.12	677	H	1.07	0.88	1.31	0.4818	1.02	0.83	1.27	0.5481
GNAS	rs8386	C/T	20	0.19	3944	All	SMA	0.19	686	R	1.39	0.92	2.09	0.1278	1.35	0.88	2.07	0.11859
DERL3	rs1128127	G/A	22	0.50	3911	All	SMA	0.47	684	R	0.88	0.72	1.07	0.1880	0.84	0.68	1.04	0.2062
CD40LG	rs1126535	T/C	X	0.15	3934	M+F	SMA	0.16	686	H	0.75	0.56	0.99	0.0416	0.75	0.56	1.02	0.071258
CD40LG	rs1126535	T/C	X	0.16	1947	F	SMA	0.13	328	D	0.75	0.57	0.99	0.0393	0.77	0.61	0.99	0.0393
CD40LG	rs1126535	C/T	X	0.85	1987	M	SMA	0.81	358	HM	0.74	0.55	1.00	0.0528	0.76	0.58	1.10	0.056789
CD40LG	rs3092945	T/C	X	0.21	3927	M+F	SMA	0.26	683	A	1.17	1.05	1.31	0.0038	1.18	1.06	1.33	0.0031
CD40LG	rs3092945	T/C	X	0.21	1942	F	SMA	0.26	328	A	1.33	1.10	1.62	0.0037	1.36	1.12	1.64	0.001
CD40LG	rs3092945	C/T	X	0.78	1985	M	SMA	0.74	355	HM	0.81	0.63	1.05	0.1224	0.79	0.61	1.02	0.0700
G6PD	rs1050828	C/T	X	0.19	3940	M+F	SMA	0.23	680	R	1.60	1.26	2.03	0.0002	1.73	1.34	2.23	0.000171
G6PD	rs1050828	C/T	X	0.20	1951	F	SMA	0.17	325	H	0.79	0.61	1.03	0.0743	0.78	0.60	1.02	0.0600
G6PD	rs1050828	C/T	X	0.19	1989	M	SMA	0.28	355	HM	1.72	1.32	2.23	6.98×10^{-5}	1.68	1.30	2.17	0.0001
G6PD	rs1050829	T/C	X	0.40	3928	M+F	SMA	0.45	680	R	1.30	1.08	1.56	0.0054	1.16	1.03	1.31	0.0138
G6PD	rs1050829	T/C	X	0.41	1952	F	SMA	0.41	325	R	1.06	0.77	1.45	0.7268	1.09	0.79	1.55	0.72678
G6PD	rs1050829	C/T	X	0.60	1976	M	SMA	0.51	355	HM	0.69	0.55	0.87	0.0016	0.71	0.58	0.90	0.0017

* The rs8176719 polymorphism identifies blood-group O in the homozygous deletion form. The derived allele of rs8176746 identifies blood-group B. Chr = chromosome; DAF = derived allele frequency; MO = genotype model [A – additive, D – dominant, H – heterozygote, HM – hemizygous males, R – recessive] with respect to the derived allele; OR = Odds Ratio; LCI = Lower Confidence Interval (95%); UCI = Upper Confidence Interval; P = p-value using a logistic regression model as described in methods. § ORs and p-values unadjusted and adjusted for ethnic group, gender and rs334 genotype. All individuals having severe malarial anaemia were included in this analysis even if they were identified with multiple severe phenotypes.

Table S5: Odds ratios for all SMA for all polymorphisms investigated.

Gene	SNPID	Alleles (ancestral / derived)	Chr	DAF Controls	No. of genotype in controls	Sample	Case Phenotype	DAF Cases	No. of genotypes in cases	MO	Unadjusted				Adjusted§			
											OR	LCL	UCL	P	OR	LCL	UCL	P
AJAP1	rs113788643	A/G	1	0.08	3940	All	RD	0.06	683	A	0.81	0.64	1.02	0.0701	0.71	0.43	1.16	0.1850
AJAP1	rs6674631	G/A	1	0.13	3928	All	RD	0.12	683	A	0.90	0.75	1.07	0.2318	1.33	0.92	1.93	0.15938
ATP2B4	rs10900585	G/T	1	0.34	3762	All	RD	0.32	680	R	1.38	1.02	1.87	0.0155	1.13	0.52	2.44	0.0151
ATP2B4	rs1541255	A/G	1	0.33	3933	All	RD	0.30	684	R	0.62	0.46	0.85	0.0019	0.64	0.46	0.88	0.002
ATP2B4	rs3753036	G/A	1	0.04	3939	All	RD	0.04	684	H	0.82	0.59	1.13	0.2162	0.83	0.60	1.14	0.2218
ATP2B4	rs4951074	G/A	1	0.32	3934	All	RD	0.29	683	R	0.65	0.47	0.90	0.0059	0.66	0.52	0.91	0.0057
ATP2B4	rs55868763	G/C	1	0.33	3931	All	RD	0.30	683	R	0.63	0.47	0.87	0.0025	0.65	0.54	0.90	0.0030
CR1	rs17047660	A/G	1	0.16	3923	All	RD	0.17	683	A	1.10	0.94	1.29	0.2219	1.22	0.90	1.66	0.33548
CR1	rs17047661	G/A	1	0.32	3934	All	RD	0.34	683	A	1.08	0.95	1.22	0.2427	1.22	0.73	2.03	0.2094
DARC	rs2814778	G/A	1	0.00	3944	All	RD	0.00	601	H	0.83	0.18	3.71	0.8003	0.87	0.22	4.21	0.34691
GBP7	rs1803632	G/C	1	0.49	3942	All	RD	0.52	683	R	1.22	1.01	1.47	0.0361	1.23	1.10	1.53	0.0360
IL10	rs1800890	A/T	1	0.24	3945	All	RD	0.23	685	R	0.75	0.51	1.09	0.1200	0.75	0.51	1.11	0.12
IL10	rs1800896	T/C	1	0.38	3943	All	RD	0.35	684	D	0.89	0.76	1.05	0.1787	0.74	0.44	1.25	0.2693
IL10	rs3024500	G/A	1	0.45	3928	All	RD	0.48	682	A	1.12	0.99	1.26	0.0658	1.36	0.98	1.90	0.057556
LPHN2	rs146428334	A/G	1	0.00	3930	All	RD	0.00	684	H	1.12	0.47	2.70	0.8015	1.54	0.36	6.61	0.5752
LPHN2	rs4650365	T/C	1	0.20	3936	All	RD	0.19	684	H	0.88	0.74	1.05	0.1587	0.77	0.28	2.11	0.48287
LPHN2	rs72933304	C/A	1	0.09	3942	All	RD	0.07	683	H	0.83	0.66	1.03	0.0975	0.85	0.68	1.07	0.1122
LPHN2	rs72933310	T/C	1	0.09	3927	All	RD	0.08	683	H	0.89	0.72	1.10	0.2667	1.14	0.75	1.74	0.52604
LPHN2	rs72933350	T/C	1	0.05	3940	All	RD	0.05	684	H	2.35	0.60	9.19	0.2479	1.47	0.92	2.35	0.1538
IL1A	rs17561	G/T	2	0.16	3938	All	RD	0.19	685	A	1.20	1.03	1.39	0.0203	2.39	1.13	5.09	0.064612
IL1B	rs1143634	C/T	2	0.11	3941	All	RD	0.11	683	R	0.78	0.35	1.73	0.5252	0.81	0.55	1.20	0.3628
LAPTM4A	rs973128	C/G	2	0.44	3885	All	RD	0.44	675	H	1.05	0.89	1.24	0.5620	1.07	0.90	1.27	0.067
LOC727982	rs10188961	A/G	2	0.40	3934	All	RD	0.43	682	A	1.16	1.03	1.30	0.0165	1.17	0.93	1.47	0.1616
LOC727982	rs1371474	T/C	2	0.27	3933	All	RD	0.29	685	H	1.38	1.17	1.63	0.0001	1.69	1.22	2.35	0.002967
LOC727982	rs1371478	C/T	2	0.27	3909	All	RD	0.29	682	H	1.39	1.18	1.64	0.0001	1.47	1.23	1.74	0.0002
SDC1	rs11899121	C/G	2	0.47	3921	All	RD	0.48	684	H	1.07	0.91	1.26	0.3948	1.27	0.87	1.85	0.29507
ZSWIM2	rs144778284	C/T	2	0.00	3937	All	RD	0.00	684	R	-	-	-	-	-	-	-	-
ZSWIM2	rs4316902	A/G	2	0.18	3910	All	RD	0.16	677	R	0.70	0.41	1.19	0.1706	0.79	0.53	1.16	0.16013
ARL14	rs74954675	A/C	3	0.08	3907	All	RD	0.09	677	H	1.15	0.92	1.44	0.2251	1.05	0.70	1.60	0.8342
ARL14	rs75731597	A/C	3	0.08	3491	All	RD	0.10	670	H	1.23	0.98	1.53	0.0663	1.28	0.99	1.59	0.089
ARL14	rs76033371	A/G	3	0.09	3939	All	RD	0.10	679	H	1.19	0.96	1.47	0.1146	1.08	0.73	1.60	0.7454
B3GALNT1	rs12107243	G/C	3	0.49	3934	All	RD	0.47	683	D	0.87	0.72	1.04	0.1342	0.93	0.74	1.18	0.39756
IL17RD	rs6780995	A/G	3	0.41	3939	All	RD	0.41	685	D	0.98	0.83	1.17	0.8498	0.98	0.63	1.51	0.8753
IL17RE	rs708567	A/G	3	0.50	3881	All	RD	0.49	680	D	0.94	0.78	1.13	0.5045	0.81	0.64	1.02	0.11682
TLR9	rs187084	T/C	3	0.33	3933	All	RD	0.32	601	D	0.87	0.74	1.04	0.1273	0.90	0.81	1.58	0.4593
FREM3	rs149914432	A/C	4	0.10	3915	All	RD	0.06	682	D	0.55	0.43	0.71	9.65×10^{-7}	0.48	0.24	0.95	0.002561
FREM3	rs184895969	C/T	4	0.00	3940	All	RD	0.00	684	H	1.03	0.43	2.48	0.9486	1.04	0.55	3.40	0.0633
FREM3	rs184908374	G/A	4	0.00	3943	All	RD	0.00	684	H	1.05	0.40	2.77	0.9185	1.06	0.50	2.80	0.12848
FREM3	rs186790584	A/T	4	0.10	3941	All	RD	0.06	684	D	0.54	0.42	0.70	6.55×10^{-7}	0.53	0.40	0.7	0.0033
FREM3	rs186873296	A/G	4	0.10	3926	All	RD	0.05	682	D	0.53	0.41	0.69	4.67×10^{-7}	0.61	0.44	0.83	6.45×10^{-5}
GAB1	rs7663712	A/G	4	0.02	3938	All	RD	0.03	684	H	1.21	0.85	1.73	0.3060	1.22	0.19	1.38	0.1530
INPP4B	rs13103597	C/T	4	0.27	3925	All	RD	0.23	683	A	0.78	0.66	0.93	0.0040	0.82	0.72	0.94	0.004952
INPP4B	rs77389579	G/T	4	0.07	3937	All	RD	0.04	683	A	0.58	0.44	0.78	8.53×10^{-5}	0.62	0.46	0.83	9.54×10^{-5}
TLR1	rs4833095	C/T	4	0.09	3921	All	RD	0.10	600	R	1.79	0.84	3.79	0.1499	1.31	0.90	1.89	0.070727
TLR6	rs5743809	T/C	4	0.06	3884	All	RD	0.06	576	R	1.85	0.61	5.67	0.3081	3.71	0.81	17.04	0.1424
TLR6	rs5743810	C/T	4	0.02	3936	All	RD	0.02	602	R	-	-	-	-	-	-	-	-
USP38	rs28459062	T/C	4	0.16	3931	All	RD	0.17	684	R	1.35	0.87	2.11	0.1952	1.05	0.78	1.43	0.5949
USP38	rs4266246	C/T	4	0.22	3928	All	RD	0.22	684	A	1.02	0.88	1.17	0.8077	1.34	0.69	2.61	0.29847
C6	rs1801033	A/C	5	0.47	3927	All	RD	0.48	600	H	1.08	0.91	1.28	0.3912	0.72	0.51	1.02	0.0586
IL13	rs20541	C/T	5	0.22	3796	All	RD	0.22	579	H	0.98	0.81	1.18	0.8427	0.99	0.86	1.48	0.34347
IRF	rs2706384	C/A	5	0.38	3819	All	RD	0.36	586	A	0.94	0.83	1.07	0.3591	1.06	0.67	1.69	0.5697
CTL4	rs2242665	A/G	6	0.26	3853	All	RD	0.28	591	R	1.20	0.87	1.65	0.2804	1.32	1.02	1.70	0.025206
GABBR1	rs192151845	G/A	6	0.01	3943	All	RD	0.01	683	H	1.30	0.70	2.39	0.4154	1.32	0.73	3.67	0.8750
HCG4	rs114980857	C/T	6	0.21	3843	All	RD	0.20	669	A	0.90	0.78	1.04	0.1628	0.89	0.67	1.19	0.57831
HSPA1B	rs6457452	C/T	6	0.19	3917	All	RD	0.19	675	A	0.96	0.83	1.12	0.6348	0.85	0.63	1.16	0.2810
IL20RA	rs1555498	C/T	6	0.46	3947	All	RD	0.48	683	H	0.86	0.73	1.01	0.0613	0.87	0.61	1.24	0.30236
LTA	rs2239704	G/T	6	0.12	3896	All	RD	0.13	673	R	1.60	0.89	2.85	0.1316	1.32	0.91	1.91	0.0873
LTA	rs909253	T/C	6	0.49	3921	All	RD	0.49	680	H	1.13	0.96	1.33	0.1358	1.00	0.79	1.25	0.91093
PLEKHG1	rs114726617	G/A	6	0.04	3940	All	RD	0.05	684	D	1.29	0.96	1.72	0.0980	1.37	0.77	2.44	0.3355
PLEKHG1	rs141555199	C/G	6	0.02	3943	All	RD	0.03	684	H	1.30	0.89	1.89	0.1833	1.32	0.90	1.95	0.67514
PLEKHG1	rs142712208	G/A	6	0.03	3927	All	RD	0.04	685	H	1.36	1.00	1.86	0.0563	1.38	0.74	2.57	0.3974
PLEKHG1	rs15116938	C/T	6	0.03	3939	All	RD	0.04	684	H	1.26	0.92	1.72	0.1537	1.31	0.94	1.82	0.49189
PLEKHG1	rs151293197	C/G	6	0.03	3940	All	RD	0.04	683	H	1.27	0.93	1.73	0.1466	1.32	0.71	2.46	0.4501
PLEKHG1	rs2131263	C/T	6	0.04	3938	All	RD	0.05	684	D	1.29	0.96	1.72	0.0985	1.37	0.77	2.44	0.33629
PLEKHG1	rs55958968	A/G	6	0.05	3932	All	RD	0.06	684	H	1.17	0.90	1.52	0.2575	1.39	0.85	2.25	0.1400
PLEKHG1	rs76924464	G/A	6	0.03	3940	All	RD	0.04	684	H	1.28	0.94	1.74	0.1315	1.29	0.69	2.40	0.48015
PLEKHG1	rs79100774	G/C	6	0.03	3939	All	RD	0.04	683	H	1.25	0.92	1.71	0.1646	1.29	0.69	2.41	0.4816
SNORD48	rs116288147	C/T	6	0.24	3896	All	RD	0.24	675	R	1.05	0.75	1.49	0.7679	1.07	0.81	1.59	0.7787
TNF	rs1799964	T/C	6	0.27	3938	All	RD	0.27	676	R	0.91	0.66	1.26	0.5853	0.95	0.70	1.34	0.6790
TNF	rs1800629	G/A	6	0.09	3940	All	RD	0.09	684	R	1.92	0.90	4.09	0.1094	1.97	0.98	4.95	0.1199
TNF	rs1800750	G/A	6	0.05	3868	All	RD	0.04	352	H	0.71	0.47	1.08	0.0987	0.67	0.25	1.81	0.3678
TNF	rs3093662	A/G	6	0.11	3936	All	RD	0.11	682	R	0.53	0.21	1.33	0.1384	0.87	0.57	1.32	0.54979
TNF	rs361525	G/A	6	0.07	3948	All	RD	0.07	685	R	0.52	0.12	2.22	0.3342	0.54	0.19	3.21	0.1810
CD36	rs3211938	T/G	7	0.10	3942	All	RD	0.09	683	H	0.84	0.67	1.06	0.1389	0.89	0.57	1.40	0.57785
NOD1	rs2075820	G/A	7	0.38	3941	All	RD	0.39	602	H	1.12	0.94	1.33	0.2032	1.08	0.78	1.51	0.7891
ABO	rs56390333	G/A	9	0.00	3942	All	RD	0.00	682	H	-	-	-	-	-	-	-	-
ABO	rs8176719*	O vs non-O	9	0.26	3888	All	RD	0.31	683	R	0.70	0.59	0.83	2.74×10^{-5}	0.71	0.48	0.91	6.45×10^{-5}
ABO	rs8176746*	C/A	9	0.13	3924	All	RD	0.18	683	A	2.55	1.66	3.92	1.79×10^{-5}	2.42	1.56	3.76	8.04×10^{-5}
ABO	rs8176750	I/D	9	0.05	3923	All	RD	0.04	680	A	0.79	0.59	1.06	0.1116	0.66	0.36	1.20	0.6042
TLR4	rs4986790	A/G	9	0.04	3929	All	RD	0.05	600	R	0.69	0.32	1.32	0.2571	0.70	0.36	1.36	0.25752
TLR4	rs4986791	C/T	9	0.01	3937	All	RD	0.01	599	H	1.40	0.70	2.78	0.3590	2.43	0.85	6.94	0.1839
MKI67	rs11016116	A/G	10	0.02	3944	All	RD	0.03	684	A	1.07	0.74	1.53	0.7325	1.11	0.78	1.78	0.9345
MKI67	rs115947774	A/G	10	0.00	3931	All	RD	0.00	682	H	0.37	0.09	1.57	0.1208	0.44	0.10	1.86	0.1453
MKI67	rs148494166	T/C	10	0.00	3913	All	RD	0.00	654	H	1.93	0.70	5.33	0.2324	1.94	0.72	6.30	0.56499

HBB	rs334	A/T	11	0.08	3949	All	RD	0.02	684	H	0.11	0.06	0.20	8.41×10^{-28}	0.11	0.06	0.20	1.62×10^{-27}
RRM1	rs116472045	A/G	11	0.02	3939	All	RD	0.01	682	R	-	-	-	-	-	-	-	-
RTN3	rs542998	T/C	11	0.43	3929	All	RD	0.44	592	R	1.16	0.93	1.43	0.1864	0.82	0.58	1.15	0.3977
TRIM5	rs7935564	G/A	11	0.44	3871	All	RD	0.47	598	A	1.15	1.01	1.30	0.0289	1.29	0.87	1.90	0.091014
CAND1	rs10459266	C/A	12	0.21	3938	All	RD	0.23	684	D	1.22	1.03	1.44	0.0217	1.75	1.26	2.42	0.0009
CAND1	rs12307123	C/T	12	0.23	3933	All	RD	0.25	682	D	1.24	1.05	1.46	0.0113	1.17	0.90	1.52	0.17144
CAND1	rs1566830	G/T	12	0.26	3940	All	RD	0.29	684	A	1.20	1.06	1.37	0.0049	1.76	1.04	2.98	0.0409
GRIP1	rs1394263	T/C	12	0.19	3944	All	RD	0.18	683	H	0.92	0.77	1.09	0.3299	0.95	0.90	2.95	0.3499
IL22	rs1012356	A/T	12	0.49	3940	All	RD	0.49	683	H	1.08	0.92	1.28	0.3359	1.09	0.93	1.31	0.4368
IL22	rs2227478	A/G	12	0.38	3939	All	RD	0.38	685	R	0.89	0.71	1.13	0.3382	0.90	0.72	1.73	0.24654
IL22	rs2227485	G/A	12	0.42	3928	All	RD	0.43	682	H	1.12	0.95	1.32	0.1632	1.14	0.97	1.33	0.1789
IL22	rs2227491	C/T	12	0.40	3870	All	RD	0.40	672	R	0.92	0.74	1.16	0.4873	0.97	0.81	1.57	0.38947
IL22	rs2227507	T/C	12	0.04	3948	All	RD	0.05	685	H	1.09	0.81	1.46	0.5699	1.10	0.82	1.48	0.4098
TPTE2	rs182873742	C/G	13	0.00	3940	All	RD	0.00	678	H	-	-	-	-	-	-	-	-
RPS6KL1	rs3742785	C/A	14	0.29	3714	All	RD	0.28	656	H	1.06	0.89	1.26	0.4599	1.06	0.89	1.27	0.5001
SPTB	rs229587	T/C	14	0.34	3872	All	RD	0.34	677	R	1.03	0.80	1.32	0.8388	1.09	0.89	1.41	0.10252
YLPM1	rs10139016	C/T	14	0.25	3937	All	RD	0.24	684	R	0.88	0.63	1.25	0.4820	0.78	0.37	1.61	0.5046
ADCY9	rs10775349	C/G	16	0.20	3941	All	RD	0.20	601	R	1.26	0.84	1.89	0.2805	1.90	0.97	3.71	0.11955
ADCY9	rs2230739	A/G	16	0.09	3944	All	RD	0.09	602	R	0.64	0.20	2.12	0.4431	0.67	0.32	3.14	0.4588
HBA	$\alpha^{-3.7}$-thalassaemia	I/D	16	0.41	3946	All	RD	0.35	662	A	0.78	0.69	0.89	0.0001	0.79	0.70	0.90	0.0002
IL4	rs2243250	T/C	16	0.20	3902	All	RD	0.22	678	H	1.16	0.98	1.38	0.0868	1.21	0.98	1.78	0.1230
IL4R	rs1805015	T/C	16	0.42	3927	All	RD	0.42	683	R	0.87	0.70	1.09	0.2154	1.12	0.81	1.56	0.63147
ADORA2B	rs2535611	T/C	17	0.10	3911	All	RD	0.13	600	A	1.23	1.03	1.49	0.0289	1.28	0.86	1.89	0.2169
NOS2	rs1800482	G/C	17	0.08	3922	All	RD	0.07	679	D	0.94	0.75	1.19	0.6226	0.98	0.56	1.21	0.40375
NOS2	rs2297518	G/A	17	0.14	3910	All	RD	0.14	598	R	1.17	0.65	2.08	0.6096	2.02	0.85	4.79	0.1207
NOS2	rs8078340	C/T	17	0.21	3948	All	RD	0.20	684	R	0.59	0.36	0.99	0.0311	0.61	0.40	0.99	0.032
NOS2	rs9282799	C/T	17	0.03	3949	All	RD	0.03	682	H	1.19	0.84	1.69	0.3324	1.21	0.87	1.72	0.3590
TBX2	rs73991577	A/G	17	0.00	3937	All	RD	0.00	683	H	0.27	0.04	2.05	0.1239	0.30	0.10	2.95	0.21332
EMR1	rs373533	G/T	19	0.46	3863	All	RD	0.47	598	H	1.09	0.92	1.29	0.3338	1.04	0.72	1.51	0.9102
EMR1	rs461645	C/T	19	0.47	3933	All	RD	0.48	601	H	1.11	0.94	1.32	0.2165	1.07	0.73	1.55	0.80482
ICAM	rs5498	A/G	19	0.11	3908	All	RD	0.13	673	H	1.16	0.95	1.41	0.1383	1.16	0.98	3.22	0.3534
GNAS	rs8386	C/T	20	0.26	3914	All	RD	0.29	683	A	1.22	1.07	1.38	0.0028	1.25	1.10	1.43	0.0029
DERL3	rs1128127	G/A	22	0.50	3911	All	RD	0.47	683	A	0.92	0.82	1.03	0.1591	0.88	0.61	1.26	0.3972
CD40LG	rs1126535	T/C	X	0.15	3934	M+F	RD	0.15	683	H	0.82	0.62	1.08	0.1493	0.59	0.37	0.94	0.020226
CD40LG	rs1126535	T/C	X	0.16	1947	F	RD	0.14	326	D	0.82	0.62	1.07	0.1314	0.84	0.63	1.09	0.1414
CD40LG	rs1126535	C/T	X	0.85	1987	M	RD	0.83	357	HM	0.87	0.64	1.19	0.3922	0.88	0.65	1.20	0.41218
CD40LG	rs3092945	T/C	X	0.21	3927	M+F	RD	0.24	680	H	1.28	1.01	1.63	0.0468	1.15	0.81	1.64	0.3059
CD40LG	rs3092945	T/C	X	0.21	1942	F	RD	0.24	325	D	1.29	1.01	1.63	0.0387	1.31	1.03	1.66	0.02
CD40LG	rs3092945	C/T	X	0.78	1985	M	RD	0.77	355	HM	0.95	0.72	1.24	0.7021	0.93	0.71	1.21	0.6100
G6PD	rs1050828	C/T	X	0.19	3940	M+F	RD	0.18	680	D	0.90	0.74	1.09	0.2865	1.05	0.64	1.70	0.64676
G6PD	rs1050828	C/T	X	0.20	1951	F	RD	0.19	325	H	0.89	0.58	1.38	0.6110	0.88	0.68	1.14	0.3500
G6PD	rs1050828	C/T	X	0.19	1989	M	RD	0.17	355	HM	0.89	0.66	1.21	0.8900	0.87	0.64	1.17	0.36
G6PD	rs1050829	T/C	X	0.40	3928	M+F	RD	0.40	681	H	0.88	0.70	1.12	0.2922	0.79	0.51	1.21	0.4246
G6PD	rs1050829	T/C	X	0.41	1952	F	RD	0.40	325	H	0.89	0.70	1.12	0.3148	0.91	0.72	1.16	0.32475
G6PD	rs1050829	C/T	X	0.60	1976	M	RD	0.61	356	HM	1.01	0.80	1.28	0.9095	1.02	0.82	1.30	0.9195

* The rs8176719 polymorphism identifies blood-group O in the homozygous deletion form. The derived allele of rs8176746 identifies blood-group B. Chr = chromosome; DAF = derived allele frequency; MO = genotype model [A – additive, D – dominant, H – heterozygote, HM – hemizygous males, R – recessive] with respect to the derived allele; OR = Odds Ratio; LCI = Lower Confidence Interval (95%); UCI = Upper Confidence Interval; P = p-value using a logistic regression model as described in methods. § ORs and p-values unadjusted and adjusted for ethnic group, gender and rs334 genotype. All individuals having respiratory distress were included in this analysis even if they were identified with multiple severe phenotypes.

Table S6: Odds ratios for all RD for all polymorphisms investigated.

Gene	SNP ID	Alleles (ancestral / derived)	Chr	DAF Controls	No. of genotype in controls	Sample	Case Phenotype	DAF Cases	No. of genotypes in cases	MO	Unadjusted				Adjusted§			
											OR	LCL	UCL	P	OR	LCL	UCL	P
AJAP1	rs113788643	A/G	1	0.08	3940	All	Other SM	0.08	430	R	-	-	-	-	-	-	-	-
AJAP1	rs6674631	G/A	1	0.13	3928	All	Other SM	0.13	431	R	0.89	0.43	1.88	0.7641	0.96	0.46	2.04	0.78675
ATP2B4	rs10900585	G/T	1	0.34	3762	All	Other SM	0.35	426	H	1.19	1.05	1.64	0.0123	1.04	0.63	1.69	0.1783
ATP2B4	rs1541255	A/G	1	0.33	3933	All	Other SM	0.33	431	R	0.84	0.60	1.18	0.3120	0.85	0.60	1.21	0.34498
ATP2B4	rs3753036	G/A	1	0.04	3939	All	Other SM	0.03	432	A	0.82	0.56	1.20	0.2965	0.78	0.52	1.17	0.2440
ATP2B4	rs4951074	G/A	1	0.32	3934	All	Other SM	0.32	431	R	0.89	0.63	1.27	0.5203	1.07	0.73	1.59	0.51529
ATP2B4	rs55868763	G/C	1	0.33	3931	All	Other SM	0.33	431	R	0.85	0.61	1.20	0.3462	0.90	0.62	1.29	0.3814
CR1	rs17047660	A/G	1	0.16	3923	All	Other SM	0.16	436	H	0.85	0.67	1.07	0.1564	0.83	0.65	1.05	0.14157
CR1	rs17047661	G/A	1	0.32	3934	All	Other SM	0.31	434	R	0.91	0.64	1.30	0.6158	0.86	0.59	1.25	0.5502
DARC	rs2814778	G/A	1	0.00	3944	All	Other SM	0.00	379	H	0.58	0.07	4.45	0.5675	0.59	0.08	4.56	0.56766
GBP7	rs1803632	G/C	1	0.49	3942	All	Other SM	0.51	434	R	1.18	0.94	1.47	0.1615	1.12	0.89	1.41	0.1942
IL10	rs1800890	A/T	1	0.24	3945	All	Other SM	0.21	434	R	0.42	0.23	0.76	0.0013	0.43	0.23	0.79	0.001385
IL10	rs1800896	T/C	1	0.38	3943	All	Other SM	0.35	433	R	0.81	0.60	1.11	0.1788	0.91	0.78	1.06	0.1865
IL10	rs3024500	G/A	1	0.45	3928	All	Other SM	0.48	434	R	1.18	0.93	1.50	0.1701	1.18	0.92	1.50	0.17602
LPHN2	rs146428334	A/G	1	0.00	3930	All	Other SM	0.01	430	H	1.55	0.60	4.04	0.3916	1.61	0.61	4.26	0.3839
LPHN2	rs4650365	T/C	1	0.20	3936	All	Other SM	0.17	432	A	0.80	0.66	0.97	0.0183	0.82	0.67	1.00	0.020669
LPHN2	rs72933304	C/A	1	0.09	3942	All	Other SM	0.06	431	D	0.67	0.49	0.91	0.0076	0.68	0.50	0.94	0.0092
LPHN2	rs72933310	T/C	1	0.09	3927	All	Other SM	0.07	431	A	0.71	0.54	0.94	0.0132	0.73	0.55	0.97	0.016043
LPHN2	rs72933350	T/C	1	0.05	3940	All	Other SM	0.03	430	A	0.61	0.41	0.91	0.0095	0.66	0.44	0.99	0.0109
IL1A	rs17561	G/T	2	0.16	3938	All	Other SM	0.19	434	R	1.89	1.16	3.09	0.0162	1.94	1.16	3.22	0.02727
IL1B	rs1143634	C/T	2	0.11	3941	All	Other SM	0.14	434	A	1.20	0.97	1.47	0.0905	1.20	0.95	1.52	0.1054
LAPTM4A	rs973128	C/G	2	0.44	3885	All	Other SM	0.43	419	R	0.86	0.66	1.13	0.2753	0.87	0.66	1.15	0.33759
LOC727982	rs10188961	A/G	2	0.40	3934	All	Other SM	0.44	431	A	1.20	1.04	1.39	0.0123	1.23	1.06	1.43	0.0082
LOC727982	rs1371474	T/C	2	0.27	3933	All	Other SM	0.29	430	D	1.18	0.97	1.45	0.1032	1.22	0.99	1.51	0.077484
LOC727982	rs1371478	C/T	2	0.27	3909	All	Other SM	0.29	429	D	1.18	0.97	1.45	0.0998	1.23	1.00	1.52	0.0751
SDC1	rs11899121	C/G	2	0.47	3921	All	Other SM	0.44	429	R	0.81	0.62	1.04	0.0902	0.84	0.65	1.09	0.11306
ZSWIM2	rs144778284	C/T	2	0.00	3937	All	Other SM	0.00	431	H	0.98	0.29	3.31	0.9741	1.11	0.32	3.80	0.9907
ZSWIM2	rs4316902	A/G	2	0.18	3910	All	Other SM	0.16	430	R	0.42	0.18	0.96	0.0188	0.38	0.16	0.95	0.007496
ARL14	rs74954675	A/C	3	0.08	3907	All	Other SM	0.10	429	A	1.15	0.91	1.47	0.2497	1.17	0.91	1.50	0.2553
ARL14	rs75731597	A/C	3	0.08	3491	All	Other SM	0.10	424	D	1.21	0.93	1.58	0.1547	1.25	0.95	1.64	0.16415
ARL14	rs76033371	A/G	3	0.09	3939	All	Other SM	0.10	431	D	1.14	0.88	1.47	0.3286	1.17	0.90	1.52	0.3341
B3GALNT1	rs12107243	G/C	3	0.49	3934	All	Other SM	0.50	430	R	1.10	0.87	1.38	0.4298	1.16	0.91	1.47	0.34802
IL17RD	rs6780995	A/G	3	0.41	3939	All	Other SM	0.41	433	H	1.27	1.04	1.55	0.0207	1.26	1.03	1.55	0.0164
IL17RE	rs708567	A/G	3	0.50	3881	All	Other SM	0.49	430	R	0.84	0.66	1.07	0.1501	1.14	0.93	1.40	0.14898
TLR9	rs187084	T/C	3	0.33	3933	All	Other SM	0.34	381	H	1.10	0.89	1.36	0.3896	1.13	0.91	1.41	0.3953
FREM3	rs149914432	A/C	4	0.10	3915	All	Other SM	0.06	429	A	0.62	0.47	0.83	0.0006	0.76	0.53	0.86	0.000667
FREM3	rs184895969	C/T	4	0.00	3940	All	Other SM	0.00	431	H	0.50	0.12	2.11	0.2983	0.54	0.12	2.42	0.3061
FREM3	rs184908374	G/A	4	0.00	3943	All	Other SM	0.00	432	H	0.61	0.14	2.59	0.4702	0.67	0.15	3.02	0.48043
FREM3	rs186790584	A/T	4	0.10	3941	All	Other SM	0.06	432	D	0.57	0.42	0.78	0.0002	0.60	0.43	0.8	0.0002
FREM3	rs186873296	A/G	4	0.10	3926	All	Other SM	0.05	428	D	0.55	0.40	0.75	0.0001	0.56	0.42	0.78	0.000195
GAB1	rs7663712	A/G	4	0.02	3938	All	Other SM	0.02	431	R	-	-	-	-	-	-	-	-
INPP4B	rs13103597	C/T	4	0.27	3925	All	Other SM	0.24	431	A	0.88	0.75	1.04	0.1337	0.99	0.82	1.19	0.11272
INPP4B	rs77389579	G/T	4	0.07	3937	All	Other SM	0.04	430	D	0.53	0.37	0.77	0.0003	0.60	0.43	0.8	0.0004
TLR1	rs4833095	C/T	4	0.09	3921	All	Other SM	0.09	380	R	0.61	0.14	2.56	0.4632	0.54	0.13	2.31	0.46587
TLR6	rs5743809	T/C	4	0.06	3884	All	Other SM	0.07	358	A	1.31	0.97	1.76	0.0857	1.35	0.99	1.82	0.0759
TLR6	rs5743810	C/T	4	0.02	3936	All	Other SM	0.02	380	R	-	-	-	-	-	-	-	-
USP38	rs28459062	T/C	4	0.16	3931	All	Other SM	0.17	432	H	1.26	1.01	1.56	0.0417	1.18	0.94	1.48	0.0472
USP38	rs4266246	C/T	4	0.22	3928	All	Other SM	0.21	429	H	0.92	0.75	1.15	0.4720	1.10	0.69	1.74	0.50541
C6	rs1801033	A/C	5	0.47	3927	All	Other SM	0.47	380	H	0.92	0.75	1.14	0.4473	0.93	0.74	1.15	0.3876
IL13	rs20541	C/T	5	0.22	3796	All	Other SM	0.22	360	H	1.01	0.80	1.28	0.9132	1.02	0.81	1.29	0.81604
IRF	rs2706384	C/A	5	0.38	3819	All	Other SM	0.35	365	D	0.89	0.72	1.11	0.3095	0.90	0.72	1.13	0.3295
CTL4	rs2242665	A/G	6	0.26	3853	All	Other SM	0.32	378	A	1.32	1.12	1.55	0.0011	1.33	1.13	1.58	0.001307
GABBR1	rs192151845	G/A	6	0.01	3943	All	Other SM	0.01	431	H	0.97	0.41	2.27	0.9385	0.86	0.34	2.14	0.9572
HCG4	rs114980857	C/T	6	0.21	3843	All	Other SM	0.22	426	D	1.04	0.85	1.28	0.7093	1.01	0.85	1.21	0.77903
HSPA1B	rs6457452	C/T	6	0.19	3917	All	Other SM	0.16	434	D	0.79	0.64	0.98	0.0332	0.81	0.65	1.01	0.0233
IL20RA	rs1555498	C/T	6	0.46	3947	All	Other SM	0.51	434	D	1.33	1.05	1.68	0.0163	1.29	1.01	1.65	0.016524
LTA	rs2239704	G/T	6	0.12	3896	All	Other SM	0.15	425	H	1.37	1.09	1.72	0.0083	1.28	1.01	1.62	0.0112
LTA	rs909253	T/C	6	0.49	3921	All	Other SM	0.46	428	A	0.92	0.80	1.06	0.2466	0.90	0.70	1.15	0.28612
PLEKHG1	rs114726617	G/A	6	0.04	3940	All	Other SM	0.04	431	R	-	-	-	-	-	-	-	-
PLEKHG1	rs141555199	C/G	6	0.02	3943	All	Other SM	0.03	432	H	1.55	1.02	2.35	0.0514	1.60	1.02	2.49	0.043208
PLEKHG1	rs142712208	G/A	6	0.03	3927	All	Other SM	0.04	431	R	-	-	-	-	-	-	-	-
PLEKHG1	rs15116938	C/T	6	0.03	3939	All	Other SM	0.04	430	R	-	-	-	-	-	-	-	-
PLEKHG1	rs151293197	C/G	6	0.03	3940	All	Other SM	0.03	432	R	-	-	-	-	-	-	-	-
PLEKHG1	rs2131263	C/T	6	0.04	3938	All	Other SM	0.04	430	R	-	-	-	-	-	-	-	-
PLEKHG1	rs55958968	A/G	6	0.05	3932	All	Other SM	0.06	431	H	1.25	0.91	1.72	0.1760	1.22	0.88	1.70	0.1995
PLEKHG1	rs76924464	G/A	6	0.03	3940	All	Other SM	0.03	431	R	-	-	-	-	-	-	-	-
PLEKHG1	rs79100774	G/C	6	0.03	3939	All	Other SM	0.03	432	R	-	-	-	-	-	-	-	-
SNORD48	rs116288147	C/T	6	0.24	3896	All	Other SM	0.21	428	D	0.80	0.65	0.98	0.0331	0.82	0.66	1.01	0.026942
TNF	rs1799964	T/C	6	0.27	3938	All	Other SM	0.27	431	H	0.85	0.69	1.05	0.1238	0.84	0.68	1.04	0.1314
TNF	rs1800629	G/A	6	0.09	3940	All	Other SM	0.08	433	A	0.80	0.61	1.04	0.0862	0.78	0.59	1.03	0.10066
TNF	rs1800750	G/A	6	0.05	3868	All	Other SM	0.05	232	H	0.86	0.53	1.40	0.5408	0.74	0.44	1.24	0.4297
TNF	rs3093662	A/G	6	0.11	3936	All	Other SM	0.11	431	A	0.94	0.75	1.19	0.6219	0.94	0.75	1.19	0.62045
TNF	rs361525	G/A	6	0.07	3948	All	Other SM	0.06	432	H	0.87	0.63	1.19	0.3623	0.82	0.60	1.14	0.3187
CD36	rs3211938	T/G	7	0.10	3942	All	Other SM	0.11	435	A	1.19	0.95	1.49	0.1294	1.24	0.99	1.56	0.13664
NOD1	rs2075820	G/A	7	0.38	3941	All	Other SM	0.37	381	H	0.93	0.75	1.15	0.5078	0.98	0.78	1.21	0.4948
ABO	rs56390333	G/A	9	0.00	3942	All	Other SM	0.00	432	H	-	-	-	-	-	-	-	-
ABO	rs8176719*	O vs non-O	9	0.26	3888	All	Other SM	0.32	433	R	0.72	0.50	0.81	0.0005	0.76	0.52	0.85	0.0006
ABO	rs8176746*	C/A	9	0.13	3924	All	Other SM	0.19	434	A	1.51	1.26	1.81	0.00002	1.36	1.08	1.71	$1.92x10^{-5}$
ABO	rs8176750	I/D	9	0.05	3923	All	Other SM	0.05	431	R	0.81	0.10	6.39	0.8405	0.58	0.07	4.80	0.8486
TLR4	rs4986790	A/G	9	0.04	3929	All	Other SM	0.04	377	R	-	-	-	-	-	-	-	-
TLR4	rs4986791	C/T	9	0.01	3937	All	Other SM	0.01	381	H	0.87	0.31	2.42	0.7807	1.00	0.35	2.82	0.7845
MKI67	rs11016116	A/G	10	0.02	3944	All	Other SM	0.02	432	A	0.70	0.41	1.19	0.1672	0.26	0.04	1.97	0.11184
MKI67	rs115947774	A/G	10	0.00	3931	All	Other SM	0.00	429	H	0.27	0.04	1.96	0.1072	0.59	0.08	4.60	0.6791
MKI67	rs148494166	T/C	10	0.00	3913	All	Other SM	0.00	415	H	0.66	0.09	5.04	0.6667	0.76	0.45	1.30	0.18346
HBB	rs334	A/T	11	0.08	3949	All	Other SM	0.02	434	A	0.24	0.14	0.38	$2.6x10^{-13}$	0.22	0.14	0.37	$4.1x10^{-13}$

RRM1	rs116472045	A/G	11	0.02	3939	All	Other SM	0.02	431	H	1.21	0.70	2.10	0.5093	1.22	0.70	2.13	0.47035
RTN3	rs542998	T/C	11	0.43	3929	All	Other SM	0.47	375	D	1.28	1.01	1.62	0.0402	1.31	1.02	1.67	0.0343
TRIM5	rs7935564	G/A	11	0.44	3871	All	Other SM	0.46	378	R	1.21	0.93	1.56	0.1638	1.08	0.83	1.41	0.15605
CAND1	rs10459266	C/A	12	0.21	3938	All	Other SM	0.23	432	D	1.16	0.94	1.42	0.1658	1.17	0.95	1.45	0.1441
CAND1	rs12307123	C/T	12	0.23	3933	All	Other SM	0.22	432	H	0.99	0.80	1.22	0.9170	1.00	0.84	1.19	0.8852
CAND1	rs1566830	G/T	12	0.26	3940	All	Other SM	0.26	431	R	1.14	0.77	1.68	0.5241	1.14	0.76	1.69	0.4937
GRIP1	rs1394263	T/C	12	0.26	3914	All	Other SM	0.26	428	R	1.18	0.80	1.73	0.4130	1.17	0.79	1.73	0.38613
IL22	rs1012356	A/T	12	0.49	3940	All	Other SM	0.48	433	H	1.16	0.95	1.42	0.1362	1.17	0.95	1.44	0.0929
IL22	rs2227478	A/G	12	0.38	3939	All	Other SM	0.40	433	H	0.86	0.70	1.05	0.1377	0.90	0.73	1.11	0.17122
IL22	rs2227485	G/A	12	0.42	3928	All	Other SM	0.41	432	R	0.88	0.67	1.15	0.3423	0.84	0.64	1.12	0.3101
IL22	rs2227491	C/T	12	0.40	3870	All	Other SM	0.43	429	D	1.24	1.00	1.54	0.0520	1.24	0.99	1.56	0.035985
IL22	rs2227507	T/C	12	0.04	3948	All	Other SM	0.04	435	R	0.91	0.11	7.33	0.9290	0.80	0.10	6.56	0.9341
TPTE2	rs182873742	C/G	13	0.00	3940	All	Other SM	0.00	427	H	-	-	-	-	-	-	-	-
RPS6KL1	rs3742785	C/A	14	0.29	3714	All	Other SM	0.30	406	H	1.21	0.98	1.49	0.0746	1.14	0.92	1.42	0.1143
SPTB	rs229587	T/C	14	0.34	3872	All	Other SM	0.37	422	R	1.36	1.02	1.81	0.0398	1.34	1.00	1.80	0.043333
YLPM1	rs10139016	C/T	14	0.25	3937	All	Other SM	0.26	431	R	1.15	0.78	1.69	0.4848	1.10	0.74	1.64	0.4388
ADCY9	rs10775349	C/G	16	0.20	3941	All	Other SM	0.20	379	H	0.89	0.71	1.12	0.3107	0.87	0.68	1.10	0.30031
ADCY9	rs2230739	A/G	16	0.09	3944	All	Other SM	0.07	380	H	0.72	0.52	1.00	0.0407	0.69	0.50	0.97	0.0431
HBA	$\alpha^{-3.7}$-thalassaemia	I/D	16	0.41	3946	All	Other SM	0.37	421	D	0.80	0.65	0.98	0.0322	0.80	0.64	0.98	0.042911
IL4	rs2243250	T/C	16	0.20	3902	All	Other SM	0.21	428	R	1.12	0.70	1.79	0.6423	1.03	0.86	1.23	0.7517
IL4R	rs1805015	T/C	16	0.42	3927	All	Other SM	0.42	434	A	0.96	0.84	1.11	0.6062	0.99	0.86	1.15	0.71347
ADORA2B	rs2535611	T/C	17	0.10	3911	All	Other SM	0.11	380	R	1.76	0.82	3.80	0.1753	1.76	0.81	3.84	0.1728
NOS2	rs1800482	G/C	17	0.08	3922	All	Other SM	0.08	432	R	0.36	0.05	2.66	0.2383	0.31	0.04	2.40	0.24072
NOS2	rs2297518	G/A	17	0.14	3910	All	Other SM	0.14	377	A	1.02	0.82	1.26	0.8720	1.04	0.84	1.30	0.8338
NOS2	rs8078340	C/T	17	0.21	3948	All	Other SM	0.23	435	R	1.37	0.88	2.13	0.1832	1.28	0.82	2.02	0.17457
NOS2	rs9282799	C/T	17	0.03	3949	All	Other SM	0.02	432	R	-	-	-	-	-	-	-	-
TBX2	rs73991577	A/G	17	0.00	3937	All	Other SM	0.00	430	R	-	-	-	-	-	-	-	-
EMR1	rs373533	G/T	19	0.46	3863	All	Other SM	0.51	379	R	1.31	1.03	1.67	0.0303	1.32	1.03	1.68	0.0260
EMR1	rs461645	C/T	19	0.47	3933	All	Other SM	0.52	380	R	1.33	1.05	1.69	0.0194	1.33	1.04	1.70	0.016471
ICAM	rs5498	A/G	19	0.11	3908	All	Other SM	0.12	427	R	1.12	0.53	2.38	0.7671	1.10	0.51	2.36	0.7497
GNAS	rs8386	C/T	20	0.19	3944	All	Other SM	0.16	434	A	0.84	0.70	1.02	0.0755	0.84	0.69	1.02	0.057606
DERL3	rs1128127	G/A	22	0.50	3911	All	Other SM	0.48	434	D	0.95	0.76	1.18	0.6360	0.98	0.78	1.23	0.6265
CD40LG	rs1126535	T/C	X	0.15	3934	M+F	Other SM	0.15	434	H	0.95	0.68	1.31	0.7387	0.91	0.65	1.28	0.69642
CD40LG	rs1126535	T/C	X	0.16	1947	F	Other SM	0.14	209	R	0.35	0.09	1.47	0.0942	0.37	0.10	1.48	0.0952
CD40LG	rs1126535	C/T	X	0.85	1987	M	Other SM	0.83	225	HM	0.87	0.60	1.26	0.4637	0.89	0.61	1.28	0.4837
CD40LG	rs3092945	T/C	X	0.21	3927	M+F	Other SM	0.26	434	D	1.30	1.05	1.61	0.0165	1.25	1.00	1.56	0.0183
CD40LG	rs3092945	T/C	X	0.21	1942	F	Other SM	0.26	210	D	1.35	1.01	1.80	0.0431	1.36	1.03	1.81	0.0451
CD40LG	rs3092945	C/T	X	0.78	1985	M	Other SM	0.73	224	HM	0.80	0.59	1.10	0.1835	0.81	0.60	1.15	0.2135
G6PD	rs1050828	C/T	X	0.19	3940	M+F	Other SM	0.19	431	H	0.75	0.55	1.04	0.0826	0.73	0.52	1.02	0.082567
G6PD	rs1050828	C/T	X	0.20	1951	F	Other SM	0.17	207	H	0.75	0.55	1.04	0.0827	0.76	0.56	1.06	0.0927
G6PD	rs1050828	C/T	X	0.19	1989	M	Other SM	0.20	224	HM	1.07	0.76	1.52	0.6975	1.09	0.78	1.55	0.7075
G6PD	rs1050829	T/C	X	0.40	3928	M+F	Other SM	0.41	433	H	0.75	0.56	1.01	0.0561	0.83	0.61	1.12	0.0876
G6PD	rs1050829	T/C	X	0.41	1952	F	Other SM	0.41	208	H	0.75	0.56	1.01	0.0555	0.76	0.57	1.02	0.0655
G6PD	rs1050829	C/T	X	0.60	1976	M	Other SM	0.59	225	HM	0.94	0.71	1.24	0.6541	0.95	0.73	1.25	0.6741

* The rs8176719 polymorphism identifies blood-group O in the homozygous deletion form. The derived allele of rs8176746 identifies blood-group B. Chr = chromosome; DAF = derived allele frequency; MO = genotype model [A – additive, D – dominant, H – heterozygote, HM – hemizygous males, R – recessive] with respect to the derived allele; OR = Odds Ratio; LCI = Lower Confidence Interval (95%); UCI = Upper Confidence Interval; P = p-value using a logistic regression model as described in methods. § ORs and p-values unadjusted and adjusted for ethnic group, gender and rs334 genotype. Other SM includes those individuals who did not have CM, SMA or RD.

Table S7: Odds ratios for other SM for all polymorphisms investigated.

Gene	SNPID	Genotype	SM		CM		SMA		RD		Death	
			OR (95% CI)	P†	OR (95% CI)	P†	OR (95% CI)	P†	OR (95% CI)	P†	OR (95% CI)	P†
ATP2B4	rs1541255	norm (AA)	1		1		1		1		1	
		het (AG)	0.98 (0.88-1.10)	0.8	0.91 (0.80-1.05)	0.22	0.89 (0.75-1.06)	0.22	0.94 (0.79-1.12)	0.53	0.97 (0.74-1.26)	0.83
		hom (GG)	0.73 (0.60-0.89)	0.001	0.65 (0.51-0.83)	0.0007	0.60 (0.44-0.83)	0.002	0.61 (0.44-0.84)	0.002	0.79 (0.49-1.25)	0.32
IL10	rs1800890	norm (AA)	1		1		1		1		1	
		het (AT)	1.03 (0.92-1.15)	0.59	1.01 (0.88-1.15)	0.82	1.06 (0.89-1.26)	0.45	1.03 (0.86-1.22)	0.71	0.90 (0.69-1.19)	0.48
		hom (TT)	0.70 (0.55-0.89)	0.004	0.74 (0.54-1.00)	0.05	0.85 (0.59-1.23)	0.4	0.76 (0.52-1.11)	0.15	0.93 (0.53-1.58)	0.79
LPHN2	rs72933304	norm (CC)	1		1		1		1		1	
		het (AC)	0.82 (0.70-0.95)	0.01	0.84 (0.69-1.01)	0.07	0.86 (0.68-1.09)	0.23	0.84 (0.66-1.06)	0.15	0.79 (0.55-1.15)	0.23
		hom (AA)	0.46 (0.20-1.02)	0.05	0.51 (0.19-1.35)	0.18	0.58 (0.17-1.96)	0.38	0.58 (0.17-1.94)	0.38	-	-
LOC727982	rs1371478	norm (CC)	1		1		1		1		1	
		het (CT)	1.30 (1.16-1.49)	2.70×10^{-6}	1.31 (1.14-1.50)	9.41×10^{-5}	1.34 (1.12-1.59)	0.0008	1.37 (1.15-1.62)	0.0002	1.37 (1.05-1.78)	0.01
		hom (TT)	1.02 (0.83-1.26)	0.78	0.96 (0.74-1.25)	0.79	1.16 (0.85-1.59)	0.35	0.89 (0.63-1.26)	0.53	1.12 (0.69-1.81)	0.62
ARL14	rs75731597	norm (AA)	1		1		1		1		1	
		het (AC)	1.26 (1.09-1.46)	0.001	1.25 (1.04-1.49)	0.01	1.25 (1.02-1.59)	0.03	1.22 (0.98-1.53)	0.06	1.55 (1.13-2.14)	0.006
		hom (CC)	0.80 (0.45-1.43)	0.46	0.73 (0.35-1.55)	0.43	1.07 (0.47-2.44)	0.86	0.73 (0.28-1.88)	0.52	1.23 (0.37-4.07)	0.72
FREM3	rs186873296	norm (AA)	1		1		1		1		1	
		het (AG)	0.57 (0.49-0.68)	3.22×10^{-11}	0.62 (0.50-0.75)	1.68×10^{-6}	0.56 (0.43-0.73)	2.67×10^{-5}	0.54 (0.41-0.70)	5.76×10^{-6}	0.57 (0.38-0.86)	0.007
		hom (GG)	0.26 (0.11-0.62)	0.002	0.15 (0.03-0.64)	0.01	0.14 (0.01-1.04)	0.05	0.40 (0.12-1.32)	0.13	0.36 (0.04-2.66)	0.31
INPP4B	rs77389579	norm (GG)	1		1		1		1		1	
		het (GT)	0.61 (0.51-0.73)	7.11×10^{-8}	0.64 (0.51-0.80)	0.0001	0.56 (0.41-0.75)	0.0001	0.61 (0.45-0.81)	0.0007	0.51 (0.32-0.83)	0.006
		hom (TT)	0.25 (0.05-1.12)	0.07	0.22 (0.02-1.70)	0.14	0.41 (0.05-3.14)	0.39	-	-	-	-
ABO	rs876719*	non-O	1		1		1		1		1	
		O	0.74 (0.66-0.82)	3.09×10^{-8}	0.78 (0.69-0.89)	0.0003	0.67 (0.57-0.80)	4.13×10^{-6}	0.71 (0.60-0.83)	4.61×10^{-5}	0.67 (0.52-0.87)	0.002
ABO	rs8176746*	norm (CC)	1		1		1		1		1	
		het (AC)	1.35 (1.19-1.52)	9.85×10^{-7}	1.19 (1.03-1.39)	0.01	1.14 (1.17-1.70)	0.0002	1.24 (1.03-1.50)	0.02	1.26 (0.94-1.68)	0.11
		hom (AA)	1.79 (1.29-2.49)	0.0004	1.46 (0.97-2.20)	0.06	2.13 (1.35-3.36)	0.001	2.37 (1.53-3.65)	9.01×10^{-5}	1.61 (0.76-3.41)	0.20
HBB	rs334	norm (AA)	1		1		1		1		1	
		het (AT)	0.15 (0.11-0.20)	2.98×10^{-39}	0.14 (0.09-0.21)	2.01×10^{-23}	0.07 (0.04-0.15)	5.28×10^{-14}	0.11 (0.06-0.19)	1.58×10^{-14}	0.26 (0.14-0.48)	2.07×10^{-5}
		hom (TT)	0.57 (0.28-1.14)	0.11	0.77 (0.35-1.69)	0.51	1.32 (0.60-2.90)	0.48	1.14 (0.50-2.61)	0.74	1.31 (0.39-4.33)	0.65
RPS6KL1	rs3742785	norm (CC)	1		1		1		1		1	
		het (AC)	1.20 (1.07-1.34)	0.001	1.16 (1.01-1.34)	0.03	1.37 (1.15-1.64)	0.0004	1.04 (0.87-1.24)	0.62	0.94 (0.71-1.23)	0.66
		hom (AA)	0.99 (0.81-1.20)	0.93	0.88 (0.69-1.14)	0.35	1.20 (0.89-1.61)	0.2	0.86 (0.63-1.18)	0.37	0.70 (0.42-1.17)	0.18
HBA	$\alpha^{3.7}$-thalassaemia	norm	1		1		1		1		1	
		het	0.83 (0.70-0.93)	0.002	0.89 (0.77-1.02)	0.11	0.75 (0.63-0.90)	0.002	0.79 (0.66-0.94)	0.01	0.74 (0.56-0.97)	0.03
		hom	0.66 (0.55-0.78)	1.70×10^{-6}	0.65 (0.52-0.80)	9.68×10^{-5}	0.50 (0.38-0.67)	4.12×10^{-6}	0.61 (0.47-0.81)	0.0004	0.46 (0.29-0.73)	0.0009
CD40LG§	rs3092945	norm (TT)	1		1		1		1		1	
		het (CT)	1.12 (0.95-1.13)	0.14	0.98 (0.81-1.20)	0.92	1.18 (0.92-1.51)	0.16	1.28 (1.00-1.64)	0.04	1.30 (0.88-1.92)	0.18
		hom (CC)	1.31 (1.13-1.53)	0.0003	1.32 (1.09-1.59)	0.003	1.37 (1.09-1.72)	0.006	1.07 (0.84-1.37)	0.55	1.00 (0.68-1.46)	0.98
G6PD§	rs1050828	norm (CC)	1		1		1		1		1	
		het (CT)	0.82 (0.69-0.96)	0.01	0.79 (0.64-0.97)	0.02	0.80 (0.61-1.04)	0.1	0.88 (0.68-1.13)	0.33	0.60 (0.38-0.94)	0.02
		hom (TT)	1.10 (0.93-1.30)	0.25	0.94 (0.76-1.17)	0.63	1.58 (1.24-2.00)	0.0001	0.92 (0.70-1.21)	0.58	0.69 (0.44-1.09)	0.11

* The rs8176719 polymorphism identifies blood-group O in the homozygous deletion form. The derived allele of rs8176746 identifies blood-group B. †Analyses are adjusted for ethnic group, gender and rs334 genotype. Chr: chromosome; MAF; minor allele frequency; OR: Odds Ratio (95% Confidence Interval); P: p-value. Individuals with multiple phenotypes were included in each of those groups for analysis. §Hemizygous males are coded as homozygotes.

Table S8: Tests of association by specific genotypes for the most significant loci in the case-control study.

Gene	SNPID	Alleles (ancestral/ derived)	Chr	DAF Controls	No. of genotype in controls	Sample	Case Phenotype	DAF Cases	No. of genotypes in cases	MO	Unadjusted				Adjusted[§]			
											OR	LCL	UCL	P	OR	LCL	UCL	P
AJAP1	rs113788643	A/G	1	0.08	3940	All	CM only	0.08	670	R	1.43	0.58	3.51	0.4556	1.55	0.61	3.94	0.3815
AJAP1	rs6674631	G/A	1	0.13	3928	All	CM only	0.11	666	R	0.49	0.23	1.08	0.0535	0.55	0.25	1.22	0.0571
ATP2B4	rs10900585	G/T	1	0.34	3762	All	CM only	0.34	666	R	1.20	0.90	1.60	0.2112	1.06	0.70	1.59	0.1700
ATP2B4	rs1541255	A/G	1	0.33	3933	All	CM only	0.32	665	R	0.80	0.60	1.06	0.1141	0.81	0.60	1.09	0.0951
ATP2B4	rs3753036	G/A	1	0.04	3939	All	CM only	0.05	668	R	2.46	0.46	13.03	0.3241	1.08	0.80	1.46	0.6336
ATP2B4	rs4951074	G/A	1	0.32	3934	All	CM only	0.31	669	R	0.87	0.65	1.17	0.3565	1.12	0.62	2.03	0.33082
ATP2B4	rs55868763	G/C	1	0.33	3931	All	CM only	0.32	667	R	0.80	0.60	1.07	0.1275	0.82	0.60	1.12	0.1091
CR1	rs17047660	A/G	1	0.16	3923	All	CM only	0.19	659	A	1.18	1.01	1.37	0.0431	1.17	0.99	1.37	0.051
CR1	rs17047661	G/A	1	0.32	3934	All	CM only	0.34	655	A	1.09	0.96	1.24	0.1812	1.18	0.89	1.57	0.1841
DARC	rs2814778	G/A	1	0.00	3944	All	CM only	0.00	583	H	0.71	0.16	3.19	0.6403	0.67	0.15	3.04	0.63892
GBP7	rs1803632	G/C	1	0.49	3942	All	CM only	0.50	662	H	1.03	0.87	1.22	0.7072	1.05	0.86	1.28	0.6713
IL10	rs1800890	A/T	1	0.24	3945	All	CM only	0.22	662	R	0.66	0.44	0.99	0.0372	0.66	0.43	1.00	0.0369
IL10	rs1800896	T/C	1	0.38	3943	All	CM only	0.37	660	H	1.11	0.94	1.31	0.2197	1.10	0.93	1.31	0.2420
IL10	rs3024500	G/A	1	0.45	3928	All	CM only	0.48	661	R	1.16	0.95	1.41	0.1518	1.16	0.94	1.42	0.17422
LPHN2	rs146428334	A/G	1	0.00	3930	All	CM only	0.01	669	H	1.76	0.83	3.75	0.1603	1.87	0.87	4.02	0.1530
LPHN2	rs4650365	T/C	1	0.20	3936	All	CM only	0.19	666	H	0.79	0.66	0.95	0.0107	0.79	0.65	0.95	0.00521
LPHN2	rs72933304	C/A	1	0.09	3942	All	CM only	0.07	669	R	0.19	0.03	1.41	0.0339	0.20	0.03	1.47	0.0350
LPHN2	rs72933310	T/C	1	0.09	3927	All	CM only	0.08	669	A	0.87	0.70	1.07	0.1792	0.90	0.72	1.12	0.18514
LPHN2	rs72933350	T/C	1	0.05	3940	All	CM only	0.04	669	A	0.74	0.55	1.00	0.0447	0.79	0.58	1.08	0.0529
IL1A	rs17561	G/T	2	0.16	3938	All	CM only	0.18	661	R	1.32	0.82	2.12	0.2705	1.41	0.86	2.31	0.2744
IL1B	rs1143634	C/T	2	0.11	3941	All	CM only	0.13	660	R	1.25	0.64	2.44	0.5140	1.40	0.71	2.77	0.5136
LAPTM4A	rs973128	C/G	2	0.44	3885	All	CM only	0.44	661	D	0.99	0.82	1.18	0.8719	0.93	0.78	1.11	0.7362
LOC727982	rs10188961	A/G	2	0.40	3934	All	CM only	0.40	667	D	1.09	0.92	1.30	0.3197	1.13	0.94	1.35	0.3328
LOC727982	rs1371474	T/C	2	0.27	3933	All	CM only	0.29	669	H	1.29	1.09	1.53	0.0031	1.33	1.12	1.58	0.0045
LOC727982	rs1371478	C/T	2	0.27	3909	All	CM only	0.29	668	H	1.30	1.10	1.54	0.0022	1.34	1.13	1.60	0.0033
SDC1	rs11899121	C/G	2	0.47	3921	All	CM only	0.49	666	D	1.15	0.95	1.39	0.1534	1.15	0.95	1.40	0.17548
ZSWIM2	rs144778284	C/T	2	0.00	3937	All	CM only	0.00	669	H	0.21	0.03	1.56	0.0507	0.23	0.03	1.73	0.0518
ZSWIM2	rs4316902	A/G	2	0.18	3910	All	CM only	0.18	667	H	1.04	0.87	1.25	0.6799	1.07	0.89	1.29	0.65552
ARL14	rs74954675	A/C	3	0.08	3907	All	CM only	0.10	662	H	1.32	1.06	1.64	0.0147	1.37	1.10	1.72	0.0109
ARL14	rs75731597	A/C	3	0.08	3491	All	CM only	0.11	657	H	1.34	1.08	1.66	0.0101	1.39	1.11	1.74	0.01012
ARL14	rs76033371	A/G	3	0.09	3939	All	CM only	0.11	665	H	1.25	1.01	1.55	0.0412	1.30	1.04	1.62	0.0377
B3GALNT1	rs12107243	G/C	3	0.49	3934	All	CM only	0.49	668	D	0.94	0.77	1.13	0.4921	0.98	0.86	1.11	0.49661
IL17RD	rs6780995	A/G	3	0.41	3939	All	CM only	0.39	660	A	0.92	0.82	1.04	0.1948	0.92	0.82	1.04	0.2194
IL17RE	rs708567	A/G	3	0.50	3881	All	CM only	0.49	660	H	0.98	0.83	1.16	0.8002	0.96	0.81	1.14	0.75464
TLR9	rs187084	T/C	3	0.33	3933	All	CM only	0.31	584	A	0.90	0.79	1.02	0.1075	0.86	0.75	0.99	0.1035
FREM3	rs149914432	A/C	4	0.10	3915	All	CM only	0.05	661	A	0.53	0.41	0.68	7.7x10^{-08}	0.54	0.43	0.70	9.7x10^{-08}
FREM3	rs184895969	C/T	4	0.00	3940	All	CM only	0.00	669	H	0.66	0.23	1.89	0.4161	0.83	0.25	2.70	0.4234
FREM3	rs184908374	G/A	4	0.00	3943	All	CM only	0.00	669	H	0.82	0.28	2.36	0.7000	1.02	0.31	3.39	0.71109
FREM3	rs186790584	A/T	4	0.10	3941	All	CM only	0.05	668	A	0.52	0.41	0.67	6.1x10^{-08}	0.59	0.45	0.8	7.0x10^{-08}
FREM3	rs186873296	A/G	4	0.10	3926	All	CM only	0.05	667	A	0.51	0.39	0.66	2.6x10^{-08}	0.52	0.41	0.67	3.8x10^{-08}
GAB1	rs7663712	A/G	4	0.02	3938	All	CM only	0.03	668	R	-	-	-	-	-	-	-	-
INPP4B	rs13103597	C/T	4	0.27	3925	All	CM only	0.22	668	A	0.76	0.66	0.88	0.0001	0.81	0.69	0.96	0.00016
INPP4B	rs77389579	G/T	4	0.07	3937	All	CM only	0.05	663	A	0.63	0.48	0.83	0.0005	0.69	0.52	0.9	0.0005
TLR1	rs4833095	C/T	4	0.09	3921	All	CM only	0.10	585	R	1.43	0.62	3.29	0.4185	1.31	0.56	3.08	0.41949
TLR6	rs5743809	T/C	4	0.06	3884	All	CM only	0.06	558	R	0.46	0.06	3.51	0.3999	0.45	0.06	3.51	0.3992
TLR6	rs5743810	C/T	4	0.02	3936	All	CM only	0.01	587	A	0.76	0.44	1.32	0.3147	0.61	0.34	1.09	0.31328
USP38	rs28459062	T/C	4	0.16	3931	All	CM only	0.20	669	A	1.26	1.09	1.47	0.0024	1.29	1.08	1.55	0.0029
USP38	rs4266246	C/T	4	0.22	3928	All	CM only	0.22	667	H	1.12	0.94	1.33	0.2077	1.04	0.87	1.25	0.18183
C6	rs1801033	A/C	5	0.47	3927	All	CM only	0.47	584	H	0.97	0.82	1.16	0.7761	1.00	0.83	1.19	0.7991
IL13	rs20541	C/T	5	0.22	3796	All	CM only	0.21	557	R	0.73	0.46	1.14	0.1514	0.70	0.44	1.11	0.17
IRF	rs2706384	C/A	5	0.38	3819	All	CM only	0.36	572	H	0.90	0.75	1.08	0.2449	0.90	0.75	1.08	0.2643
CTL4	rs2242665	A/G	6	0.26	3853	All	CM only	0.26	575	R	0.91	0.64	1.31	0.6257	0.84	0.58	1.22	0.64253
GABBR1	rs192151845	G/A	6	0.01	3943	All	CM only	0.01	666	H	1.07	0.54	2.12	0.8487	1.12	0.56	2.25	0.8349
HCG4	rs114980857	C/T	6	0.21	3843	All	CM only	0.24	667	D	1.21	1.02	1.43	0.0269	1.19	1.00	1.42	0.026352
HSPA1B	rs6457452	C/T	6	0.19	3917	All	CM only	0.18	658	D	0.89	0.74	1.06	0.1928	0.89	0.74	1.07	0.1974
IL20RA	rs1555498	C/T	6	0.46	3947	All	CM only	0.48	661	H	1.07	0.91	1.27	0.4116	1.09	0.92	1.30	0.40271
LTA	rs2239704	G/T	6	0.12	3896	All	CM only	0.13	653	H	1.09	0.89	1.33	0.3982	1.04	0.85	1.28	0.4347
LTA	rs909253	T/C	6	0.49	3921	All	CM only	0.48	661	H	0.90	0.76	1.06	0.2128	0.92	0.77	1.09	0.21919
PLEKHG1	rs114726617	G/A	6	0.04	3940	All	CM only	0.04	669	A	1.13	0.84	1.51	0.4271	1.17	0.86	1.60	0.4339
PLEKHG1	rs141555199	C/G	6	0.02	3943	All	CM only	0.02	669	R	-	-	-	-	-	-	-	-
PLEKHG1	rs142712208	G/A	6	0.03	3927	All	CM only	0.03	669	D	1.10	0.78	1.54	0.5876	1.19	0.85	1.66	0.6292
PLEKHG1	rs15116938	C/T	6	0.03	3939	All	CM only	0.03	669	A	1.04	0.75	1.44	0.8199	1.13	0.81	1.58	0.86408
PLEKHG1	rs151293197	C/G	6	0.03	3940	All	CM only	0.03	669	R	2.15	0.42	10.94	0.3883	2.83	0.53	15.15	0.3812
PLEKHG1	rs2131263	C/T	6	0.04	3938	All	CM only	0.04	669	A	1.13	0.84	1.51	0.4282	1.17	0.86	1.60	0.435
PLEKHG1	rs55958968	A/G	6	0.05	3932	All	CM only	0.05	669	R	-	-	-	-	-	-	-	-
PLEKHG1	rs76924464	G/A	6	0.03	3940	All	CM only	0.03	669	R	2.15	0.42	10.95	0.3879	2.82	0.53	15.09	0.38075
PLEKHG1	rs79100774	G/C	6	0.03	3939	All	CM only	0.03	670	R	2.15	0.42	10.94	0.3885	2.83	0.53	15.15	0.3813
SNORD48	rs116288147	C/T	6	0.24	3896	All	CM only	0.24	659	H	0.96	0.81	1.14	0.6533	0.97	0.81	1.17	0.75804
TNF	rs1799964	T/C	6	0.27	3938	All	CM only	0.28	654	R	1.11	0.82	1.51	0.5068	1.14	0.83	1.57	0.5155
TNF	rs1800629	G/A	6	0.09	3940	All	CM only	0.08	663	A	0.91	0.73	1.12	0.3730	0.91	0.73	1.13	0.38746
TNF	rs1800750	G/A	6	0.05	3868	All	CM only	0.05	423	R	1.08	0.25	4.70	0.9215	0.97	0.69	1.39	0.9179
TNF	rs3093662	A/G	6	0.11	3936	All	CM only	0.12	659	H	1.10	0.90	1.35	0.3498	1.09	0.89	1.35	0.38042
TNF	rs361525	G/A	6	0.07	3948	All	CM only	0.07	658	R	0.84	0.25	2.83	0.7698	0.99	0.29	3.40	0.7682
CD36	rs3211938	T/G	7	0.10	3942	All	CM only	0.09	656	R	0.64	0.23	1.82	0.3783	0.72	0.25	2.07	0.3757
NOD1	rs2075820	G/A	7	0.38	3941	All	CM only	0.38	587	H	1.03	0.86	1.23	0.7513	1.04	0.87	1.25	0.7894
ABO	rs56390333	G/A	9	0.00	3942	All	CM only	0.00	670	H	2.20	0.56	8.65	0.2855	2.34	0.58	9.44	0.28169
ABO	rs8176719*	O vs non-O	9	0.26	3888	All	CM only	0.29	663	R	0.65	0.48	0.81	0.0040	0.66	0.49	0.83	0.0040
ABO	rs8176746*	C/A	9	0.13	3924	All	CM only	0.15	661	A	1.17	0.97	1.42	0.1069	1.13	0.90	1.42	0.13497
ABO	rs8176750	I/D	9	0.05	3923	All	CM only	0.05	667	R	-	-	-	-	-	-	-	-
TLR4	rs4986790	A/G	9	0.04	3929	All	CM only	0.04	581	A	0.83	0.60	1.15	0.2472	0.81	0.58	1.14	0.24062
TLR4	rs4986791	C/T	9	0.01	3937	All	CM only	0.00	583	H	0.73	0.29	1.86	0.4983	0.81	0.32	2.08	0.4942
MKI67	rs11016116	A/G	10	0.02	3944	All	CM only	0.03	669	R	4.13	0.90	18.90	0.0858	4.96	1.03	23.91	0.084249
MKI67	rs115947774	A/G	10	0.00	3931	All	CM only	0.00	665	H	0.69	0.24	1.98	0.4699	0.69	0.24	2.01	0.4756
MKI67	rs148494166	T/C	10	0.00	3913	All	CM only	0.00	651	H	2.44	0.93	6.40	0.0899	2.08	0.78	5.55	0.086194
HBB	rs334	A/T	11	0.08	3949	All	CM only	0.02	660	D	0.18	0.11	0.28	6.1x10^{-22}	0.17	0.11	0.27	7.5x10^{-22}
RRM1	rs116472045	A/G	11	0.02	3939	All	CM only	0.01	667	R	-	-	-	-	-	-	-	-
RTN3	rs542998	T/C	11	0.43	3929	All	CM only	0.41	583	R	0.75	0.59	0.96	0.0179	0.79	0.62	1.01	0.0193

TRIM5	rs7935564	G/A	11	0.44	3871	All	CM only	0.45	585	H	0.91	0.76	1.08	0.2738	0.90	0.75	1.08	0.29658
CAND1	rs10459266	C/A	12	0.21	3938	All	CM only	0.21	668	R	1.19	0.82	1.73	0.3615	1.18	0.81	1.72	0.3127
CAND1	rs12307123	C/T	12	0.23	3933	All	CM only	0.24	669	A	1.09	0.95	1.25	0.2477	1.07	0.93	1.23	0.25064
CAND1	rs1566830	G/T	12	0.26	3940	All	CM only	0.27	669	A	1.10	0.96	1.25	0.1684	1.08	0.94	1.24	0.1629
GRIP1	rs1394263	T/C	12	0.26	3914	All	CM only	0.27	666	A	1.10	0.97	1.26	0.1508	1.08	0.95	1.24	0.14575
IL22	rs1012356	A/T	12	0.49	3940	All	CM only	0.47	659	D	0.91	0.75	1.09	0.2982	0.93	0.77	1.12	0.2899
IL22	rs2227478	A/G	12	0.38	3939	All	CM only	0.39	661	H	1.02	0.86	1.20	0.8511	1.07	0.90	1.27	0.84254
IL22	rs2227485	G/A	12	0.42	3928	All	CM only	0.40	659	A	0.93	0.83	1.05	0.2441	0.93	0.82	1.05	0.2562
IL22	rs2227491	C/T	12	0.40	3870	All	CM only	0.43	646	A	1.11	0.98	1.25	0.0932	1.09	0.97	1.24	0.090368
IL22	rs2227507	T/C	12	0.04	3948	All	CM only	0.04	663	R	1.79	0.47	6.82	0.4140	1.61	0.41	6.25	0.4152
TPTE2	rs182873742	C/G	13	0.00	3940	All	CM only	0.00	665	H	-	-	-	-	-	-	-	-
RPS6KL1	rs3742785	C/A	14	0.29	3714	All	CM only	0.30	632	H	1.20	1.01	1.43	0.0366	1.17	0.98	1.40	0.0376
SPTB	rs229587	T/C	14	0.34	3872	All	CM only	0.33	642	R	0.78	0.58	1.03	0.0712	0.80	0.60	1.07	0.080607
YLPM1	rs10139016	C/T	14	0.25	3937	All	CM only	0.28	668	D	1.27	1.07	1.50	0.0050	1.24	1.04	1.47	0.0049
ADCY9	rs10775349	C/G	16	0.20	3941	All	CM only	0.20	587	R	0.76	0.45	1.27	0.2732	0.81	0.48	1.36	0.27141
ADCY9	rs2230739	A/G	16	0.09	3944	All	CM only	0.07	587	H	0.77	0.60	1.01	0.0492	0.76	0.58	0.99	0.0499
HBA	$\alpha^{-3.7}$-thalassaemia	I/D	16	0.41	3946	All	CM only	0.37	646	A	0.85	0.75	0.97	0.0126	0.76	0.59	0.99	0.022178
IL4	rs2243250	T/C	16	0.20	3902	All	CM only	0.23	655	D	1.19	1.00	1.41	0.0495	1.17	0.98	1.39	0.0439
IL4R	rs1805015	T/C	16	0.42	3927	All	CM only	0.45	661	D	1.20	1.00	1.44	0.0437	1.24	1.02	1.49	0.040194
ADORA2B	rs2535611	T/C	17	0.10	3911	All	CM only	0.10	586	R	0.28	0.07	1.16	0.0330	0.25	0.06	1.06	0.0325
NOS2	rs1800482	G/C	17	0.08	3922	All	CM only	0.07	659	H	0.90	0.71	1.15	0.4109	0.89	0.69	1.14	0.36808
NOS2	rs2297518	G/A	17	0.14	3910	All	CM only	0.12	582	A	0.86	0.71	1.04	0.1051	0.86	0.71	1.04	0.1030
NOS2	rs8078340	C/T	17	0.21	3948	All	CM only	0.20	662	R	0.70	0.43	1.14	0.1306	0.68	0.42	1.11	0.13073
NOS2	rs9282799	C/T	17	0.03	3949	All	CM only	0.04	659	H	1.44	1.03	2.00	0.0378	1.36	0.95	1.93	0.0484
TBX2	rs73991577	A/G	17	0.00	3937	All	CM only	0.00	669	H	0.79	0.23	2.69	0.6962	0.81	0.23	2.83	0.70518
EMR1	rs373533	G/T	19	0.46	3863	All	CM only	0.50	584	A	1.13	1.00	1.28	0.0503	1.12	0.99	1.28	0.0577
EMR1	rs461645	C/T	19	0.47	3933	All	CM only	0.50	587	A	1.12	0.99	1.27	0.0728	1.11	0.98	1.26	0.082877
ICAM	rs5498	A/G	19	0.11	3908	All	CM only	0.12	652	H	0.99	0.80	1.21	0.9097	0.96	0.78	1.19	0.9401
GNAS	rs8386	C/T	20	0.19	3944	All	CM only	0.20	660	R	1.65	1.11	2.45	0.0186	1.65	1.10	2.48	0.018891
DERL3	rs1128127	G/A	22	0.50	3911	All	CM only	0.48	660	D	0.93	0.77	1.12	0.4317	0.91	0.75	1.11	0.4094
CD40LG	rs1126535	T/C	X	0.15	3934	M+F	CM only	0.17	662	R	1.16	0.87	1.55	0.3039	1.18	0.87	1.58	0.30855
CD40LG	rs1126535	T/C	X	0.16	1947	F	CM only	0.15	330	D	0.92	0.71	1.20	0.5552	0.93	0.73	1.22	0.5652
CD40LG	rs1126535	C/T	X	0.85	1987	M	CM only	0.82	332	HM	0.82	0.60	1.12	0.2124	0.84	0.62	1.22	0.2324
CD40LG	rs3092945	T/C	X	0.21	3927	M+F	CM only	0.26	663	R	1.47	1.17	1.85	0.0013	1.52	1.20	1.92	0.0013
CD40LG	rs3092945	T/C	X	0.21	1942	F	CM only	0.25	331	R	2.27	1.46	3.52	0.0005	2.28	1.48	3.56	0.0005
CD40LG	rs3092945	C/T	X	0.78	1985	M	CM only	0.73	332	HM	0.78	0.60	1.02	0.0736	0.79	0.63	1.09	0.0836
G6PD	rs1050828	C/T	X	0.19	3940	M+F	CM only	0.17	661	D	0.83	0.69	1.02	0.0697	0.85	0.69	1.04	0.069691
G6PD	rs1050828	C/T	X	0.20	1951	F	CM only	0.18	329	H	0.79	0.61	1.02	0.0687	0.80	0.64	1.03	0.0787
G6PD	rs1050828	C/T	X	0.19	1989	M	CM only	0.16	332	HM	0.84	0.61	1.15	0.2796	0.86	0.65	1.16	0.2996
G6PD	rs1050829	T/C	X	0.40	3928	M+F	CM only	0.41	660	H	0.83	0.66	1.05	0.1242	0.87	0.68	1.12	0.1188
G6PD	rs1050829	T/C	X	0.41	1952	F	CM only	0.42	329	H	0.83	0.65	1.05	0.1193	0.85	0.68	1.07	0.1293
G6PD	rs1050829	C/T	X	0.60	1976	M	CM only	0.60	331	HM	0.99	0.78	1.26	0.9445	0.99	0.81	1.28	0.9645

* The rs8176719 polymorphism identifies blood-group O in the homozygous deletion form. The derived allele of rs8176746 identifies blood-group B. Chr = chromosome; DAF = derived allele frequency; MO = genotype model [A – additive, D – dominant, H – heterozygote, HM – hemizygous males, R – recessive] with respect to the derived allele; OR = Odds Ratio; LCI = Lower Confidence Interval (95%); UCI = Upper Confidence Interval; P = p-value using a logistic regression model as described in methods. § ORs and p-values unadjusted and adjusted for ethnic group, gender and rs334 genotype. Only those individuals solely having CM were included in this analysis.

Table S9: Odds ratios for CM only for all polymorphisms investigated.

Gene	SNPID	Alleles (ancestral/ derived)	Chr	DAF Controls	No. of genotype in controls	Sample	Case Phenotype	DAF Cases	No. of genotypes in cases	M O	Unadjusted				Adjusted§			
											OR	LCL	UCL	P	OR	LCL	UCL	P
AJAP1	rs113788643	A/G	1	0.92	3940	All	SMA only	0.91	227	R	1.94	0.58	6.45	0.3201	2.00	0.60	6.69	0.3042
AJAP1	rs6674631	G/A	1	0.87	3928	All	SMA only	0.88	228	R	0.91	0.68	1.22	0.5193	0.63	0.20	2.04	0.4130
ATP2B4	rs10900585	G/T	1	0.66	3762	All	SMA only	0.66	226	H	1.16	0.89	1.52	0.2687	1.18	0.90	1.55	0.2251
ATP2B4	rs1541255	A/G	1	0.67	3933	All	SMA only	0.67	226	R	0.86	0.54	1.37	0.5178	0.85	0.53	1.34	0.4698
ATP2B4	rs3753036	G/A	1	0.96	3939	All	SMA only	0.94	228	H	1.46	0.96	2.23	0.0932	1.45	0.95	2.22	0.0993
ATP2B4	rs4951074	G/A	1	0.68	3934	All	SMA only	0.68	227	H	1.17	0.89	1.53	0.2518	1.18	0.90	1.55	0.2202
ATP2B4	rs55868763	G/C	1	0.67	3931	All	SMA only	0.68	225	R	0.83	0.52	1.33	0.4231	0.82	0.51	1.31	0.3877
CR1	rs17047660	A/G	1	0.84	3923	All	SMA only	0.86	230	A	0.85	0.65	1.12	0.2318	0.83	0.63	1.10	0.1810
CR1	rs17047661	G/A	1	0.68	3934	All	SMA only	0.67	229	R	1.16	0.76	1.77	0.4879	1.23	0.81	1.88	0.3447
DARC	rs2814778	G/A	1	1.00	3944	All	SMA only	1.00	207	A	1.47	0.19	11.28	0.7265	1.30	0.17	10.16	0.8073
GBP7	rs1803632	G/C	1	0.51	3942	All	SMA only	0.52	230	H	0.91	0.70	1.19	0.5015	0.91	0.69	1.18	0.4696
IL10	rs1800890	A/T	1	0.76	3945	All	SMA only	0.74	230	H	1.17	0.89	1.53	0.2694	1.20	0.91	1.57	0.2016
IL10	rs1800896	T/C	1	0.62	3943	All	SMA only	0.61	227	R	1.25	0.87	1.78	0.2372	1.28	0.90	1.84	0.1802
IL10	rs3024500	G/A	1	0.55	3928	All	SMA only	0.55	229	H	0.76	0.58	0.99	0.0401	0.75	0.57	0.98	0.0348
LPHN2	rs146428334	A/G	1	1.00	3930	All	SMA only	1.00	228	A	0.52	0.07	3.81	0.4754	0.58	0.08	4.29	0.5627
LPHN2	rs4650365	T/C	1	0.80	3936	All	SMA only	0.81	228	H	0.83	0.62	1.11	0.2109	0.82	0.61	1.10	0.1755
LPHN2	rs72933304	C/A	1	0.91	3942	All	SMA only	0.93	228	R	-	-	-	-	-	-	-	-
LPHN2	rs72933310	T/C	1	0.91	3927	All	SMA only	0.92	228	R	-	-	-	-	-	-	-	-
LPHN2	rs72933350	T/C	1	0.95	3940	All	SMA only	0.96	228	A	0.77	0.47	1.26	0.2768	0.73	0.44	1.20	0.1959
IL1A	rs17561	G/T	2	0.84	3938	All	SMA only	0.85	230	H	0.75	0.55	1.03	0.0697	0.73	0.53	1.01	0.0486
IL1B	rs1143634	C/T	2	0.89	3941	All	SMA only	0.88	230	R	1.77	0.70	4.47	0.2657	1.61	0.63	4.10	0.3464
LAPTM4A	rs973128	C/G	2	0.56	3885	All	SMA only	0.53	225	R	1.28	0.93	1.77	0.1417	1.27	0.92	1.75	0.1620
LOC727982	rs10188961	A/G	2	0.60	3934	All	SMA only	0.58	227	D	1.14	0.86	1.52	0.3559	1.19	0.89	1.58	0.2394
LOC727982	rs1371474	T/C	2	0.73	3933	All	SMA only	0.71	228	H	1.16	0.89	1.53	0.2753	1.19	0.91	1.57	0.2075
LOC727982	rs1371478	C/T	2	0.73	3909	All	SMA only	0.72	226	H	1.14	0.87	1.50	0.3360	1.17	0.89	1.54	0.2564
SDC1	rs11899121	C/G	2	0.53	3921	All	SMA only	0.53	226	H	1.05	0.80	1.37	0.7268	1.06	0.81	1.38	0.6948
ZSWIM2	rs144778284	C/T	2	1.00	3937	All	SMA only	1.00	228	A	1.65	0.38	7.08	0.5285	1.34	0.31	5.78	0.7062
ZSWIM2	rs4316902	A/G	2	0.82	3910	All	SMA only	0.79	226	A	1.20	0.95	1.51	0.1352	1.22	0.96	1.54	0.1087
ARL14	rs74954675	A/C	3	0.92	3907	All	SMA only	0.89	228	H	1.56	1.11	2.18	0.0131	1.46	1.04	2.05	0.0348
ARL14	rs75731597	A/C	3	0.92	3491	All	SMA only	0.90	221	H	1.40	0.99	1.99	0.0649	1.33	0.94	1.89	0.1191
ARL14	rs76033371	A/G	3	0.91	3939	All	SMA only	0.89	228	H	1.48	1.06	2.06	0.0248	1.38	0.99	1.93	0.0636
B3GALNT1	rs12107243	G/C	3	0.51	3934	All	SMA only	0.50	228	D	1.15	0.84	1.58	0.3725	1.13	0.82	1.55	0.4518
IL17RD	rs6780995	A/G	3	0.59	3939	All	SMA only	0.60	230	H	1.23	0.94	1.60	0.1339	1.23	0.94	1.61	0.1288
IL17RE	rs708567	A/G	3	0.50	3881	All	SMA only	0.53	229	R	0.79	0.57	1.09	0.1401	0.80	0.57	1.10	0.1633
TLR9	rs187084	T/C	3	0.67	3933	All	SMA only	0.67	208	R	0.94	0.60	1.46	0.7783	0.92	0.59	1.44	0.7131
FREM3	rs149914432	A/C	4	0.90	3915	All	SMA only	0.94	226	A	0.56	0.38	0.84	0.0024	0.56	0.38	0.84	0.0026
FREM3	rs184895969	C/T	4	1.00	3940	All	SMA only	1.00	228	A	1.12	0.27	4.69	0.8829	1.01	0.24	4.25	0.9932
FREM3	rs184908374	G/A	4	1.00	3943	All	SMA only	1.00	228	A	1.39	0.33	5.89	0.6719	1.23	0.29	5.27	0.7822
FREM3	rs186790584	A/T	4	0.90	3941	All	SMA only	0.95	228	A	0.54	0.36	0.82	0.0015	0.55	0.36	0.83	0.0018
FREM3	rs186873296	A/G	4	0.90	3926	All	SMA only	0.95	226	A	0.51	0.33	0.78	0.0006	0.51	0.33	0.78	0.0007
GAB1	rs7663712	A/G	4	0.98	3938	All	SMA only	0.98	228	A	0.79	0.40	1.55	0.4748	0.82	0.42	1.62	0.5562
INPP4B	rs13103597	C/T	4	0.73	3925	All	SMA only	0.77	228	D	0.83	0.66	1.03	0.0912	0.79	0.60	1.04	0.0849
INPP4B	rs77389579	G/T	4	0.93	3937	All	SMA only	0.95	228	A	0.65	0.41	1.01	0.0430	0.64	0.41	1.00	0.0383
TLR1	rs4833095	C/T	4	0.91	3921	All	SMA only	0.92	208	H	0.65	0.42	1.05	0.0643	0.69	0.45	1.06	0.0743
TLR6	rs5743809	T/C	4	0.94	3884	All	SMA only	0.96	200	H	0.65	0.37	1.13	0.1036	0.63	0.36	1.10	0.0822
TLR6	rs5743810	C/T	4	0.98	3936	All	SMA only	0.98	208	R	-	-	-	-	-	-	-	-
USP38	rs28459062	T/C	4	0.84	3931	All	SMA only	0.83	227	H	1.06	0.79	1.43	0.6870	1.06	0.79	1.44	0.6864
USP38	rs4266246	C/T	4	0.78	3928	All	SMA only	0.76	227	R	1.85	1.13	3.02	0.0227	1.89	1.15	3.09	0.0195
C6	rs1801033	A/C	5	0.53	3927	All	SMA only	0.53	207	H	0.94	0.71	1.24	0.6586	0.92	0.70	1.22	0.5694
IL13	rs20541	C/T	5	0.78	3796	All	SMA only	0.78	195	R	0.48	0.20	1.19	0.0763	0.46	0.19	1.13	0.0560
IRF	rs2706384	C/A	5	0.62	3819	All	SMA only	0.65	204	R	0.67	0.42	1.06	0.0695	0.68	0.43	1.08	0.0904
CTL4	rs2242665	A/G	6	0.74	3853	All	SMA only	0.78	204	D	0.69	0.52	0.92	0.0115	0.69	0.52	0.93	0.0131
GABBR1	rs192151845	G/A	6	0.99	3943	All	SMA only	1.00	227	A	0.31	0.04	2.23	0.1529	0.31	0.04	2.27	0.1612
HCG4	rs114980857	C/T	6	0.79	3843	All	SMA only	0.79	225	R	0.70	0.34	1.45	0.3171	0.73	0.35	1.49	0.3625
HSPA1B	rs6457452	C/T	6	0.81	3917	All	SMA only	0.84	230	A	0.79	0.61	1.02	0.0675	0.79	0.61	1.02	0.0586
IL20RA	rs1555498	C/T	6	0.54	3947	All	SMA only	0.48	229	R	1.22	1.01	1.47	0.0363	1.31	0.97	1.77	0.0824
LTA	rs2239704	G/T	6	0.88	3896	All	SMA only	0.90	223	R	-	-	-	-	-	-	-	-
LTA	rs909253	T/C	6	0.51	3921	All	SMA only	0.46	227	D	1.38	0.99	1.92	0.0502	1.42	1.02	1.98	0.0340
PLEKHG1	rs114726617	G/A	6	0.96	3940	All	SMA only	0.96	228	R	2.17	0.27	17.39	0.5100	2.09	0.26	16.98	0.5279
PLEKHG1	rs141555199	C/G	6	0.98	3943	All	SMA only	0.98	228	A	0.76	0.35	1.62	0.4582	0.71	0.33	1.52	0.3470
PLEKHG1	rs142712208	G/A	6	0.97	3927	All	SMA only	0.97	228	R	2.88	0.35	24.02	0.3888	3.10	0.37	26.17	0.3621
PLEKHG1	rs15116938	C/T	6	0.97	3939	All	SMA only	0.96	228	R	2.89	0.35	24.09	0.3876	3.11	0.37	26.23	0.3611
PLEKHG1	rs151293197	C/G	6	0.97	3940	All	SMA only	0.97	228	R	2.89	0.35	24.09	0.3875	3.11	0.37	26.24	0.3610
PLEKHG1	rs2131263	C/T	6	0.96	3938	All	SMA only	0.96	228	R	2.16	0.27	17.38	0.5103	2.09	0.26	16.97	0.5281
PLEKHG1	rs55958968	A/G	6	0.95	3932	All	SMA only	0.94	228	A	1.23	0.83	1.81	0.3141	1.28	0.87	1.90	0.2221
PLEKHG1	rs76924464	G/A	6	0.97	3940	All	SMA only	0.97	228	R	2.89	0.35	24.09	0.3875	3.11	0.37	26.25	0.3609
PLEKHG1	rs79100774	G/C	6	0.97	3939	All	SMA only	0.97	228	R	2.89	0.35	24.09	0.3876	3.11	0.37	26.23	0.3612
SNORD48	rs116288147	C/T	6	0.76	3896	All	SMA only	0.79	227	A	0.85	0.67	1.07	0.1539	0.82	0.65	1.03	0.0855
TNF	rs1799964	T/C	6	0.73	3938	All	SMA only	0.71	228	R	1.05	0.86	1.30	0.6195	1.13	0.70	1.84	0.6212
TNF	rs1800629	G/A	6	0.91	3940	All	SMA only	0.90	228	R	1.74	0.53	5.74	0.3991	1.81	0.54	5.99	0.3711
TNF	rs1800750	G/A	6	0.95	3868	All	SMA only	0.92	125	H	1.62	0.98	2.66	0.0734	1.71	1.04	2.83	0.0474
TNF	rs3093662	A/G	6	0.89	3936	All	SMA only	0.90	227	H	0.79	0.55	1.14	0.1984	0.77	0.53	1.11	0.1531
TNF	rs361525	G/A	6	0.93	3948	All	SMA only	0.93	229	R	-	-	-	-	-	-	-	-
CD36	rs3211938	T/G	7	0.90	3942	All	SMA only	0.91	230	R	0.42	0.06	3.03	0.3166	0.41	0.06	3.04	0.3167
NOD1	rs2075820	G/A	7	0.62	3941	All	SMA only	0.63	208	H	0.90	0.68	1.19	0.4571	0.92	0.69	1.22	0.5493
ABO	rs56390333	G/A	9	1.00	3942	All	SMA only	1.00	228	A	-	-	-	-	-	-	-	-
ABO	rs8176719*	O vs non-O	9	0.74	3888	All	SMA only	0.66	229	D	0.62	0.42	0.81	3.0×10^{-06}	0.63	0.45	0.84	3.9×10^{-06}
ABO	rs8176746*	C/A	9	0.87	3924	All	SMA only	0.79	229	A	1.69	1.34	2.14	0.0000	1.66	1.31	2.10	0.0001
ABO	rs8176750	I/D	9	0.95	3923	All	SMA only	0.95	228	A	1.11	0.74	1.69	0.6148	1.15	0.76	1.75	0.5118
TLR4	rs4986790	A/G	9	0.96	3929	All	SMA only	0.98	207	A	0.56	0.30	1.04	0.0448	0.56	0.30	1.05	0.0486
TLR4	rs4986791	C/T	9	0.99	3937	All	SMA only	0.99	204	A	1.55	0.56	4.35	0.4280	1.63	0.58	4.58	0.3851
MKI67	rs11016116	A/G	10	0.98	3944	All	SMA only	0.98	228	R	-	-	-	-	-	-	-	-

MKI67	rs115947774	A/G	10	1.00	3931	All	SMA only	1.00	226	A	0.67	0.09	4.94	0.6734	0.57	0.08	4.21	0.5448
MKI67	rs148494166	T/C	10	1.00	3913	All	SMA only	1.00	214	A	-	-	-	-	-	-	-	-
HBB	rs334	A/T	11	0.92	3949	All	SMA only	0.98	228	H	0.10	0.04	0.27	9.68×10^{-12}	0.10	0.04	0.28	3.87×10^{-11}
RRM1	rs116472045	A/G	11	0.98	3939	All	SMA only	0.97	228	H	1.98	1.10	3.56	0.0368	2.01	1.11	3.64	0.0334
RTN3	rs542998	T/C	11	0.57	3929	All	SMA only	0.51	201	D	1.28	1.05	1.56	0.0164	1.44	1.04	2.00	0.0234
TRIM5	rs7935564	G/A	11	0.56	3871	All	SMA only	0.51	206	D	1.47	1.06	2.05	0.0177	1.44	1.03	2.00	0.0269
CAND1	rs10459266	C/A	12	0.79	3938	All	SMA only	0.76	228	H	1.31	1.00	1.73	0.0547	1.32	1.00	1.74	0.0522
CAND1	rs12307123	C/T	12	0.77	3933	All	SMA only	0.78	228	H	0.83	0.62	1.11	0.2087	0.84	0.63	1.12	0.2402
CAND1	rs1566830	G/T	12	0.74	3940	All	SMA only	0.74	227	H	0.84	0.64	1.11	0.2251	0.84	0.64	1.12	0.2338
GRIP1	rs1394263	T/C	12	0.74	3914	All	SMA only	0.74	225	H	0.83	0.62	1.10	0.1805	0.83	0.62	1.10	0.1887
IL22	rs1012356	A/T	12	0.51	3940	All	SMA only	0.50	229	D	1.07	0.82	1.40	0.6041	1.10	0.80	1.50	0.5615
IL22	rs2227478	A/G	12	0.62	3939	All	SMA only	0.60	230	D	1.19	0.90	1.57	0.2291	1.14	0.86	1.51	0.3608
IL22	rs2227485	G/A	12	0.58	3928	All	SMA only	0.60	226	R	0.67	0.45	1.00	0.0380	0.69	0.46	1.03	0.0602
IL22	rs2227491	C/T	12	0.60	3870	All	SMA only	0.62	227	R	0.69	0.46	1.05	0.0695	0.68	0.45	1.03	0.0549
IL22	rs2227507	T/C	12	0.96	3948	All	SMA only	0.96	230	R	-	-	-	-	-	-	-	-
TPTE2	rs182873742	C/G	13	1.00	3940	All	SMA only	1.00	228	A	-	-	-	-	-	-	-	-
RPS6KL1	rs3742785	C/A	14	0.71	3714	All	SMA only	0.66	222	D	1.38	1.05	1.81	0.0212	1.40	1.06	1.84	0.0156
SPTB	rs229587	T/C	14	0.66	3872	All	SMA only	0.66	210	H	0.90	0.68	1.20	0.4737	0.89	0.67	1.18	0.4127
YLPM1	rs10139016	C/T	14	0.75	3937	All	SMA only	0.70	227	D	1.33	1.02	1.74	0.0381	1.36	1.04	1.78	0.0243
ADCY9	rs10775349	C/G	16	0.80	3941	All	SMA only	0.79	207	H	1.08	0.81	1.45	0.6033	1.08	0.81	1.46	0.5945
ADCY9	rs2230739	A/G	16	0.91	3944	All	SMA only	0.91	208	H	0.61	0.08	4.48	0.5993	1.08	0.75	1.58	0.6750
HBA	$\alpha^{-3.7}$-thalassaemia	I/D	16	0.59	3946	All	SMA only	0.67	218	A	0.71	0.57	0.87	0.0009	0.70	0.57	0.86	0.0007
IL4	rs2243250	T/C	16	0.80	3902	All	SMA only	0.77	224	H	1.31	1.00	1.72	0.0560	1.30	0.98	1.71	0.0716
IL4R	rs1805015	T/C	16	0.58	3927	All	SMA only	0.59	229	D	0.91	0.69	1.20	0.5018	0.90	0.68	1.19	0.4620
ADORA2B	rs2535611	T/C	17	0.90	3911	All	SMA only	0.90	207	R	0.45	0.06	3.27	0.3649	0.40	0.05	2.94	0.2958
NOS2	rs1800482	G/C	17	0.92	3922	All	SMA only	0.91	229	H	1.18	0.83	1.69	0.3695	1.20	0.84	1.72	0.3328
NOS2	rs2297518	G/A	17	0.86	3910	All	SMA only	0.85	205	A	1.09	0.83	1.44	0.5371	1.07	0.81	1.42	0.6169
NOS2	rs8078340	C/T	17	0.79	3948	All	SMA only	0.80	230	H	0.82	0.61	1.10	0.1803	0.83	0.62	1.11	0.2009
NOS2	rs9282799	C/T	17	0.97	3949	All	SMA only	0.97	229	H	1.34	0.78	2.30	0.3142	1.29	0.75	2.22	0.3804
TBX2	rs73991577	A/G	17	1.00	3937	All	SMA only	1.00	228	A	0.91	0.12	6.82	0.9245	0.78	0.10	5.91	0.8061
EMR1	rs373533	G/T	19	0.54	3863	All	SMA only	0.51	207	R	1.19	0.86	1.64	0.3115	1.15	0.83	1.59	0.4097
EMR1	rs461645	C/T	19	0.53	3933	All	SMA only	0.51	208	R	1.19	0.86	1.64	0.3071	1.15	0.83	1.59	0.4081
ICAM	rs5498	A/G	19	0.89	3908	All	SMA only	0.88	226	R	1.91	0.81	4.49	0.1707	1.73	0.73	4.09	0.2410
GNAS	rs8386	C/T	20	0.81	3944	All	SMA only	0.79	230	R	1.68	0.92	3.09	0.1159	1.69	0.92	3.10	0.1153
DERL3	rs1128127	G/A	22	0.50	3911	All	SMA only	0.57	228	A	0.78	0.64	0.94	0.0076	0.79	0.65	0.96	0.0154
CD40LG	rs1126535	T/C	x	0.85	3934	M+F	SMA only	0.86	229	H	0.60	0.37	0.98	0.0280	0.68	0.41	1.14	0.1355
CD40LG	rs1126535	T/C	x	0.85	3934	F	SMA only	0.86	229	H	0.70	0.42	1.17	0.1605	0.73	0.47	1.14	0.1341
CD40LG	rs1126535	T/C	x	0.85	3934	M	SMA only	0.86	229	HM	0.93	0.57	1.50	0.7596	0.94	0.58	1.52	0.7940
CD40LG	rs3092945	T/C	x	0.79	3927	M+F	SMA only	0.69	227	A	1.94	1.41	2.68	0.0001	1.35	1.14	1.59	0.0005
CD40LG	rs3092945	T/C	x	0.79	3927	F	SMA only	0.69	227	H	1.78	1.30	2.43	0.0005	1.74	1.27	2.39	0.0008
CD40LG	rs3092945	T/C	x	0.79	3927	M	SMA only	0.69	227	HM	0.65	0.44	0.95	0.0321	0.66	0.45	0.97	0.0411
G6PD	rs1050828	C/T	x	0.81	3940	M+F	SMA only	0.76	229	R	1.74	1.23	2.48	0.0033	1.63	1.13	2.36	0.0122
G6PD	rs1050828	C/T	x	0.81	3940	F	SMA only	0.76	229	H	0.85	0.54	1.33	0.4602	0.84	0.54	1.32	0.4465
G6PD	rs1050828	C/T	x	0.81	3940	M	SMA only	0.76	229	HM	1.72	1.16	2.54	0.0093	1.76	1.18	2.60	0.0070
G6PD	rs1050829	T/C	x	0.60	3928	M+F	SMA only	0.57	229	R	0.80	0.57	1.11	0.1659	1.12	0.83	1.51	0.4527
G6PD	rs1050829	T/C	x	0.60	3928	F	SMA only	0.57	229	H	0.84	0.62	1.13	0.2401	0.83	0.62	1.13	0.2426
G6PD	rs1050829	T/C	x	0.60	3928	M	SMA only	0.57	229	HM	0.75	0.53	1.07	0.1164	0.76	0.53	1.08	0.1282

* The rs8176719 polymorphism identifies blood-group O in the homozygous deletion form. The derived allele of rs8176746 identifies blood-group B. Chr = chromosome; DAF = derived allele frequency; MO = genotype model [A – additive, D – dominant, H – heterozygote, HM – hemizygous males, R – recessive] with respect to the derived allele; OR = Odds Ratio; LCI = Lower Confidence Interval (95%); UCI = Upper Confidence Interval; P = p-value using a logistic regression model as described in methods. § ORs and p-values unadjusted and adjusted for ethnic group, gender and *rs334*. Only those individuals solely having SMA were included in this analysis.

Table S10: Odds ratios for SMA only for all polymorphisms investigated.

Gene	SNPID	Alleles (ancestral/ derived)	Chr	DAF Controls	No. of genotype in controls	Sample	Case Phenotype	DAF Cases	No. of genotypes in cases	MO	Unadjusted				Adjusted§			
											OR	LCL	UCL	P	OR	LCL	UCL	P
AJAP1	rs113788643	A/G	1	0.08	3940	All	RD_only	0.06	157	R	-	-	-	-	-	-	-	-
AJAP1	rs6674631	G/A	1	0.13	3928	All	RD_only	0.14	157	H	1.28	0.89	1.85	0.1872	1.33	0.92	1.93	0.15938
ATP2B4	rs10900585	G/T	1	0.34	3762	All	RD_only	0.37	157	H	1.66	1.05	2.85	0.0229	1.67	1.08	2.44	0.0251
ATP2B4	rs1541255	A/G	1	0.33	3933	All	RD_only	0.36	157	H	0.63	0.48	0.87	0.0275	0.66	0.50	0.90	0.027579
ATP2B4	rs3753036	G/A	1	0.04	3939	All	RD_only	0.04	157	R	4.94	0.57	42.62	0.2241	7.94	0.83	76.21	0.2218
ATP2B4	rs4951074	G/A	1	0.32	3934	All	RD_only	0.35	157	H	1.32	0.96	1.82	0.0879	1.09	0.59	2.04	0.063237
ATP2B4	rs55868763	G/C	1	0.33	3931	All	RD_only	0.36	157	H	1.39	1.01	1.92	0.0432	1.49	1.10	1.98	0.0440
CR1	rs17047660	A/G	1	0.16	3923	All	RD_only	0.18	156	A	1.15	0.85	1.55	0.3637	1.22	0.90	1.66	0.33548
CR1	rs17047661	G/A	1	0.32	3934	All	RD_only	0.33	155	R	1.37	0.85	2.22	0.2151	1.22	0.73	2.03	0.2094
DARC	rs2814778	G/A	1	0.00	3944	All	RD_only	0.00	151	H	-	-	-	-	-	-	-	-
GBP7	rs1803632	G/C	1	0.49	3942	All	RD_only	0.49	156	H	0.85	0.61	1.17	0.3037	0.89	0.64	1.23	0.3454
IL10	rs1800890	A/T	1	0.24	3945	All	RD_only	0.22	156	R	0.29	0.09	0.91	0.0092	0.27	0.08	0.88	0.009432
IL10	rs1800896	T/C	1	0.38	3943	All	RD_only	0.36	156	R	0.75	0.45	1.25	0.2542	0.74	0.44	1.25	0.2693
IL10	rs3024500	G/A	1	0.45	3928	All	RD_only	0.45	155	H	1.35	0.97	1.87	0.0707	1.36	0.98	1.90	0.057556
LPHN2	rs146428334	A/G	1	0.00	3930	All	RD_only	0.01	157	H	1.53	0.36	6.43	0.5876	1.54	0.36	6.61	0.5752
LPHN2	rs4650365	T/C	1	0.20	3936	All	RD_only	0.19	157	R	0.70	0.25	1.91	0.4562	0.77	0.28	2.11	0.48287
LPHN2	rs72933304	C/A	1	0.09	3942	All	RD_only	0.09	157	H	1.12	0.73	1.70	0.6173	1.14	0.74	1.76	0.5766
LPHN2	rs72933310	T/C	1	0.09	3927	All	RD_only	0.10	157	H	1.13	0.75	1.71	0.5678	1.14	0.75	1.74	0.52604
LPHN2	rs72933350	T/C	1	0.05	3940	All	RD_only	0.07	157	A	1.40	0.89	2.22	0.1663	1.47	0.92	2.35	0.1538
IL1A	rs17561	G/T	2	0.16	3938	All	RD_only	0.20	156	R	2.15	1.02	4.51	0.0664	2.39	1.13	5.09	0.064612
IL1B	rs1143634	C/T	2	0.11	3941	All	RD_only	0.10	156	A	0.84	0.57	1.22	0.3494	0.81	0.55	1.20	0.3628
LAPTM4A	rs973128	C/G	2	0.44	3885	All	RD_only	0.35	156	D	0.55	0.40	0.75	0.0003	0.53	0.38	0.74	0.000191
LOC727982	rs10188961	A/G	2	0.40	3934	All	RD_only	0.44	157	A	1.20	0.95	1.50	0.1242	1.17	0.93	1.47	0.1616
LOC727982	rs1371474	T/C	2	0.27	3933	All	RD_only	0.31	157	H	1.63	1.18	2.25	0.0029	1.69	1.22	2.35	0.002967
LOC727982	rs1371478	C/T	2	0.27	3909	All	RD_only	0.30	157	H	1.71	1.24	2.36	0.0011	1.73	1.25	2.40	0.0019
SDC1	rs11899121	C/G	2	0.47	3921	All	RD_only	0.50	157	D	1.24	0.85	1.80	0.2514	1.27	0.87	1.85	0.29507
ZSWIM2	rs144778284	C/T	2	0.00	3937	All	RD_only	0.00	157	H	1.13	0.15	8.51	0.9058	1.31	0.17	10.14	0.9004
ZSWIM2	rs4316902	A/G	2	0.18	3910	All	RD_only	0.17	153	H	0.75	0.51	1.10	0.1343	0.79	0.53	1.16	0.16013
ARL14	rs74954675	A/C	3	0.08	3907	All	RD_only	0.08	156	R	-	-	-	-	-	-	-	-
ARL14	rs75731597	A/C	3	0.08	3491	All	RD_only	0.10	157	R	-	-	-	-	-	-	-	-
ARL14	rs76033371	A/G	3	0.09	3939	All	RD_only	0.10	157	R	-	-	-	-	-	-	-	-
B3GALNT1	rs12107243	G/C	3	0.49	3934	All	RD_only	0.46	157	A	0.89	0.71	1.13	0.3422	0.93	0.74	1.18	0.39756
IL17RD	rs6780995	A/G	3	0.41	3939	All	RD_only	0.40	156	R	0.96	0.62	1.47	0.8356	0.98	0.63	1.51	0.8753
IL17RE	rs708567	A/G	3	0.50	3881	All	RD_only	0.45	154	A	0.82	0.66	1.04	0.0967	0.81	0.64	1.02	0.11682
TLR9	rs187084	T/C	3	0.33	3933	All	RD_only	0.33	151	R	0.83	0.48	1.43	0.4990	1.14	0.81	1.58	0.4593
FREM3	rs149914432	A/C	4	0.10	3915	All	RD_only	0.06	157	H	0.46	0.26	0.80	0.0025	0.48	0.24	0.95	0.002561
FREM3	rs184895969	C/T	4	0.00	3940	All	RD_only	0.01	157	H	3.13	1.09	9.02	0.0649	8.15	2.19	30.36	0.0633
FREM3	rs184908374	G/A	4	0.00	3943	All	RD_only	0.01	157	H	2.90	0.86	9.74	0.1313	6.51	1.54	27.44	0.12848
FREM3	rs186790584	A/T	4	0.10	3941	All	RD_only	0.06	157	H	0.47	0.27	0.81	0.0031	0.47	0.27	0.8	0.0033
FREM3	rs186873296	A/G	4	0.10	3926	All	RD_only	0.06	157	H	0.47	0.27	0.83	0.0038	0.48	0.29	0.82	0.003987
GAB1	rs7663712	A/G	4	0.02	3938	All	RD_only	0.01	157	A	0.51	0.19	1.39	0.1433	0.51	0.19	1.38	0.1530
INPP4B	rs13103597	C/T	4	0.27	3925	All	RD_only	0.21	157	D	0.64	0.46	0.90	0.0086	0.70	0.48	1.02	0.004952
INPP4B	rs77389579	G/T	4	0.07	3937	All	RD_only	0.04	157	A	0.57	0.32	1.01	0.0358	0.70	0.46	0.9	0.0213
TLR1	rs4833095	C/T	4	0.09	3921	All	RD_only	0.12	150	A	1.40	0.98	2.01	0.0764	1.31	0.90	1.89	0.070727
TLR6	rs5743809	T/C	4	0.06	3884	All	RD_only	0.06	145	R	3.68	0.82	16.40	0.1441	3.71	0.81	17.04	0.1424
TLR6	rs5743810	C/T	4	0.02	3936	All	RD_only	0.03	151	H	2.02	1.00	4.09	0.0737	2.02	0.99	4.14	0.071669
USP38	rs28459062	T/C	4	0.16	3931	All	RD_only	0.18	157	D	1.11	0.79	1.57	0.5522	1.05	0.78	1.43	0.5949
USP38	rs4266246	C/T	4	0.22	3928	All	RD_only	0.22	157	R	1.42	0.73	2.74	0.3195	1.34	0.69	2.61	0.29847
C6	rs1801033	A/C	5	0.47	3927	All	RD_only	0.44	150	D	0.72	0.51	1.02	0.0654	0.72	0.51	1.02	0.0586
IL13	rs20541	C/T	5	0.22	3796	All	RD_only	0.24	144	A	1.13	0.86	1.48	0.3936	1.13	0.86	1.48	0.34347
IRF	rs2706384	C/A	5	0.38	3819	All	RD_only	0.38	144	R	1.13	0.72	1.79	0.6008	1.06	0.67	1.69	0.5697
CTL4	rs2242665	A/G	6	0.26	3853	All	RD_only	0.32	150	R	1.83	1.10	3.05	0.0289	1.32	1.02	1.70	0.025206
GABBR1	rs192151845	G/A	6	0.01	3943	All	RD_only	0.01	157	H	1.33	0.41	4.32	0.6438	0.88	0.21	3.67	0.8750
HCG4	rs114980857	C/T	6	0.21	3843	All	RD_only	0.20	150	A	0.91	0.69	1.22	0.5392	0.89	0.67	1.19	0.57831
HSPA1B	rs6457452	C/T	6	0.19	3917	All	RD_only	0.17	157	A	0.86	0.63	1.16	0.3047	0.85	0.63	1.16	0.2810
IL20RA	rs1555498	C/T	6	0.46	3947	All	RD_only	0.44	156	D	0.82	0.58	1.15	0.2508	0.87	0.61	1.24	0.30236
LTA	rs2239704	G/T	6	0.12	3896	All	RD_only	0.14	153	H	1.37	0.95	1.97	0.0955	1.32	0.91	1.91	0.0873
LTA	rs909253	T/C	6	0.49	3921	All	RD_only	0.48	155	D	0.96	0.67	1.38	0.8402	1.00	0.79	1.25	0.91093
PLEKHG1	rs114726617	G/A	6	0.04	3940	All	RD_only	0.05	157	H	1.41	0.81	2.43	0.2391	1.37	0.77	2.44	0.3355
PLEKHG1	rs141555199	C/G	6	0.02	3943	All	RD_only	0.02	157	R	-	-	-	-	-	-	-	-
PLEKHG1	rs142712208	G/A	6	0.03	3927	All	RD_only	0.04	157	H	1.29	0.70	2.36	0.4273	1.38	0.74	2.57	0.3974
PLEKHG1	rs15116938	C/T	6	0.03	3939	All	RD_only	0.04	157	R	-	-	-	-	-	-	-	-
PLEKHG1	rs151293197	C/G	6	0.03	3940	All	RD_only	0.04	156	H	1.25	0.68	2.29	0.4826	1.32	0.71	2.46	0.4501
PLEKHG1	rs2131263	C/T	6	0.04	3938	All	RD_only	0.05	157	H	1.41	0.81	2.43	0.2397	1.37	0.77	2.44	0.33629
PLEKHG1	rs55958968	A/G	6	0.05	3932	All	RD_only	0.07	157	H	1.42	0.89	2.29	0.1578	1.39	0.85	2.25	0.1400
PLEKHG1	rs76924464	G/A	6	0.03	3940	All	RD_only	0.04	157	R	-	-	-	-	-	-	-	-
PLEKHG1	rs79100774	G/C	6	0.03	3939	All	RD_only	0.04	157	R	-	-	-	-	-	-	-	-
SNORD48	rs116288147	C/T	6	0.24	3896	All	RD_only	0.21	156	A	0.86	0.65	1.14	0.2896	0.55	0.22	1.39	0.17612
TNF	rs1799964	T/C	6	0.27	3938	All	RD_only	0.24	155	R	0.58	0.27	1.25	0.1315	0.50	0.22	1.14	0.0640
TNF	rs1800629	G/A	6	0.09	3940	All	RD_only	0.09	156	R	3.46	1.20	9.97	0.0477	3.37	1.13	9.98	0.04709
TNF	rs1800750	G/A	6	0.05	3868	All	RD_only	0.03	58	A	0.66	0.24	1.77	0.3711	0.67	0.25	1.81	0.3678
TNF	rs3093662	A/G	6	0.11	3936	All	RD_only	0.10	154	D	0.87	0.58	1.32	0.5198	0.87	0.57	1.32	0.54979
TNF	rs361525	G/A	6	0.07	3948	All	RD_only	0.05	156	R	-	-	-	-	-	-	-	-
CD36	rs3211938	T/G	7	0.10	3942	All	RD_only	0.09	155	H	0.88	0.56	1.37	0.5574	0.89	0.57	1.40	0.57785
NOD1	rs2075820	G/A	7	0.38	3941	All	RD_only	0.37	151	R	0.94	0.59	1.51	0.8016	1.08	0.78	1.51	0.7891
ABO	rs56390333	G/A	9	0.00	3942	All	RD_only	0.00	156	H	-	-	-	-	-	-	-	-
ABO	rs8176719*	O vs non-O	9	0.26	3888	All	RD_only	0.33	156	R	0.52	0.31	0.81	0.0074	0.53	0.34	0.83	0.0089
ABO	rs8176746*	C/A	9	0.13	3924	All	RD_only	0.18	156	A	1.59	1.13	2.24	0.0092	1.34	0.89	2.02	0.012755
ABO	rs8176750	I/D	9	0.05	3923	All	RD_only	0.04	155	R	-	-	-	-	-	-	-	-
TLR4	rs4986790	A/G	9	0.04	3929	All	RD_only	0.03	151	R	-	-	-	-	-	-	-	-
TLR4	rs4986791	C/T	9	0.01	3937	All	RD_only	0.01	150	H	2.16	0.77	6.08	0.1861	2.43	0.85	6.94	0.1839

MKI67	rs11016116	A/G	10	0.02	3944	All	RD_only	0.03	157	R	-	-	-	-	-	-	-	-
MKI67	rs115947774	A/G	10	0.00	3931	All	RD_only	0.00	156	H	0.91	0.12	6.79	0.9269	0.79	0.10	5.98	0.9345
MKI67	rs148494166	T/C	10	0.00	3913	All	RD_only	0.01	146	H	3.58	0.81	15.78	0.1496	3.30	0.74	14.82	0.14532
HBB	rs334	A/T	11	0.08	3949	All	RD_only	0.02	156	A	0.18	0.08	0.45	$5.0x10^{-07}$	0.19	0.08	0.46	$5.8x10^{-07}$
RRM1	rs116472045	A/G	11	0.02	3939	All	RD_only	0.04	157	H	2.64	1.42	4.90	0.0060	2.59	1.38	4.86	0.005081
RTN3	rs542998	T/C	11	0.43	3929	All	RD_only	0.41	147	H	0.88	0.63	1.22	0.4422	0.82	0.58	1.15	0.3977
TRIM5	rs7935564	G/A	11	0.44	3871	All	RD_only	0.47	151	R	1.40	0.95	2.05	0.0963	1.29	0.87	1.90	0.091014
CAND1	rs10459266	C/A	12	0.21	3938	All	RD_only	0.28	157	D	1.69	1.23	2.33	0.0014	1.75	1.26	2.42	0.0009
CAND1	rs12307123	C/T	12	0.23	3933	All	RD_only	0.26	156	A	1.22	0.94	1.58	0.1323	1.17	0.90	1.52	0.17144
CAND1	rs1566830	G/T	12	0.26	3940	All	RD_only	0.30	157	R	1.76	1.05	2.97	0.0449	1.76	1.04	2.98	0.0409
GRIP1	rs1394263	T/C	12	0.26	3914	All	RD_only	0.30	157	R	1.76	1.05	2.96	0.0459	1.74	1.03	2.95	0.041826
IL22	rs1012356	A/T	12	0.49	3940	All	RD_only	0.47	156	R	0.85	0.57	1.26	0.4147	0.88	0.59	1.31	0.4368
IL22	rs2227478	A/G	12	0.38	3939	All	RD_only	0.40	156	H	1.19	0.86	1.64	0.2908	1.25	0.90	1.73	0.24654
IL22	rs2227485	G/A	12	0.42	3928	All	RD_only	0.40	156	R	0.68	0.42	1.09	0.0939	0.67	0.41	1.08	0.0977
IL22	rs2227491	C/T	12	0.40	3870	All	RD_only	0.41	151	H	1.14	0.82	1.58	0.4365	1.13	0.81	1.57	0.38947
IL22	rs2227507	T/C	12	0.04	3948	All	RD_only	0.04	156	R	-	-	-	-	-	-	-	-
TPTE2	rs182873742	C/G	13	0.00	3940	All	RD_only	0.00	157	H	-	-	-	-	-	-	-	-
RPS6KL1	rs3742785	C/A	14	0.29	3714	All	RD_only	0.25	148	R	0.69	0.36	1.33	0.2424	0.83	0.63	1.08	0.2026
SPTB	rs229587	T/C	14	0.34	3872	All	RD_only	0.30	155	A	0.82	0.64	1.05	0.1067	0.82	0.64	1.05	0.10252
YLPM1	rs10139016	C/T	14	0.25	3937	All	RD_only	0.24	157	R	0.78	0.38	1.60	0.4752	0.78	0.37	1.61	0.5046
ADCY9	rs10775349	C/G	16	0.20	3941	All	RD_only	0.21	151	R	1.75	0.90	3.40	0.1217	1.90	0.97	3.71	0.11955
ADCY9	rs2230739	A/G	16	0.09	3944	All	RD_only	0.10	151	R	2.58	0.78	8.59	0.1706	3.13	0.92	10.67	0.1687
HBA	$\alpha^{-3.7}$-thalassaemia	I/D	16	0.41	3946	All	RD_only	0.37	153	D	0.72	0.52	0.98	0.0340	0.76	0.54	0.98	0.036851
IL4	rs2243250	T/C	16	0.20	3902	All	RD_only	0.18	154	R	0.45	0.14	1.42	0.1209	0.47	0.15	1.51	0.1230
IL4R	rs1805015	T/C	16	0.42	3927	All	RD_only	0.44	156	D	1.10	0.78	1.55	0.5977	1.12	0.81	1.56	0.63147
ADORA2B	rs2535611	T/C	17	0.10	3911	All	RD_only	0.13	151	D	1.26	0.86	1.86	0.2420	1.28	0.86	1.89	0.2169
NOS2	rs1800482	G/C	17	0.08	3922	All	RD_only	0.09	154	R	1.96	0.46	8.34	0.4061	2.50	0.56	11.15	0.40375
NOS2	rs2297518	G/A	17	0.14	3910	All	RD_only	0.14	149	R	2.08	0.89	4.87	0.1234	2.02	0.85	4.79	0.1207
NOS2	rs8078340	C/T	17	0.21	3948	All	RD_only	0.20	156	R	0.15	0.02	1.04	0.0061	0.13	0.02	0.97	0.006273
NOS2	rs9282799	C/T	17	0.03	3949	All	RD_only	0.02	155	A	0.63	0.25	1.54	0.2725	0.66	0.27	1.62	0.2840
TBX2	rs73991577	A/G	17	0.00	3937	All	RD_only	0.00	157	H	-	-	-	-	-	-	-	-
EMR1	rs373533	G/T	19	0.46	3863	All	RD_only	0.47	151	R	0.99	0.67	1.47	0.9646	1.04	0.72	1.51	0.9102
EMR1	rs461645	C/T	19	0.47	3933	All	RD_only	0.47	151	D	1.02	0.71	1.47	0.9061	1.07	0.73	1.55	0.80482
ICAM	rs5498	A/G	19	0.11	3908	All	RD_only	0.10	154	R	0.44	0.06	3.19	0.3493	0.44	0.06	3.22	0.3534
GNAS	rs8386	C/T	20	0.19	3944	All	RD_only	0.18	155	H	0.89	0.63	1.27	0.5190	0.90	0.63	1.29	0.52994
DERL3	rs1128127	G/A	22	0.50	3911	All	RD_only	0.47	156	D	0.87	0.61	1.23	0.4269	0.88	0.61	1.26	0.3972
CD40LG	rs1126535	T/C	X	0.15	3934	M+F	RD_only	0.10	156	D	0.60	0.38	0.94	0.0184	0.59	0.37	0.94	0.020226
CD40LG	rs1126535	T/C	X	0.16	1947	F	RD_only	0.10	80	A	0.59	0.35	0.99	0.0311	0.61	0.37	1.00	0.0351
CD40LG	rs1126535	C/T	X	0.85	1987	M	RD_only	0.89	76	HM	1.49	0.71	3.13	0.2740	1.50	0.73	3.17	0.2940
CD40LG	rs3092945	T/C	X	0.21	3927	M+F	RD_only	0.24	154	D	1.19	0.84	1.68	0.3412	1.15	0.81	1.64	0.3059
CD40LG	rs3092945	T/C	X	0.21	1942	F	RD_only	0.25	79	A	1.30	0.89	1.88	0.1786	1.32	0.90	1.89	0.1986
CD40LG	rs3092945	C/T	X	0.78	1985	M	RD_only	0.77	75	HM	0.99	0.57	1.71	0.9629	1.00	0.58	1.72	0.9786
G6PD	rs1050828	C/T	X	0.19	3940	M+F	RD_only	0.20	155	H	1.12	0.70	1.79	0.6468	1.05	0.64	1.70	0.64676
G6PD	rs1050828	C/T	X	0.20	1951	F	RD_only	0.22	79	A	1.12	0.75	1.67	0.5802	1.16	0.78	1.69	0.6102
G6PD	rs1050828	C/T	X	0.19	1989	M	RD_only	0.18	76	HM	0.98	0.54	1.77	0.9503	0.99	0.57	1.84	0.9808
G6PD	rs1050829	T/C	X	0.40	3928	M+F	RD_only	0.38	155	A	0.92	0.76	1.12	0.3931	0.79	0.51	1.21	0.4246
G6PD	rs1050829	T/C	X	0.41	1952	F	RD_only	0.41	79	R	0.96	0.52	1.77	0.9017	0.97	0.55	1.79	0.9617
G6PD	rs1050829	C/T	X	0.60	1976	M	RD_only	0.66	76	HM	1.27	0.78	2.05	0.3344	1.29	0.79	2.08	0.3784

* The rs8176719 polymorphism identifies blood-group O in the homozygous deletion form. The derived allele of rs8176746 identifies blood-group B. Chr = chromosome; DAF = derived allele frequency; MO = genotype model [A – additive, D – dominant, H – heterozygote, HM – hemizygous males, R – recessive] with respect to the derived allele; OR = Odds Ratio; LCI = Lower Confidence Interval (95%); UCI = Upper Confidence Interval; P = p-value using a logistic regression model as described in methods. § ORs and p-values unadjusted and adjusted for ethnic group, gender and *rs334*. Only those individuals solely having RD were included in this analysis.

Table S11: Odds ratios for RD only for all polymorphisms investigated.

		No. of cases by genotype						No. of controls by genotype						
Gene 1	Gene 2	REF REF	REF HET	HET REF	HET HET	DER REF	DER HET	REF REF	REF HET	HET REF	HET HET	DER REF	DER HET	P
HBA	*HBB*	820	16	994	22	250	13	1131	216	1649	288	537	92	0.02
HBA	*ABO*	412	369	462	457	129	110	725	501	1058	753	342	250	0.05
HBA	*LPHN2*	720	124	890	116	216	45	1147	195	1615	322	533	101	0.05
ARL14	*HBB*	1680	48	392	7	17	1	2471	464	424	70	31	3	0.06
INPP4B	*RPS6KL1*	938	826	89	64	0	1	1673	1229	250	184	8	3	0.07
HBA	*ARL14*	656	163	814	169	208	49	1026	166	1460	243	469	91	0.07
HBA	*INPP4B*	772	69	929	83	230	30	1188	1219	301	237	21	10	0.07
HBB	*LOC727982*	1844	278	44	13	7	4	2774	520	495	94	28	5	0.08
ABO	*IL10*	694	404	590	347	124	70	1239	728	839	582	156	87	0.08
HBB	*LPHN2*	1844	278	44	13	7	4	2774	520	495	94	28	5	0.09
LOC727982	*RPS6KL1*	524	426	435	396	71	70	1029	749	726	538	168	119	0.11
ATP2B4	*FREM3*	895	105	900	109	165	19	1425	288	1434	312	342	69	0.13
LOC727982	*LPHN2*	946	136	806	145	148	15	1764	330	1231	225	268	51	0.13
INPP4B	*LOC727982*	980	886	102	67	0	2	1835	1260	275	195	6	7	0.15
ATP2B4	*RPS6KL1*	451	430	474	409	102	54	849	628	871	640	208	147	0.20
ABO	*INPP4B*	928	102	901	69	183	13	1825	286	1308	190	221	32	0.20
ARL14	*IL10*	1014	648	239	145	14	4	1697	1061	295	171	16	16	0.23
ABO	*ATP2B4*	474	460	448	453	82	100	726	967	665	672	118	112	0.29
IL10	*LPHN2*	1128	163	690	117	82	16	1905	341	1185	224	203	54	0.30
HBA	*FREM3*	750	86	892	116	235	23	1129	204	1575	355	510	110	0.35
IL10	*INPP4B*	1179	113	752	57	83	15	1954	302	1234	174	215	40	0.36
ARL14	*INPP4B*	1601	143	362	38	14	4	2573	370	420	77	30	4	0.40
IL10	*RPS6KL1*	598	533	382	317	48	41	1108	829	690	508	135	83	0.42
ATP2B4	*HBB*	971	26	983	25	175	6	1487	244	1466	287	354	62	0.44
FREM3	*INPP4B*	1910	48	102	131	2	2	3122	83	255	408	12	20	0.45
ARL14	*LOC727982*	855	763	203	166	8	9	1604	1083	261	197	16	15	0.50
ATP2B4	*INPP4B*	911	95	932	81	172	12	1510	219	1527	242	360	55	0.50
HBA	*IL10*	489	318	613	368	156	92	791	473	1112	712	366	232	0.50
ARL14	*FREM3*	1554	183	357	42	13	4	2432	488	392	102	18	4	0.50
ABO	*HBB*	1006	26	957	25	192	5	1779	328	1282	210	205	51	0.51
ARL14	*ATP2B4*	802	796	182	190	6	10	1329	1318	202	244	14	17	0.54
IL10	*LOC727982*	640	565	400	346	45	40	1214	851	777	514	128	101	0.54
INPP4B	*LPHN2*	1736	271	164	24	1	1	2859	522	421	93	40	4	0.56
FREM3	*HBB*	1899	48	220	8	5	0	2716	478	552	109	32	7	0.58
FREM3	*LOC727982*	965	853	115	99	3	3	1744	1181	357	262	19	16	0.65
HBB	*IL10*	1270	791	31	24	8	3	1902	1196	349	211	19	11	0.66
FREM3	*RPS6KL1*	914	806	112	82	1	4	1570	1177	331	233	26	8	0.68
HBA	*RPS6KL1*	391	338	479	398	121	112	639	498	985	683	311	234	0.69
ARL14	*RPS6KL1*	819	705	185	161	6	10	1429	1070	252	176	18	13	0.74
ARL14	*LPHN2*	1511	235	347	51	16	2	2467	471	428	67	26	8	0.74
HBB	*INPP4B*	1944	181	53	4	10	1	2880	421	503	87	24	13	0.75
ABO	*RPS6KL1*	488	415	443	399	95	77	1042	751	737	552	126	95	0.75
FREM3	*IL10*	1146	731	140	78	3	2	1840	1169	392	280	24	13	0.76
ATP2B4	*LOC727982*	488	438	502	440	91	79	929	634	967	645	221	170	0.76
LPHN2	*RPS6KL1*	900	761	126	130	3	3	1625	1175	294	236	12	9	0.86
ABO	*LPHN2*	886	147	842	123	169	26	1756	354	1276	218	217	34	0.86
ABO	*FREM3*	913	112	873	96	174	21	1735	355	1228	257	201	49	0.88
HBA	*ATP2B4*	375	392	476	455	116	120	588	605	876	874	276	294	0.91
FREM3	*LPHN2*	1692	263	302	30	5	1	2694	494	552	113	33	7	0.91
HBA	*LOC727982*	414	362	497	443	127	116	740	479	1046	744	336	242	0.95
ATP2B4	*IL10*	588	370	595	376	111	65	1005	620	1017	640	238	153	0.97
ABO	*LOC727982*	498	451	486	415	100	84	1140	792	815	554	141	92	0.98
HBB	*RPS6KL1*	998	858	23	25	5	5	1639	1173	280	238	16	11	0.99

REF: reference homozygote genotype; HET: Heterozygote genotype; DER: derived allele homozygote genotype. p-values were adjusted for ethnicity and gender.

Table S12: Pairwise interactions between the polymorphisms (1 per gene) at autosomal loci at which we found significant associations in our primary analyses using the likelihood ratio approach (Main Text Table 2).

Gene	SNP ID	genotype	Hb						MCV						RBC					
			N	G mean	LCL	UCL	P value	Regress P*	N	G mean	LCL	UCL	P value	Regress P*	N	G mean	LCL	UCL	P value	Regress P*
ATP2B4	rs1541255	AA	1010	60.58	59.03	62.17	NA		963	73.67	73.09	74.25	NA		966	2.64	2.57	2.72	NA	
ATP2B4	rs1541255	AG	1016	62.22	60.63	63.84	0.33	0.13	969	73.59	73.01	74.18	0.98	0.77	969	2.70	2.63	2.78	0.49	0.20
ATP2B4	rs1541255	GG	185	63.72	59.97	67.70	0.29		176	73.55	72.20	74.92	0.99		177	2.76	2.59	2.95	0.44	
IL10	rs1800890	AA	1313	61.33	59.95	62.74	NA		1264	73.81	73.30	74.33	NA		1264	2.67	2.60	2.73	NA	
IL10	rs1800890	AT	821	61.48	59.74	63.28	0.99	0.40	774	73.11	72.47	73.77	0.22	0.10	778	2.68	2.59	2.76	0.99	0.43
IL10	rs1800890	TT	99	58.69	54.02	63.76	0.57		92	73.69	71.81	75.61	0.99		92	2.63	2.40	2.88	0.94	
LPHN2	rs72933304	CC	1912	61.77	60.62	62.95	NA		1828	73.64	73.22	74.06	NA		1829	2.68	2.63	2.74	NA	
LPHN2	rs72933304	AC	296	60.74	57.90	63.72	0.80	0.65	278	73.50	72.43	74.60	0.97	0.09	281	2.67	2.54	2.82	0.99	0.43
LPHN2	rs72933304	AA	8	63.64	47.50	85.10	0.98		7	79.81	72.70	87.50	0.21		7	2.65	1.90	3.69	1.00	
LOC727982	rs1371478	CC	1088	62.43	60.89	64.01	NA		1035	74.03	73.47	74.59	NA		1037	2.70	2.63	2.78	NA	
LOC727982	rs1371478	CT	958	61.23	59.61	62.88	0.55	0.03	915	73.29	72.70	73.89	0.18	0.05	918	2.67	2.59	2.75	0.79	0.15
LOC727982	rs1371478	TT	163	58.61	54.94	62.52	0.17		156	72.93	71.52	74.38	0.35		156	2.60	2.42	2.79	0.56	
ARL14	rs75731597	AA	1754	62.00	60.79	63.23	NA		1674	73.68	73.24	74.12	NA		1676	2.69	2.63	2.75	NA	
ARL14	rs75731597	AC	401	61.21	58.74	63.78	0.85	0.19	384	73.15	72.24	74.07	0.57	0.006	385	2.70	2.59	2.83	0.98	0.02
ARL14	rs75731597	CC	18	56.54	46.57	68.66	0.62		16	80.60	75.81	85.69	0.01		17	2.14	1.73	2.64	0.09	
FREM3	rs186873296	AA	1967	61.36	60.23	62.51	NA		1875	73.66	73.24	74.08	NA		1876	2.67	2.62	2.73	NA	
FREM3	rs186873296	AG	233	62.94	59.63	66.44	0.66	0.01	222	73.54	72.34	74.76	0.98	0.51	225	2.72	2.56	2.88	0.84	0.10
FREM3	rs186873296	GG	6	84.39	60.20	118.10	0.15		6	71.46	64.60	78.90	0.82		6	3.08	2.15	4.41	0.72	
INPP4B	rs77389579	GG	2019	61.40	60.28	62.53	NA		1927	73.68	73.26	74.09	NA		1928	2.67	2.62	2.73	NA	
INPP4B	rs77389579	GT	188	63.77	60.05	67.73	0.46	0.16	178	73.25	71.91	74.61	0.83	0.07	180	2.74	2.57	2.93	0.73	0.18
INPP4B	rs77389579	TT	2	56.58	31.50	101.00	0.96		2	62.09	52.10	73.80	0.13		2	2.90	1.55	5.41	0.96	
ABO	rs8176719	Non-O	1178	57.49	55.95	59.07	NA	0.35	1125	71.56	70.97	72.15	NA	0.08	1126	2.54	2.29	2.44	NA	0.45
ABO	rs8176719	O	1045	62.87	61.29	64.49	0.80		996	73.78	73.20	74.36	0.13		999	2.76	2.68	2.84	0.96	
ABO	rs8176746	CC	1529	6.19	6.06	6.32	NA		189	72.36	71.07	73.67	NA		1458	2.70	2.64	2.76	NA	
ABO	rs8176746	AC	622	6.02	5.83	6.23	0.35	0.08	942	73.56	72.97	74.15	0.23	0.23	598	2.61	2.52	2.70	0.26	0.07
ABO	rs8176746	AA	76	5.79	5.27	6.37	0.37		996	73.78	73.20	74.36	0.13		73	2.59	2.34	2.88	0.74	
HBB	rs334	AA	2159	61.27	60.20	62.35	NA		2058	73.55	73.15	73.94	NA		2062	2.67	2.62	2.72	NA	
HBB	rs334	AT	57	73.54	66.01	81.92	0.003	5.1×10^{-7}	55	70.89	68.59	73.28	0.08	1.0×10^{-9}	55	3.40	3.03	3.82	0.0002	8.57×10^{-11}
HBB	rs334	TT	11	30.07	23.52	38.45	5.1×10^{-8}		11	89.56	83.18	96.44	6.1×10^{-7}		11	1.13	0.87	1.46	4.3×10^{-10}	
RPS6KL1	rs3742785	CC	1032	62.86	61.26	64.50	NA		985	73.54	72.97	74.12	NA		987	2.71	2.64	2.79	NA	
RPS6KL1	rs3742785	CA	896	59.98	58.35	61.67	0.04	0.001	855	73.73	73.12	74.36	0.90	0.24	857	2.63	2.55	2.71	0.28	0.01
RPS6KL1	rs3742785	AA	187	57.98	54.57	61.60	0.04		176	74.34	72.97	75.73	0.55		176	2.50	2.34	2.67	0.06	
$\alpha^{-3.7}$thalassemia		Norm	853	61.12	59.43	62.87	NA		819	76.78	76.16	77.41	NA		819	2.54	2.46	2.61	NA	
$\alpha^{-3.7}$thalassemia		Het	1026	62.23	60.66	63.85	0.62		974	72.88	72.34	73.43	5.4×10^{-11}	8.3×10^{-61}	975	2.72	2.65	2.80	0.002	
$\alpha^{-3.7}$thalassemia		Hom	264	62.16	59.09	65.38	0.84	0.49	252	66.80	65.82	67.78	5.4×10^{-11}		254	3.03	2.87	3.20	6.6×10^{-8}	1.11×10^{-10}
CD40LG§	rs3092945	TT	1459	61.93	60.61	63.28	NA		1390	73.48	73.00	73.97	NA		1392	2.70	2.64	2.76	NA	
CD40LG	rs3092945	TC	388	60.00	57.55	62.56	0.38	0.12	366	74.66	73.70	75.63	0.08	0.28	369	2.56	2.45	2.68	0.12	0.28
CD40LG§	rs3092945	CC	379	60.52	58.02	63.13	0.61		367	72.75	71.82	73.70	0.37		366	2.68	2.56	2.81	0.97	
G6PD§	rs1050828	CC	1643	61.93	60.68	63.20	NA		1574	73.17	72.71	73.62	NA		1575	2.71	2.65	2.77	NA	
G6PD	rs1050828	CT	306	61.76	58.92	64.74	0.99	0.001	286	75.12	74.03	76.23	0.003	0.003	288	2.58	2.45	2.72	0.20	0.01
G6PD§	rs1050828	TT	271	57.04	54.26	59.97	0.008		257	74.33	73.19	75.48	0.15		258	2.53	2.39	2.67	0.06	

Only those polymorphisms with the most significant associations with severe malaria phenotypes (main text Table 2) were analysed with red blood cell indices.
N = number of individuals; G mean = geometric mean; LCL = lower confidence level (95%), UCL = upper confidence level (95%); HB = haemoglobin (g/l); MCV = mean cell volume (fL); RBC = red blood cell count ($x10^{12}$/l); P value; Regress P = adjusted for gender, age, rs334 and ethnicity using linear regression. §Hemizygous males are coded as homozygotes.

Table S13: Association of Red Blood Cell indices for all severe malaria cases.

Gene	SNPID	*Individual loci							§Combined - 13 loci		
		Observed scale		Logit scale		Probit scale			Observed scale	Logit scale	Probit scale
		Case-control population	General population	Case-control population	General population	Case-control population	General population		General population	General population	General population
HBB	rs334	0.0390	0.0282	0.0361	0.0334	0.0361	0.0033		0.0295	0.0354	0.0354
FREM3	rs186873296	0.0098	0.0071	0.0080	0.0078	0.0080	0.0334		0.0064	0.0076	0.0076
FREM3	rs186790584	0.0092	0.0066	0.0074	0.0073	0.0074	0.0078				
FREM3	rs149914432	0.0089	0.0064	0.0072	0.0071	0.0072	0.0073				
ABO	rs8176746	0.0065	0.0047	0.0049	0.0048	0.0049	0.0071		0.0041	0.0044	0.0045
ABO	rs8176719	0.0061	0.0044	0.0046	0.0046	0.0046	0.0048		0.0011	0.0012	0.0011
INPP4B	rs77389579	0.0055	0.0040	0.0044	0.0044	0.0044	0.0046		0.0001	0.0003	0.0003
HBA	$\alpha^{-3.7}$-thalassaemia	0.0042	0.0031	0.0033	0.0033	0.0033	0.0044		0.0021	0.0023	0.0022
INPP4B	rs13103597	0.0034	0.0025	0.0026	0.0026	0.0026	0.0026				
LOC727982	rs1371478	0.0033	0.0024	0.0025	0.0025	0.0025	0.0025		0.0027	0.0030	0.0029
LOC727982	rs1371474	0.0031	0.0023	0.0024	0.0024	0.0024	0.0024				
ARL14	rs75731597	0.0031	0.0022	0.0023	0.0023	0.0023	0.0023		0.0019	0.0021	0.0020
ARL14	rs74954675	0.0026	0.0019	0.0020	0.0019	0.0020	0.0019				
ARL14	rs76033371	0.0025	0.0018	0.0019	0.0018	0.0019	0.0018				
IL10	rs1800890	0.0018	0.0013	0.0015	0.0015	0.0015	0.0015		0.0013	0.0015	0.0015
IL10	rs3024500	0.0018	0.0013	0.0013	0.0013	0.0013	0.0013				
RPS6KL1	rs3742785	0.0016	0.0012	0.0012	0.0012	0.0012	0.0012		0.0008	0.0009	0.0009
EMR1	rs373533	0.0015	0.0011	0.0012	0.0012	0.0012	0.0012		0.0012	0.0013	0.0014
EMR1	rs461645	0.0014	0.0010	0.0011	0.0011	0.0011	0.0011				
LPHN2	rs72933304	0.0014	0.0010	0.0011	0.0011	0.0011	0.0011		0.0007	0.0008	0.0008
ATP2B4	rs55868763	0.0013	0.0010	0.0010	0.0010	0.0010	0.0010		0.0007	0.0007	0.0008
ATP2B4	rs1541255	0.0013	0.0010	0.0010	0.0010	0.0010	0.0010	**Total**	**0.0525**	**0.0571**	**0.0569**
USP38	rs28459062	0.0013	0.0010	0.0010	0.0010	0.0010	0.0010				
IL1A	rs17561	0.0013	0.0010	0.0010	0.0010	0.0010	0.0010				
LPHN2	rs4650365	0.0012	0.0009	0.0009	0.0009	0.0009	0.0009				
ATP2B4	rs10900585	0.0012	0.0009	0.0009	0.0009	0.0009	0.0009				
DERL3	rs1128127	0.0012	0.0009	0.0009	0.0009	0.0009	0.0009				
ATP2B4	rs4951074	0.0011	0.0008	0.0009	0.0009	0.0009	0.0009				
TRIM5	rs7935564	0.0011	0.0008	0.0008	0.0008	0.0008	0.0008				
LOC727982	rs10188961	0.0010	0.0007	0.0008	0.0008	0.0008	0.0008				
IL20RA	rs1555498	0.0010	0.0007	0.0008	0.0008	0.0008	0.0008				
GBP7	rs1803632	0.0010	0.0007	0.0008	0.0008	0.0008	0.0008				
IL22	rs2227485	0.0010	0.0007	0.0008	0.0008	0.0008	0.0008				
TLR4	rs4986790	0.0007	0.0005	0.0007	0.0007	0.0007	0.0007				
IRF1	rs2706384	0.0009	0.0007	0.0007	0.0007	0.0007	0.0007				
GRIP1	rs1394263	0.0010	0.0007	0.0007	0.0007	0.0007	0.0007				
LPHN2	rs72933350	0.0009	0.0007	0.0007	0.0007	0.0007	0.0007				
IL4	rs2243250	0.0009	0.0007	0.0007	0.0007	0.0007	0.0007				
HSPA1B	rs6457452	0.0009	0.0006	0.0007	0.0007	0.0007	0.0007				
ADCY9	rs2230739	0.0008	0.0006	0.0006	0.0006	0.0006	0.0006				
LPHN2	rs72933310	0.0008	0.0006	0.0006	0.0006	0.0006	0.0006				
CAND1	rs1566830	0.0008	0.0006	0.0006	0.0006	0.0006	0.0006				
IL22	rs2227491	0.0008	0.0006	0.0006	0.0006	0.0006	0.0006				
IL22	rs1012356	0.0008	0.0006	0.0006	0.0006	0.0006	0.0006				
PLEKHG1	rs141555199	0.0007	0.0005	0.0006	0.0006	0.0006	0.0006				
ADORA2B	rs2535611	0.0007	0.0005	0.0005	0.0005	0.0005	0.0005				
GNAS	rs8386	0.0007	0.0005	0.0005	0.0005	0.0005	0.0005				
PLEKHG1	rs55958968	0.0007	0.0005	0.0005	0.0005	0.0005	0.0005				
CAND1	rs10459266	0.0007	0.0005	0.0005	0.0005	0.0005	0.0005				
IL1B	rs1143634	0.0006	0.0004	0.0005	0.0005	0.0005	0.0005				
TBX2	rs73991577	0.0005	0.0004	0.0005	0.0005	0.0005	0.0005				
TLR1	rs4833095	0.0006	0.0004	0.0004	0.0004	0.0004	0.0004				
IL10	rs1800896	0.0005	0.0004	0.0004	0.0004	0.0004	0.0004				
NOS2	rs9282799	0.0005	0.0004	0.0004	0.0004	0.0004	0.0004				
AJAP1	rs6674631	0.0005	0.0004	0.0004	0.0004	0.0004	0.0004				
NOS2	rs8078340	0.0005	0.0004	0.0004	0.0004	0.0004	0.0004				
CR1	rs17047660	0.0005	0.0004	0.0004	0.0004	0.0004	0.0004				
ICAM	rs5498	0.0005	0.0003	0.0004	0.0004	0.0004	0.0004				
TPTE2	rs182873742	0.0004	0.0003	0.0003	0.0003	0.0003	0.0003				
TNF	rs1800629	0.0004	0.0003	0.0003	0.0003	0.0003	0.0003				
RTN3	rs542998	0.0004	0.0003	0.0003	0.0003	0.0003	0.0003				
IL17RD	rs6780995	0.0003	0.0002	0.0002	0.0002	0.0002	0.0002				
CAND1	rs12307123	0.0003	0.0002	0.0002	0.0002	0.0002	0.0002				
PLEKHG1	rs114726617	0.0003	0.0002	0.0002	0.0002	0.0002	0.0002				
TLR6	rs5743810	0.0002	0.0001	0.0002	0.0002	0.0002	0.0002				
PLEKHG1	rs142712208	0.0003	0.0002	0.0002	0.0002	0.0002	0.0002				
PLEKHG1	rs2131263	0.0003	0.0002	0.0002	0.0002	0.0002	0.0002				
AJAP1	kgp15825649	0.0003	0.0002	0.0002	0.0002	0.0002	0.0002				
MKI67	rs115947774	0.0003	0.0002	0.0002	0.0002	0.0002	0.0002				
TNF	rs361525	0.0003	0.0002	0.0002	0.0002	0.0002	0.0002				
CTL4	rs2242665	0.0002	0.0002	0.0002	0.0002	0.0002	0.0002				
TNF	rs1800750	0.0002	0.0001	0.0002	0.0002	0.0002	0.0002				
TNF	rs3093662	0.0002	0.0002	0.0002	0.0002	0.0002	0.0002				
TLR6	rs5743809	0.0002	0.0002	0.0002	0.0002	0.0002	0.0002				
SDC1	rs11899121	0.0002	0.0002	0.0002	0.0002	0.0002	0.0002				
LTA	rs2239704	0.0002	0.0002	0.0002	0.0002	0.0002	0.0002				
IL13	rs20541	0.0002	0.0001	0.0002	0.0002	0.0002	0.0002				
TLR9	rs187084	0.0002	0.0001	0.0002	0.0002	0.0002	0.0002				
SNORD48	rs116288147	0.0002	0.0001	0.0001	0.0001	0.0001	0.0001				
IL4R	rs1805015	0.0002	0.0001	0.0001	0.0001	0.0001	0.0001				

PLEKHG1	rs15116938	0.0002	0.0001	0.0001	0.0001	0.0001	0.0001
RRM1	kgp12768002	0.0001	0.0001	0.0001	0.0001	0.0001	0.0001
CR1	rs17047661	0.0002	0.0001	0.0001	0.0001	0.0001	0.0001
SPTB	rs229587	0.0002	0.0001	0.0001	0.0001	0.0001	0.0001
PLEKHG1	rs76924464	0.0001	0.0001	0.0001	0.0001	0.0001	0.0001
PLEKHG1	rs151293197	0.0001	0.0001	0.0001	0.0001	0.0001	0.0001
PLEKHG1	rs79100774	0.0001	0.0001	0.0001	0.0001	0.0001	0.0001
LTA	rs909253	0.0001	0.0001	0.0001	0.0001	0.0001	0.0001
MKI67	rs148494166	0.0001	0.0001	0.0001	0.0001	0.0001	0.0001
NOS2	rs1800482	0.0001	0.0001	0.0001	0.0001	0.0001	0.0001
MKI67	rs11016116	0.0001	0.0001	0.0001	0.0001	0.0001	0.0001
ABO	rs8176750	0.0001	0.0001	0.0001	0.0001	0.0001	0.0001
HCG4	rs114980857	0.0001	0.0001	0.0001	0.0001	0.0001	0.0001
TNF	rs1799964	0.0001	0.0001	0.0001	0.0001	0.0001	0.0001
B3GALNT1	rs12107243	0.0001	0.0001	0.0001	0.0001	0.0001	0.0001
CD36	rs3211938	0.0001	0.0000	0.0001	0.0001	0.0001	0.0001
LPHN2	rs146428334	0.0001	0.0000	0.0000	0.0000	0.0000	0.0000
IL17RE	rs708567	0.0001	0.0000	0.0000	0.0000	0.0000	0.0000
NOS2	rs2297518	0.0001	0.0000	0.0000	0.0000	0.0000	0.0000
IL22	rs2227478	0.0000	0.0000	0.0000	0.0000	0.0000	0.0000
ATP2B4	rs3753036	0.0000	0.0000	0.0000	0.0000	0.0000	0.0000
NOD1	rs2075820	0.0000	0.0000	0.0000	0.0000	0.0000	0.0000
ABO	rs56390333	0.0000	0.0000	0.0000	0.0000	0.0000	0.0000
IL22	rs2227507	0.0000	0.0000	0.0000	0.0000	0.0000	0.0000
GABBR1	rs192151845	0.0000	0.0000	0.0000	0.0000	0.0000	0.0000
GAB1	rs7663712	0.0000	0.0000	0.0000	0.0000	0.0000	0.0000
FREM3	rs184895969	0.0000	0.0000	0.0000	0.0000	0.0000	0.0000
C6	rs1801033	0.0000	0.0000	0.0000	0.0000	0.0000	0.0000
ADCY9	rs10775349	0.0000	0.0000	0.0000	0.0000	0.0000	0.0000
FREM3	rs184908374	0.0000	0.0000	0.0000	0.0000	0.0000	0.0000
DARC	rs2814778	0.0000	0.0000	0.0000	0.0000	0.0000	0.0000
TLR4	rs4986791	0.0000	0.0000	0.0000	0.0000	0.0000	0.0000

*Estimates are the proportion of the total variance explained by each polymorphism individually when estimated on the scale shown ("Observed" where computed on the 0-1 scale and transformed to the underlying liability scale; "Logit" and "Probit" where computed using the logit and probit transformations to model them on the liability scale). Estimates from the case-control data ("Case-control") are inflated due to ascertainment bias and thus are adjusted to give the values expected in the general population ("General"). Estimates are missing for markers that had <5 genotypes carrying the minor allele. The model here included sex as a fixed effect but not ethnic group. When ethnic group was included, the estimates decreased by a factor of 0.91 on average. §Estimates when simultaneously fitting the most significant SNP for each of 12 genes explaining the most amount of variance individually under the logit model. Two SNPs for the ABO locus are included because they independently explained significant amounts of variance. The total variance explained by these 13 markers is shown in bold below these with adjustment for ascertainment bias.

Table S14: Proportions of variance explained by individual markers

		BlpI RFLP assay for the Dantu hybrid gene		
		**Non-Dantu**	**Dantu positive**	**Total**
**iPLEX assay for rs186873296**	AA	1685*	13	**1698**
	AG	17	213*	**230**
	GG	1	9*	**10**
	**Total**	**1703**	**235**	**1938**

All severe malaria cases (n=2244) were genotyped for the Dantu hybrid gene; data are shown for samples with both a valid rs186873296 and Dantu result. The BlpI RFLP assay does not distinguish readily between heterozygous and homozygous states for Dantu. RFLP=restriction fragment length polymorphism. *Concordant data between assays.

Table S15: **Concordance between iPLEX assay for rs186873296 and the BlpI RFLP assay for the Dantu hybrid in paediatric cases of severe malaria**

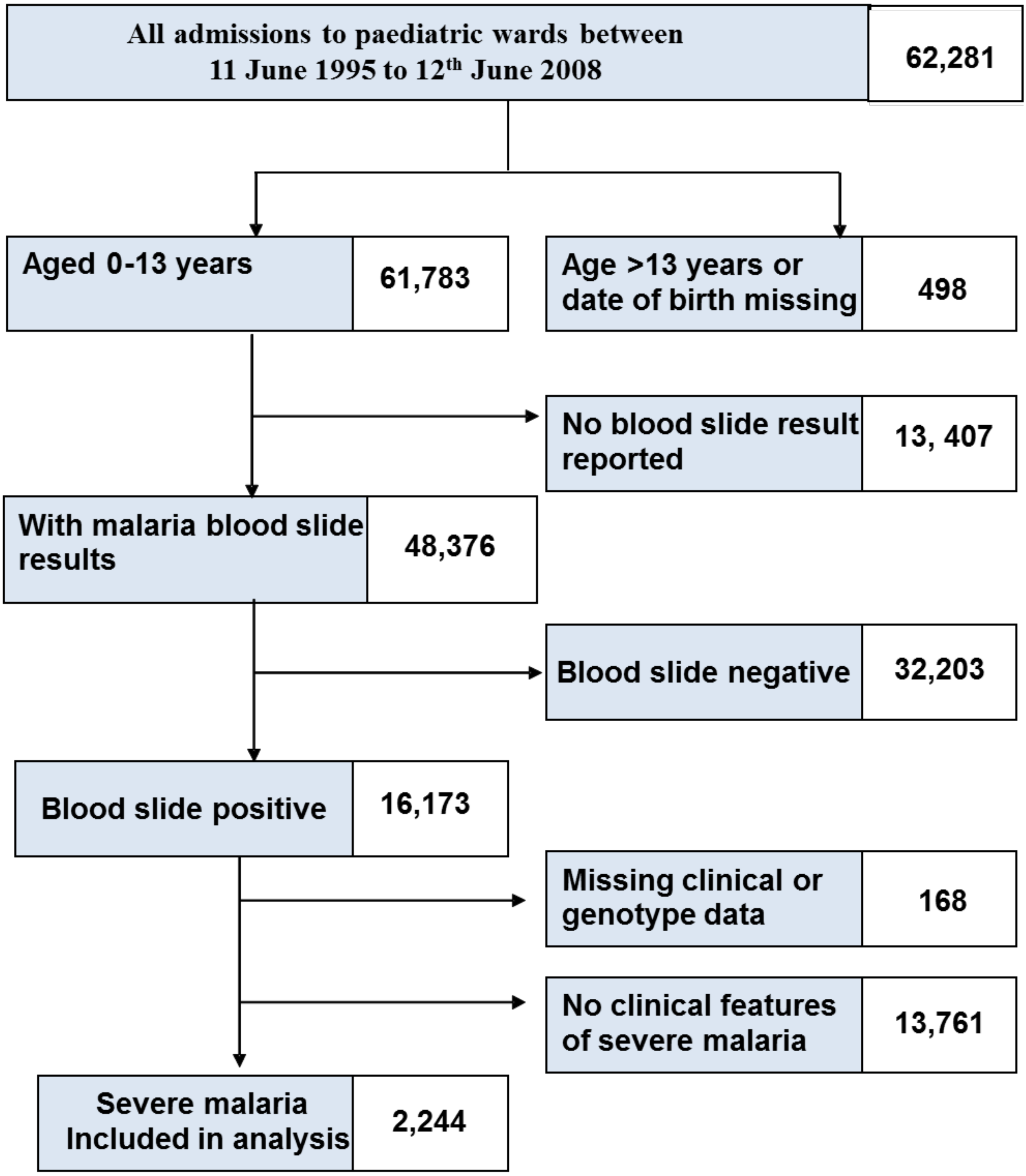

Figure S1: Flow diagram showing the selection of severe malaria cases.

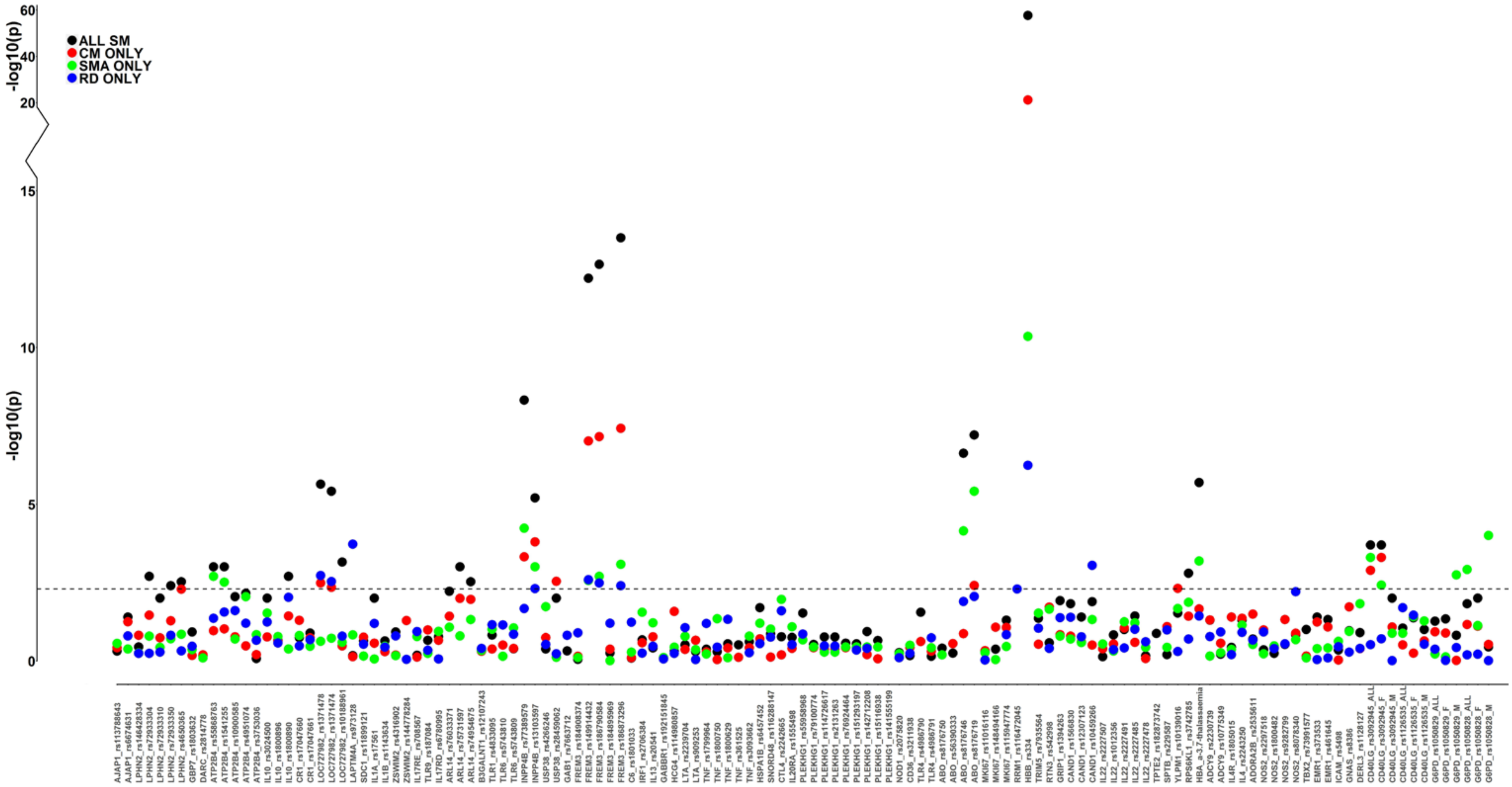

Figure S2. Manhattan plot showing the distribution of p-values for association tests with severe malaria phenotypes.
All SM; all individuals with any severe malaria phenotype; CM, cerebral malaria only; SMA, severe malaria anaemia only; RD, respiratory distress only. We tested for associations using additive (A), dominant (D), heterozygous (H), hemizygous (HM) and recessive (R) genetic models and the minimum p-value (as $-\log_{10}[p]$)for each polymorphism across the models is shown. Analyses were adjusted for gender, ethnic group and rs334 genotype. The dashed horizontal line represents the significance threshold of $p<0.005$ determined by permutation testing (see methods). Polymorphisms tested are listed in chromosomal order (see Supplementary Table 1 for details).

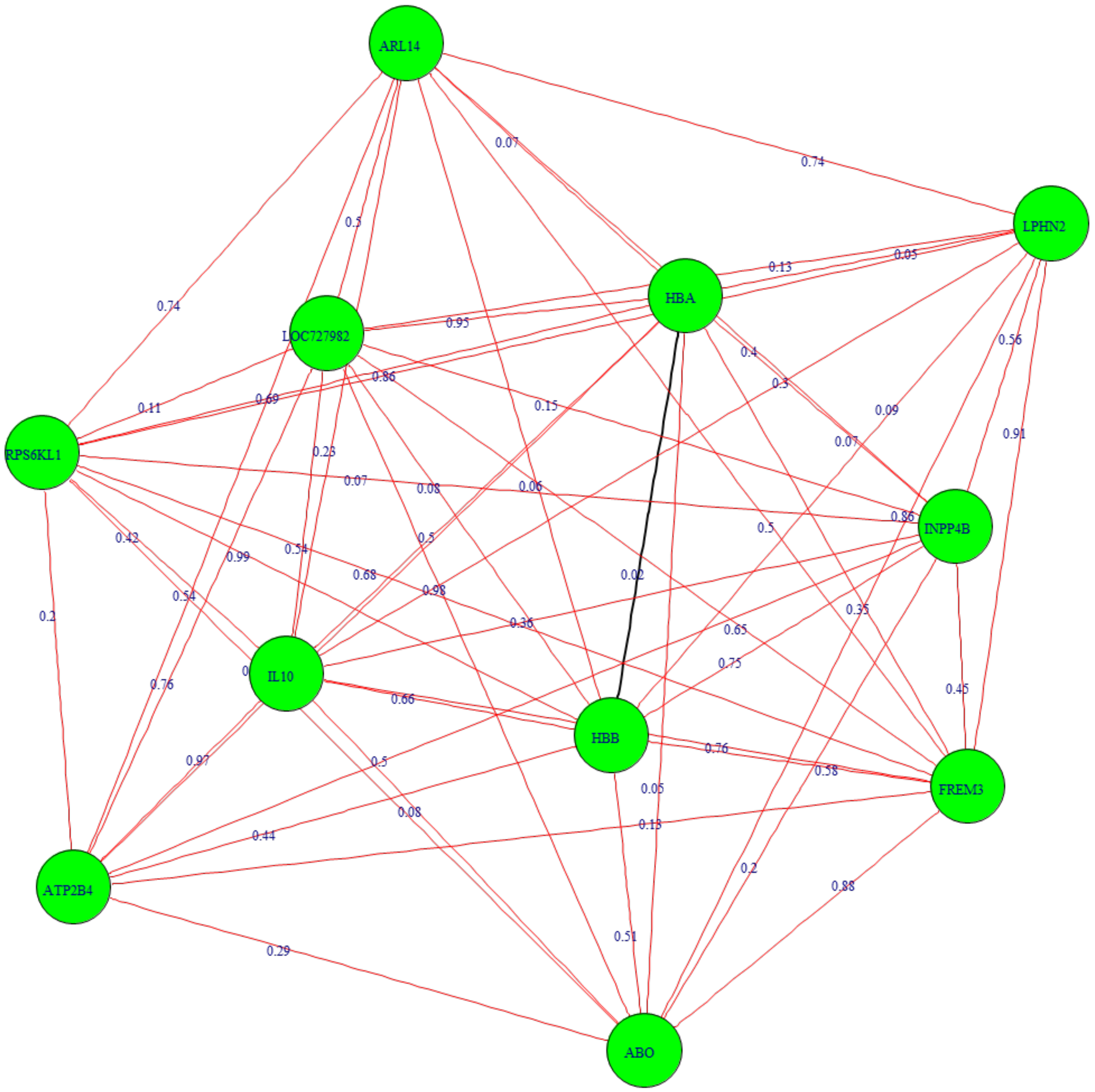

The figure shows a graphical representation of the data in Supplementary Table S12. The polymorphims used for the pairwise interaction analysis were those shown in the main text Table 2. The nodes in the figure are labelled according with the gene name for the polymorphims. The lines connecting the nodes show the p-values for the interactions. p-values were adjusted for ethnicity and gender.

Figure S3: Network diagram for interaction of polymorphim pairs in severe malaria.

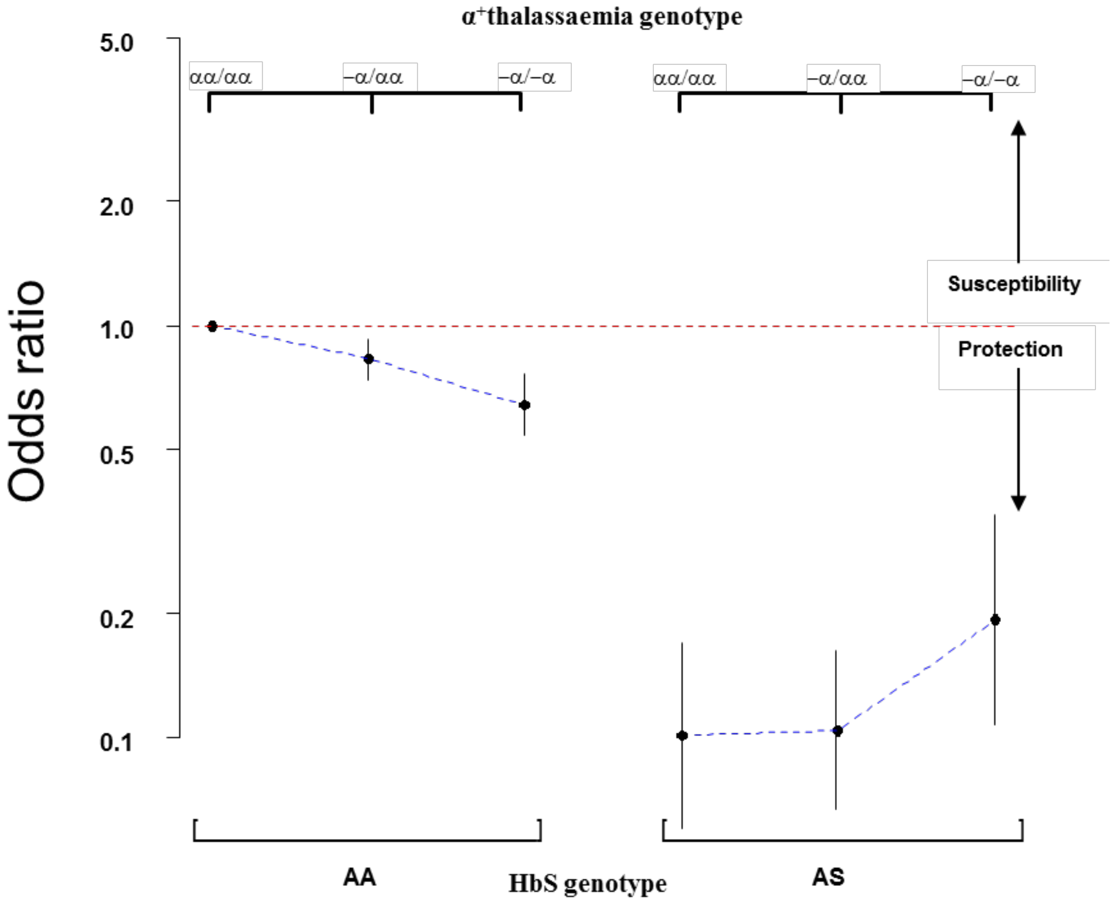

Allele group	a	b	c	d	e	f
Cases	828	1002	252	16	22	13
Controls	1131	1649	537	216	288	92
P-value	ref	0.002	5.8×10^{-7}	3.1×10^{-18}	1.4×10^{-23}	4.0×10^{-8}

For each combination of genotypes (x-axis), we computed the odds ratio ± 95% CI (y-axis) and p-value relative to the reference group (α^{+}thalassaemia: αα/αα/HbAA). The red line shows the point of no effect. The odds ratio has been plotted on a log scale so that the distances above and below 1 represent the same size of effect although opposite effect. We also list the sample size for cases and controls. Allele group "a" shows the baseline for normal α^{+}thalassaemia and HbAA. As shown in allele groups "a", "b" and "c", on a normal HbAA background, α^{+}thalassaemia tends to protect reaching an OR of 0.6 in the homozygote state. However, this trend for protection is not seen in individuals with HbAS, indeed the α^{+}thalassaemia locus reduces the effect of HbAS (OR 0.1 to 0.2).

Figure S4: Two-way epistatic interactions between rs334 (HbS) and $\alpha^{-3.7}$-thalassaemia for all severe malaria cases.

A

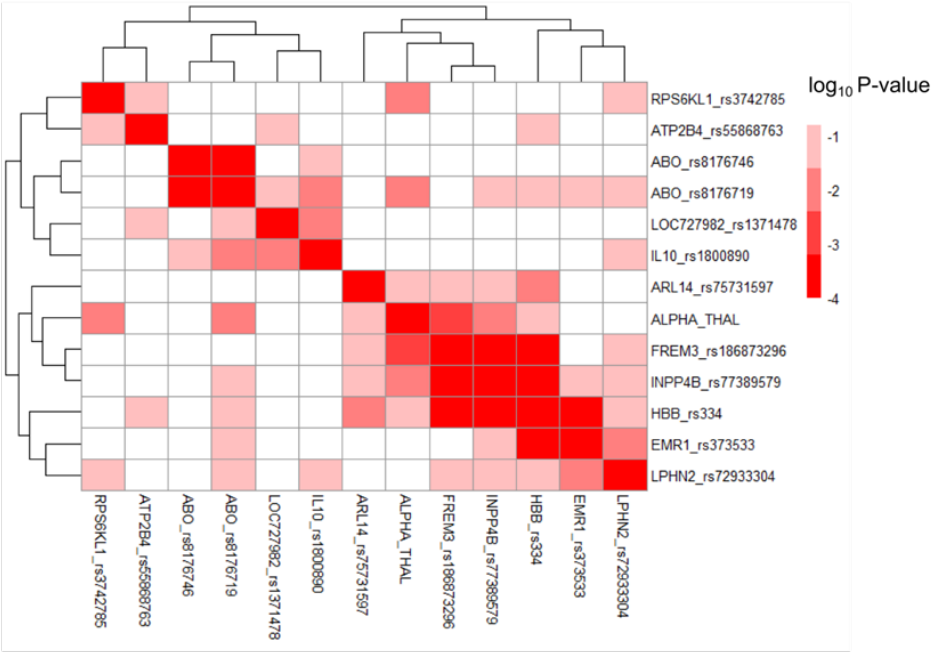

B

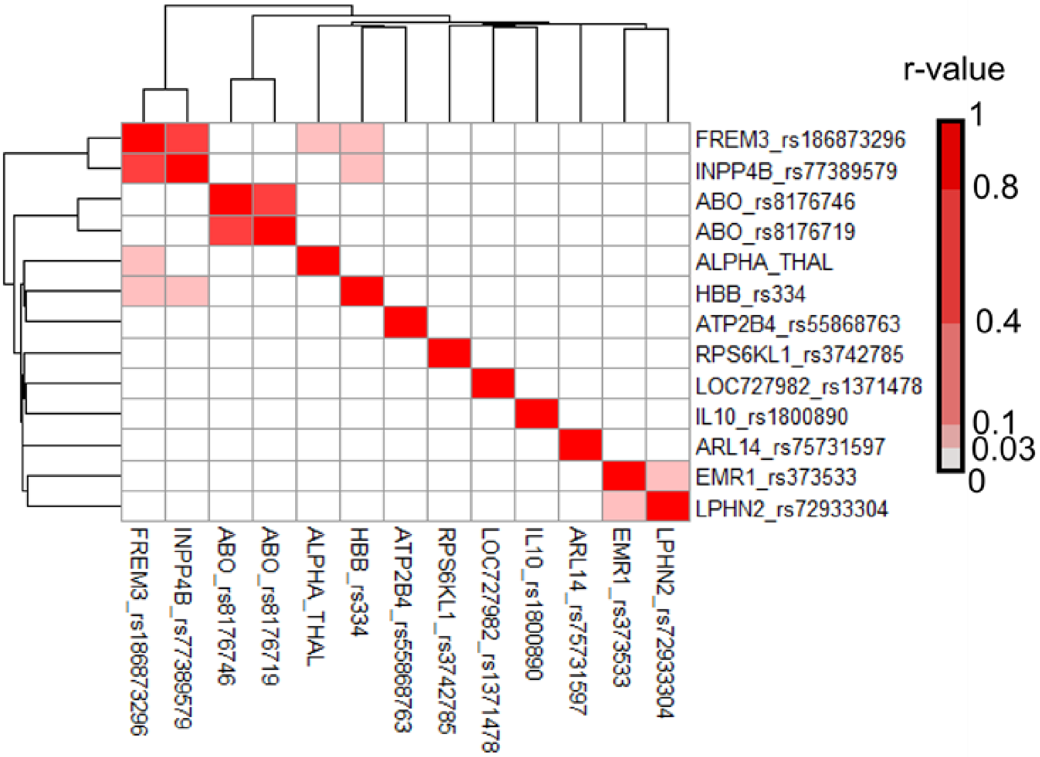

Panels A and B show the p-value and r-value for each pairing of the 13 polymorphims (Figure 3 and Supplementary Table 14) explaining the variability in severe malaria among individuals in the general population (one SNP per gene except for ABO where both rs8176719 and 8176746 independently explained significant amounts of variance).
A: p-value: Colour indicates significance of linkage disequilibria by χ^2 test. polymorphim pairs are clustered and ordered according to closeness of p-values.
B: r-value: Colour indicates the strength of the correlation between genotypes (r-value). Values of r > 0.03 are highly significant by χ^2 test (P<0.001). polymorphim pairs are clustered and ordered according to relatedness of r-values.

Figure S5. Linkage disequilibria among the 13 polymorphims explaining most of the variance in the population.

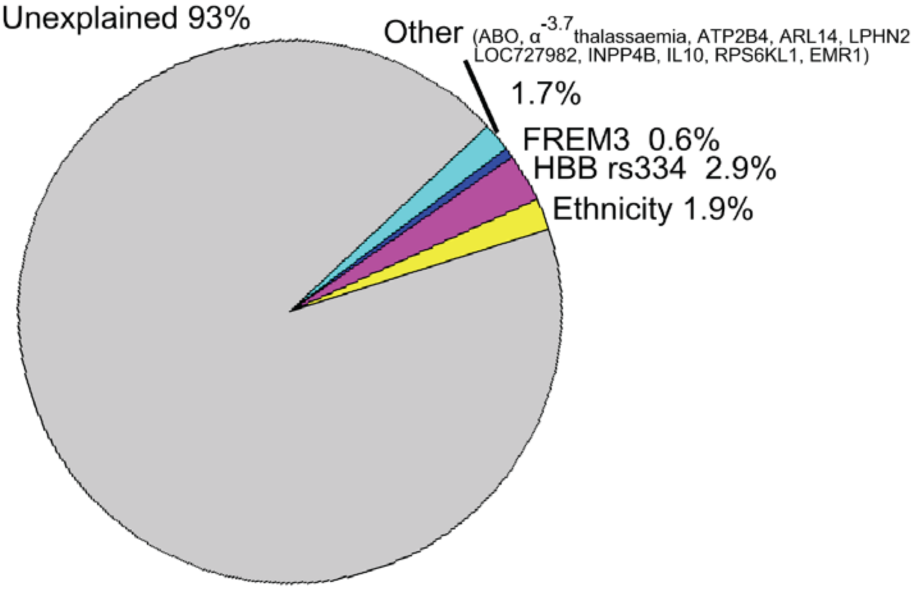

Figure S6. Proportion of variance in the risk of severe malaria among children that is explained by genetic polymorphisms and other factors. Values pertain to the general population and were obtained by analysing on the observed (0-1) scale and then transforming to the underlying liability scale (see Supplementary Table 12).

MalariaGEN Consortium members

This list can also be viewed at;
https://www.malariagen.net/projects/consortial-project-1/malariagen-consortium-members

MalariaGEN Resource Centre

Wellcome Centre for Human Genetics, University of Oxford, Oxford, UK; The Big Data Institute, University of Oxford, Oxford, UK and The Wellcome Sanger Institute, Hinxton, Cambridgeshire, UK

Aaron Vanderwal, Abier Elzein, Aceme Nyika, Alieu Mendy, Alistair Miles, Andrea Diss, Angeliki Kerasidou, Angie Green, Anna E. Jeffreys, Bronwyn MacInnis, Catherine Hughes, Catherine Moyes, Chris C.A. Spencer, Christina Hubbart, Cinzia Malangone, Claire Potter, Daniel Mead, David Barnwell, Dominic P. Kwiatkowski, Dushyanth Jyothi, Eleanor Drury, Elilan Somaskantharajah, Eliza Hilton, Ellen Leffler, Gareth Maslen, Gavin Band, George Busby, Geraldine M. Clarke, Ioannis Ragoussis, Jacob Almagro Garcia, Jane Rogers, Jantina deVries, Jennifer Shelton, Jiannis Ragoussis, Jim Stalker, Joanne Rodford, John O'Brien, Julie Evans, Kate Rowlands, Katharine Cook, Kathryn Fitzpatrick, Katja Kivinen, Kerrin Small, Kimberly J. Johnson, Kirk A. Rockett, Lee Hart, Magnus Manske, Marilyn McCreight, Marryat Stevens, Matti Pirinen, Meike Hennsman, Michael Parker, Miguel SanJoaquin, Nuno Sepúlveda, Olivia Cook, Olivo Miotto, Panos Deloukas, Rachel Craik, Rebecca Wrigley, Renee Watson, Richard Pearson, Robert Hutton, Samuel Oyola, Sarah Auburn, Shivang Shah, Si Quang Le, Sile Molloy, Susan Bull, Susana Campino, Taane G. Clark, Valentín Ruano-Rubio, Victoria Cornelius, Yik Ying Teo.

National Institute for Biological Standards, South Mimms, Hertfordshire, UK

Patrick Corran, Nilupa Silva, Paul Risley.

The Wellcome Trust, London, UK

Alan Doyle.

Bernhard-Nocht-Institut für Tropenmedizin, Hamburg, Germany

Jennifer Evans, Rolf Horstmann.

Howard Hughes Medical Institute, University of Maryland, Baltimore, Maryland, USA

Chris Plowe.

National Institute of Allergy and Infectious Diseases, Bethesda, Maryland, USA

Patrick Duffy.

Foundation for the National Institutes of Health, Bethesda, Maryland, USA

Dan Carucci, Michael Gottleib.

The Institut Pasteur de Dakar, Dakar, Senegal and The Institut Pasteur de Dakar, Paris, France

Adama Tall, Alioune Badara Ly, Amagana Dolo, Anavaj Sakuntabhai, Odile Puijalon.

Medical Research Council Laboratories, Banjul Gambia

Abdou Bah, Abdoulie Camara, Abubacar Sadiq, Aja Abie Khan, Amie Jobarteh, Anthony Mendy, Augustine Ebonyi, Bakary Danso, Bintou Taal, Climent Casals-Pascual, David J. Conway, Emmanuel Onykwelu, Fatoumatta Sisay-Joof, Giorgio Sirugo, Haddy Kanyi, Haddy Njie, Herbert Obu, Horeja Saine, Idrissa Sambou, Ismaela Abubakar, Jalimory Njie, Janet Fullah, Jula Jaiteh, Kalifa A. Bojang, Kebba Jammeh, Kumba Sabally-Ceesay, Lamin Manneh, Landing Camara, Lawrence Yamoah, Madi Njie, Malick Njie, Margaret Pinder, Mariatou Jallow, Mohammed Aiyegbo, Momodou Jasseh, Momodou Lamin Keita, Momodou Saidy-Khan, Muminatou Jallow, Ndey Ceesay, Oba Rasheed, Pa Lamin Ceesay, Pamela Esangbedo, Ramou Cole-Ceesay, Rasaq Olaosebikan, Simon Correa, Sophie Njie, Stanley Usen, Yaya Dibba.

University Of Bamako, Bamako Mali

Abdoulaye Barry, Abdoulaye Djimdé, Abdourahmane H. Sall, Amadou Abathina, Amadou Niangaly, Awa Dembele, Belco Poudiougou, Elizabeth Diarra, Kariatou Bamba, Mahamadou A. Thera, Ogobara Doumbo, Ousmane Toure, Salimata Konate, Sibiry Sissoko, Mahamadou Diakite.

Centre National De Recherche Et De Formation Sur Le Paludisme, Ouagadougou Burkina Faso

Amadou T. Konate, David Modiano, Edith C. Bougouma, Germana Bancone, Issa N. Ouedraogo, Jaques Simpore, Sodiomon B. Sirima, Valentina D. Mangano, Marita Troye-Blomberg.

Noguchi Memorial Institute For Medical Research With Navrongo Health Research Centre, Navrongo, Ghana

Abraham R. Oduro, Abraham V. O. Hodgson, Anita Ghansah, Francis Nkrumah, Frank Atuguba, Kwadwo A. Koram, Lucas N. Amenga-Etego, Michael D. Wilson, Nana Akosua Ansah, Nathan Mensah, Patrick A. Ansah, Thomas Anyorigiya, Victor Asoala, William O. Rogers.

Kwame Nkrumah University Of Science And Technology, Kumasi, Ghana

Alex Osei Akoto, Alex Owusu Ofori, Anthony Enimil, Daniel Ansong, David Sambian, Emmanuel Asafo-Agyei, Justice Sylverken, Sampson Antwi, Tsiri Agbenyega.

University Of Ibadan, Ibadan, Nigeria

Adebola E. Orimadegun, Folakemi Anjol Amodu, Olajumoke Oni, Olayemi O. Omotade, Olukemi Amodu, Subulade Olaniyan.

University Of Buea, Buea, Cameroon

Andre Ndi, Clarisse Yafi, Eric Akum Achidi, Eric Mbunwe, Judith Anchang-Kimbi, Regina Mugri, Richard Besingi, Tobias O. Apinjoh, Vincent Titanji.

University Of Khatoum, Khartoum, Sudan

Ahmed Elhassan, Ayman Hussein, Hiba Mohamed, Ibrahim Elhassan, Muntaser Ibrahim.

KEMRI-Wellcome Research Programme, Kilifi, Kenya

Gilbert Kokwaro, Tom Oluoch, Alexander Macharia, Carolyne M. Ndila, Charles Newton, Daniel H. Opi, Dorcas Kamuya, Evasius Bauni, Kevin Marsh, Norbert Peshu, Sassy Molyneux, Sophie Uyoga, Thomas N. Williams, Vicki Marsh.

Joint Malaria Programme, Kilimanjaro Christian Medical Centre And London School Of Hygiene And Tropical Medicine, Moshi, Tanzania

Alphaxard Manjurano, Behzad Nadjm, Caroline Maxwell, Chris Drakeley, Eleanor Riley, Frank Mtei, George Mtove, Hannah Wangai, Hugh Reyburn, Sarah Joseph.

National Institute For Medical Research, Tanga, Tanzania

Deus Ishengoma, Martha Lemnge, Theonest Mutabingwa.

Muhimbili University Of Health And Allied Sciences Of The University Of Dar Es Salaam, Dar Es Salaam, Tanzania

Julie Makani, Sharon Cox.

Blantyre Malaria Project With Malawi-Liverpool-Wellcome Programme, Blantyre, Malawi

Ajib Phiri, Annie Munthali, David Kachala, Labes Njiragoma, Malcolm E. Molyneux, Mike Moore, Neema Ntunthama, Paul Pensulo, Terrie Taylor, Vysaul Nyirongo.

The University of Colombo, Colombo, Sri Lanka

Richard Carter, Deepika Fernando, Nadira Karunaweera, Rajika Dewasurendra.

Mahidol University, Bangkok, Thailand

Prapat Suriyaphol, Pratap Singhasivanon.

Oxford University Clinical Research Unit, Ho Chi Minh City, Vietnam

Cameron P. Simmons, Cao Quang Thai, Dinh Xuan Sinh, Jeremy Farrar, Ly Van Chuong, Nguyen Hoan Phu, Nguyen T. Hieu, Nguyen Thi Hoang Mai, Nguyen Thi Ngoc Quyen, Nicholas Day, Sarah J. Dunstan, Sean E. O'Riordan, Tran Thi Hong Chau, Tran Tinh Hien.

Papua New Guinea Institute For Medical Research, Madang, Papua New Guinea

Angela Allen, Enmoore Lin, Harin Karunajeewa, Ivo Mueller, John Reeder, Laurens Manning, Moses Laman, Pascal Michon, Peter Siba, Stephen Allen, Timothy M. E. Davis.
